# Ligand Chirality Transfer from Solution State to the Crystalline Self‐Assemblies in Circularly Polarized Luminescence (CPL) Active Lanthanide Systems

**DOI:** 10.1002/advs.202307448

**Published:** 2024-03-06

**Authors:** David F. Caffrey, Tumpa Gorai, Bláithín Rawson, Miguel Martínez‐Calvo, Jonathan A. Kitchen, Niamh S. Murray, Oxana Kotova, Steve Comby, Robert D. Peacock, Patrycja Stachelek, Robert Pal, Thorfinnur Gunnlaugsson

**Affiliations:** ^1^ School of Chemistry and Trinity Biomedical Sciences Institute (TBSI) Trinity College Dublin The University of Dublin Dublin 2 Ireland; ^2^ Departamento de Química Inorgánica, Facultade de Química Campus Vida Universidade de Santiago de Compostela Santiago de Compostela 15782 Spain; ^3^ Chemistry, Institute of Natural and Mathematical Sciences Massey University Auckland 0632 New Zealand; ^4^ AMBER (Advanced Materials and Bioengineering Research) Centre Trinity College Dublin The University of Dublin Dublin 2 Ireland; ^5^ School of Chemistry University of Glasgow Glasgow G12 8QQ Scotland; ^6^ Department of Chemistry Durham University Durham DH1 3LE UK; ^7^ Present address: Department of Polymers and Functional Materials CSIR‐Indian Institute of Chemical Technology Hyderabad 500007 India

**Keywords:** circularly polarized luminescence (CPL), coordination chemistry, lanthanide luminescence, pyridyl‐diamide (pda), self‐assembly

## Abstract

The synthesis of a family of chiral and enantiomerically pure pyridyl‐diamide (**pda**) ligands that upon complexation with europium [Eu(CF_3_SO_3_)_3_] result in chiral complexes with metal centered luminescence is reported; the sets of enantiomers giving rise to both circular dichroism (CD) and circularly polarized luminescence (CPL) signatures. The solid‐state structures of these chiral metallosupramolecular systems are determined using X‐ray diffraction showing that the ligand chirality is transferred from solution to the solid state. This optically favorable helical packing arrangement is confirmed by recording the CPL spectra from the crystalline assembly by using steady state and enantioselective differential chiral contrast (EDCC) CPL Laser Scanning Confocal Microscopy (CPL‐LSCM) where the two enantiomers can be clearly distinguished.

## Graphical abstract

Enantiopure formation of chiral self‐templated lanthanide supramolecular structures gives rise to CPL when probed both in solution and the solid state demonstrating chirality transfer.

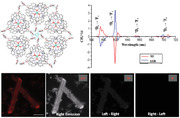

## 1 Introduction

**Scheme 1:**
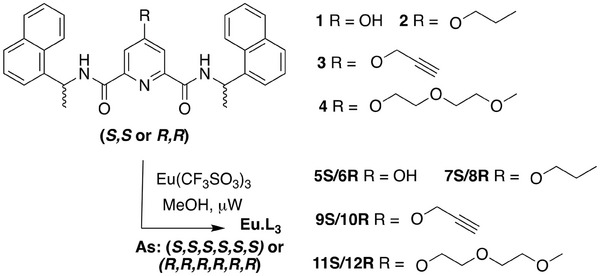
Chemical structures of the chiral **pda** ligands **1‐4** synthesised herein, and their corresponding (1:3) Eu(III) complexes **5S‐12R** (Eu:L_3_) where **R** = (*R*,*R*,*R*,*R*,*R*,*R*) and **S** = (*S*,*S*,*S*,*S*,*S*,*S*) are the stereochemistry at each of the three chiral ligands (**1‐4**) in **Eu.L_3_**.

The development of chiral supramolecular discrete or higher order self‐assembled structures from chiral or achiral ligands has attracted significant attention in recent times.^[^ ^1^, ^2^ ^]^ The additional use of metal ions in directing the formation of these assemblies is particularly intriguing, as this leads to obtaining structures with unique physical properties and complex architectures.^[^ ^3^, ^4^, ^5^ ^]^ We have been interested in the synthesis of supramolecular structures where complexes such as helicates and other higher‐order assemblies, as well as soft materials, have been prepared from chiral ligands and lanthanide ions.^[^ ^6^ ^]^ This includes using Eu(III), Tb(III), Sm(III) ions, etc. to direct the synthesis of chiral ligands derived from either pyridyl‐diamide (**pda**)^[^ ^7^ ^]^ or 2,6‐bis(1,2,3‐triazol‐4‐yl)pyridine (**btp**)^[^ ^8^ ^]^ structures; resulting in the formation of lanthanide luminescent systems with rich structural and functional properties.^[^ ^9^ ^]^ Others have demonstrated alternative examples, such as knots and mechanically interlocked molecules, as elegantly shown by Leigh and co‐workers,^[^ ^10^ ^]^ while several examples of lanthanide helicates, cages, and polyhedral structures have been developed by Clever et al.,^[^ ^11^ ^]^ Law et al.,^[^ ^12^ ^]^ Sun et al.,^[^ ^13^ ^]^ and Piguet et al.^[^ ^14^ ^]^ to name just a few.^[^ ^15^ ^]^ Herein, we present the synthesis of the chiral **pda** ligand **1** (as an intermediate) and the derivatives **2–4**, possessing either *(S,S)* or *(R,R)* stereochemistry, that are functionalized at the 4th position of the pyridine unit as shown in **Scheme** **1** with short chains. We study the properties of these derivatives in solution using Eu(III) ion, which upon complexation (and with the aid of the antenna effect)^[^ ^16^ ^]^ emits at long‐wavelengths with line‐like emission bands and long‐lived excited state lifetimes.^[^ ^17^ ^]^ Moreover, we explore the solid state properties of these chiral self‐assemblies which demonstrate that the chirality of the ligand is transferred to the lanthanide complexes both in solution, as well to their crystalline state. We observe this by probing the circularly polarized luminescence (CPL),^[^ ^18^, ^19^, ^20^ ^]^ which is a powerful means of detecting “helical” luminescence from chiral molecules; the presence of the europium being particularly attractive where all the ^5^D_0_ → ^7^F*_J_* (*J* = 1–4) transitions can be probed. We demonstrate this both in solution, as well as by recording the CPL from the single crystal of chiral crystalline assembly using CPL microscopy,^[^ ^21^ ^]^ which opens various application possibilities.^[^ ^22^, ^23^ ^]^

## 2 Results and Discussion

The synthesis of ligands **2–4** *(S,S or R,R)* (See Schemes S1 and S2 and full experimental details, Supporting Information) was achieved from ligand **1**, which was formed by reacting commercially available chelidamic acid with the appropriate enantiomer of 1‐(1‐naphthyl)‐ethylamine using peptide coupling reactions giving **1** *(S,S)* and **1**(*R,R)* in 63% and 56% yield, respectively. The 4‐hydroxy group was then reacted with either 1‐bromopropane, propargyl bromide or 1‐iodo‐2‐(2‐methoxy‐ethoxy)ethane, respectively, under anhydrous conditions giving ligands **2–4** (*S,S or R,R* enantiomers), as white solids in yields of 32–67%. The products were fully characterized which included the use of NMR experiments and HRMS analysis.

Gratifyingly, clear plate‐shaped single crystals of the *(R,R)* polyethoxy‐substituted enantiomer **4** *(R,R)* suitable for X‐ray crystal structure analysis were grown by recrystallization from EtOH. The ligand was found to crystallize in the chiral orthorhombic space group P2_1_2_1_2_1_, and contained two ligand molecules, along with two interstitial EtOH molecules in the asymmetric unit. The two amine hydrogen atoms face into the cavity of the ligands and hydrogen bond to the pyridyl nitrogen atom (Supporting Information).

These ligands were used then for the synthesis of the Eu(III) complexes **5S‐12R**, which was achieved using microwave irradiation, where the relevant ligands **2–4** (*S,S* or *R,R*) were heated in the presence of 0.33 eq. of Eu(CF_3_SO_3_)_3_ for 20 min in MeOH (Supporting Information). The Eu(III) **5S** and **6R** were formed under reflux from intermediate **1** (*SS/RR*) in the presence of 0.33 eq. of Eu(CF_3_SO_3_)_3_. This stoichiometry fulfills the nine‐coordinate requirement of Eu(III), resulting in the formation of the 1:3 (Eu:L) complexes **5S‐12R** (i.e., **Eu.L_3_**, where **L** = **1–4**, *S*,*S* or *R*,*R*). This gives rise to complexes with six stereogenic centers (i.e., all *S* or all *R*, respectively depending on the ligand used). Vapor diffusion of diethyl ether into the reaction solution precipitated the complexes as a white crystalline solid in yields ranging from 68–82%. The successful Eu(III) complexation was determined by ^1^H NMR, HRMS, IR, and elemental analysis. The ^1^H NMR (400 MHz, CD_3_OD‐*d*_4_) spectra of all complexes displayed characteristic broadening and shifting due to the paramagnetic nature of Eu(III) (Supporting Information). As with their corresponding ligands, the ^1^H NMR spectra were found to be identical for both enantiomers in a given pair of structures. In addition to elemental analysis, high‐resolution mass spectrometry (HRMS) analysis (MALDI^+^) also showed the desired 1:3 (M:L) stoichiometry, where in all cases, the experimental isotopic distribution patterns matching those of the calculated ones (Supporting Information).

**Figure 1:**
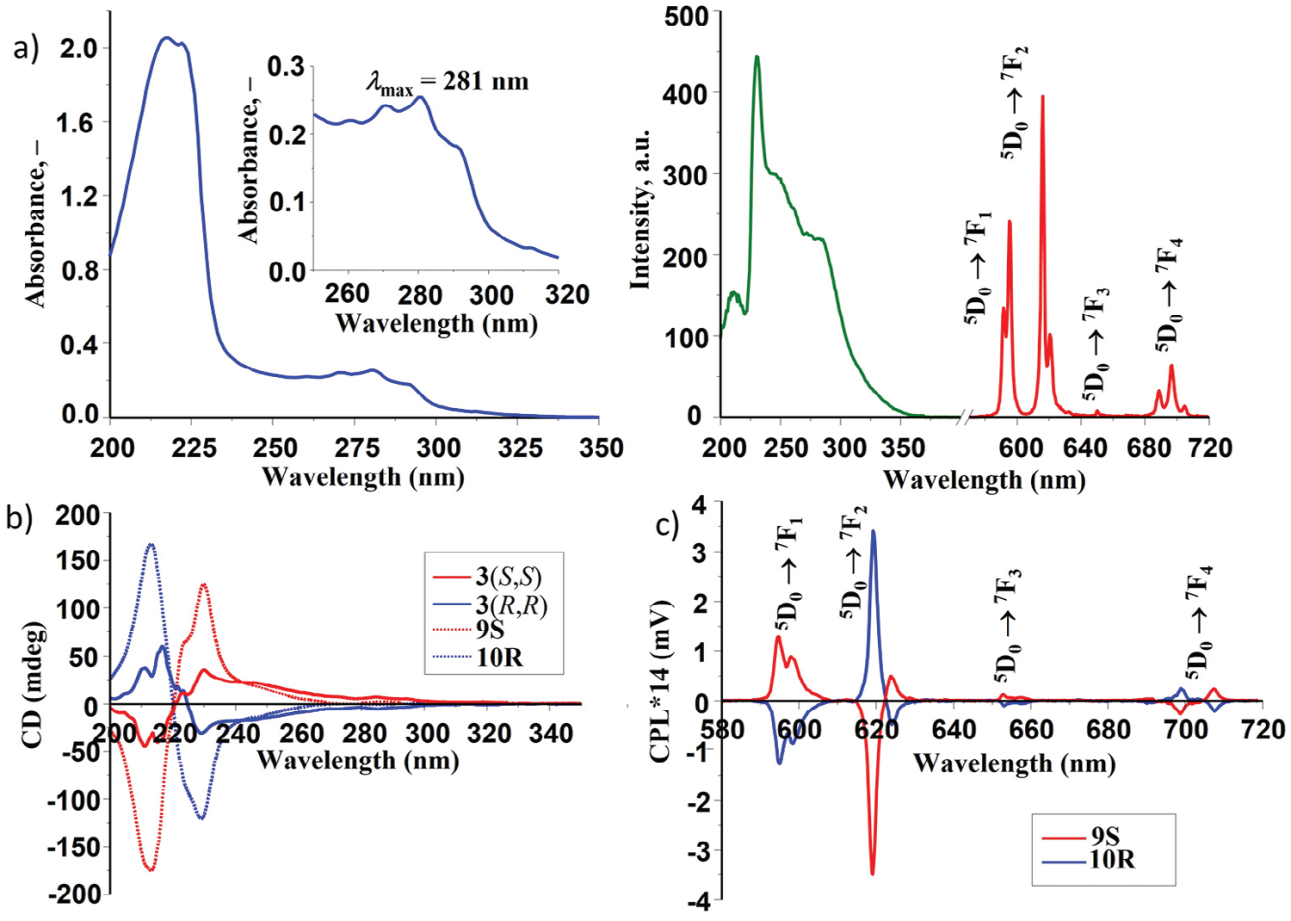
a) The absorption (blue), phosphorescence excitation (green) and emission (red) spectra (*λ*_ex_ = 281 nm) of **9S** (c = 6.4 × 10^−6^ m) recorded in MeOH at 298 K. b) CD spectra of ligand solutions of **3**(*S*,*S*) and **3**(*R*,*R*) (c = 2.0 × 10^−5^ m) recorded in MeCN at 298 K before (solid lines) and after (dashed lines) the addition of 0.33 eq. of Eu(CF_3_SO_3_)_3_ to give the complexes **9S** and **10R**, respectively. c) CPL emission spectra of **9S** and **10R** recorded in MeCN at 298 K (*λ*_ex_ = 281 nm). (See Supporting Information).

The photophysical properties of these Eu(III) complexes were evaluated, both in MeOH as well as in MeCN solutions. For all, the UV–vis absorption spectra were comprised of two main bands, centered at 220 and 281 nm the latter arising from the *π*–*π* ^*^ transitions of the naphthalene units, as demonstrated in **Figure** **1**a (left). The naphthalene moieties (along with the **pda** center) also act as sensitizing antennae, allowing for the population of the Eu(III) ^5^D_0_ excited state. This was confirmed by excitation at 281 nm, as red Eu(III)‐centered emission was observed for all complexes, with characteristic line‐like emission bands for the ^5^D_0_ → ^7^F*_J_* (*J* = 1–4) transitions; this is shown in Figure 1a (right) for **9S** (see Supporting Information for other complexes), which also shows the corresponding excitation spectrum, demonstrating the sensitization of the Eu(III) excited state by a ligand. Notable by its absence in the Eu(III) emission spectra (recorded in the phosphorescence mode) was the electronic‐dipole *J* = 0 transition; a band appearing only when symmetry is lost and the Laporte selection rules are relaxed.^[^ ^9a,c^ ^]^ This indicated high symmetry for these complexes. The quantum yield (See Supporting Information) as well as the Eu(III) excited state lifetimes of all the complexes were determined. The latter allows for the hydration states (*q*) to be determined in both H_2_O (*τ*_O‐H_) and D_2_O (*τ*_O‐D_), as well as in CH_3_OH (*τ*_O‐H_) and CD_3_OD (*τ*_O‐D_). All excited state decays were best fit to a mono‐exponential decay from which *q* values of 0 were calculated for each of the complexes, confirming the coordination number of Eu(III) ion being nine (see Supporting Information). Furthermore, spectroscopic titrations using **2–4** (*S,S* or *R,R*) and Eu(CF_3_SO_3_) in either MeOH or CH_3_CN were also carried out, and equilibrium binding constants determined using non‐linear regression analysis (*c.f*. Supporting Information for ligand **4** as an example). These showed the 1:3 stoichiometry formed in high yield even under kinetic control for all the complexes (See Supporting Information). The quantum yield for the Eu(III) emission of all the complexes was also determined (See Supporting Information).

Due to the chiral nature of the ligands used, the CD spectra of the ligands **1–4** and the Eu(III) complexes (**5S‐12R**) were recorded in MeCN to verify the enantiomeric relationship (Supporting Information). Indeed, the CD spectra revealed exciton signals of opposite sign and approximately equal magnitude for a given pair of complexes, signifying the formation of enantiomers under diastereoselective control. This is demonstrated in Figure 1b for both ligand **3** *(S,S* or *R,R)* and for the corresponding Eu(III) complexes **9S** and **10R** (see Supporting Information for other complexes). The observed lower ellipticity values for **11S** and **11R** in comparison to the other complexes are most likely due to the lower percentage of tris‐complex species formed in solution under kinetic control. Indeed, their binding constants values are lower in comparison to other self‐assemblies studied here. An impact of kinetic effect due to the presence of polyethoxy chain in **4**(*S*,*S* or *R*,*R*) is also possible here (Supporting Information). In these pairs of enantiomers, the handedness at the Eu(III) center is either Δ or Λ. As the complexes give rise to delayed Eu(III) luminescence with sharp transitions, the CPL was also recorded in MeCN (*λ*_ex_ = 281 nm). The resulting spectra again confirmed the enantiomeric nature of these complexes, where the CPL signals of opposite sign for the ^5^D_0_ → ^7^F*_J_* (*J* = 1–4) transitions were recorded reaffirming the existence of optically active supramolecular species of opposing chirality, as depicted in Figure 1c for **9S** and **10R**, respectively. Here, a large spectral uniformity was observed across the six complexes, where these formed from the ligands (*S*) enantiomers all gave rise to a positive band for the Δ*J* = 1 transition (split at 595 and 598 nm) and a large negative band corresponding to the hypersensitive Δ*J* = 2 transition centered at 619 nm (with a positive split at 624 nm). Conversely, the opposite effect was observed for the (*R*) enantiomeric species. The luminescence dissymmetry factor (*g_lum_*) for the Δ*J* = 1 and 2 showed good agreement across the three pairs of enantiomers (Supporting Information).^[^ ^24^ ^]^ With *g*_lum_ values of +0.27 and −0.27 for the Δ*J* = 1 transitions in the **9S** and **10R**, while *g*_lum_ values of −0.23 and +0.21 were calculated for Δ*J* = 2; the slightly larger *g*_lum_ values for Δ*J* = 1 being attributed to the magnetic‐dipole character of the transition, which is more likely to yield large circular polarization.^[^ ^25^ ^]^ Such relatively high *g*_lum_ values can be rationalized as a combination of two main factors, namely the chirality of the ligand and the topological chirality from the helical ligand arrangement, with both contributing to the total CPL activity.

**Figure 2:**
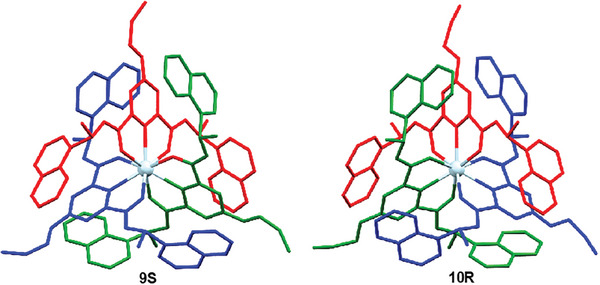
Perspective views of the asymmetric units of the Eu(III) complexes **9S** and **10R** [formed from ligand **3** (*S,S*) and **3** (*R,R*), respectively], showing that the chirality at the ligand dictating the stereochemistry around the metal ion where **9S** was assigned as Δ, while **10R** gave the Λ stereochemistry. Hydrogen atoms are omitted for clarity.

**Figure 3:**
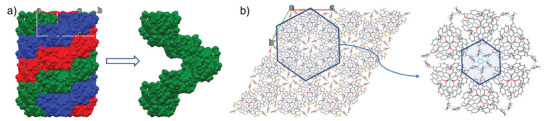
a) X‐ray packing diagram (space‐filled) of **11S** propagated down the *c*‐axis showing the helical packing arrangement of complex molecules and focusing on a single complex strand only, b) X‐ray packing diagram (capped stick) of **11S** viewed down the *c*‐axis showing both the presence of channels and the orientation of the ligand polyethoxy chains into these cavities focusing on the intermolecular interactions established in the internal cavity between the polyethoxy chains.

The above results effectively demonstrate that the chirality of the complexes is driven by asymmetric induction, a mechanism whereby the metal stereochemistry (Δ or Λ) is directed by the absolute configuration of the chiral antennae, which would be expected to be transferred into their larger crystalline self‐assembly. To support this, we grew crystals of six of these complexes that were suitable for X‐ray crystal structure analysis. All the analyzed complexes showed the same coordination environments around the Eu(III) ions. This included the pair of enantiomers **9S** and **10R** shown in **Figure** **2** (demonstrating the structures being mirror images of each other; See Supporting Information for the enantiomers **5S** and **6R**), where the ligands are arranged in a helical fashion around the Eu(III) center, and the propargyl substituents are faced away from the metal centers. The packing of these systems (Supporting Information) also demonstrates that the helical nature of the complexes is extended into the three dimensions, even in the case of **11S**, possessing the longer 2‐(2‐methoxy‐ethoxy)ethane chain at the back of the pyridyl units (**Figure** **3**), which could potentially disturb helical packing of the complexes in the solid state (See Supporting Information for similar packing diagrams for **10R**).

The packing diagram of **11S** is shown in Figure 3, but all the crystal structures obtained show the same general features (*c.f*. Supporting Information). The structural data for **11S** was resolved to an R factor of 5.36 (See Supporting Information for the remaining structures). **11S** crystallized in the *P6_3_* space group (Supporting Information, with the extended packing analysis shown in Figure 3a). Here, as in the case of either **9S** and **10R**, the Eu(III) is coordinating all three ligands through their central pyridine nitrogen atom and carbonyl oxygen atoms to afford a nine‐coordinate tri‐capped trigonal prismatic N_3_O_6_ geometry. In these, each of the pyridine rings is intercalated between the naphthyl groups of the other two ligands, enabling *π*–*π* stacking interactions across the three ligands that contributed to the overall stability of the complexes (see Supporting Information). The pyridyl substituents extended outwards away from the Eu(III) cores, effectively underlining the suitability of this position for functionalization without negatively altering the overall architecture. It is also clear that the ligand induces a helical arrangement around the Eu(III) center, in which the ligands are wrapped around the metal, resulting in the creation of the chiral metal ion complexes. The metal stereochemistry of **11S** could be assigned as Δ, while the *R*‐analogs gave the Λ stereochemistry (see Supporting Information). This Δ and Λ chirality is transferred to the packing order of **11S**, as shown in Figure 3a,b, when viewed down the crystallographic *c*‐axis. In addition to the helical wrapping of ligands within the complex molecules, the latter themselves were found to pack in a helical arrangement (Figure 3a). This packing is also the product of the formation of intermolecular hydrogen bonds between the amide NH protons and the oxygen atoms of the CF_3_SO_3_^−^ counter‐anions as well as non‐classical CH⋅⋅⋅F interactions between the F atoms with the naphthalene rings, Figure 3b and Supporting Information. This gives rise to the formation of channels for **11S** running down the *c*‐axis with the side chains all orientated into the channel cavities, Figure 3b. Furthermore, the 2‐(2‐methoxy‐ethoxy)ethoxy chains oriented toward the cavity, interact through CH⋅⋅⋅CH soft contacts of the C45 and two other C45 carbons from the neighboring complexes. In the lower level of the packing, another six complexes interact through the same atoms in a starred disposition, giving rise to a six‐star view along *c*‐axis of the packing (see Supporting Information). The overall chirality‐induced helicity of the complex is thus retained. Such an arrangement was found for all of the complexes formed from Eu(CF_3_SO_3_)_3_.

**Figure 4:**
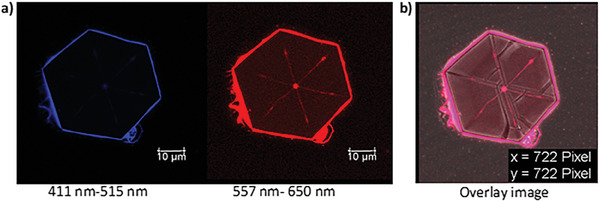
a) The confocal fluorescence microscopic images from the hexagonal crystals of **9S** deposited from MeCN, the emission was recorded in two channels. b) The overlayed emission from both channels.

We next investigated if the sensitized Eu(III) centered emission and CPL signature could be observed from this crystalline material and focused on the complexes **9S** and **10R**. Under UV excitation, the crystalline samples were clearly red emitting to the naked eye. Further investigations, using confocal fluorescence microscopy, demonstrated this at the microscopic crystalline level, the imaging of hexagonal plates, **Figure** **4**, showing emission from the surface of crystals of **9S**. To demonstrate that this was due to the Eu(III) emission, the spectra were also extracted from confocal microscopy images, which showed the transitions consistent with characteristic sharp 615 nm emission band of Eu(III) (Supporting Information).^[^ ^22a^ ^]^

**Figure 5:**
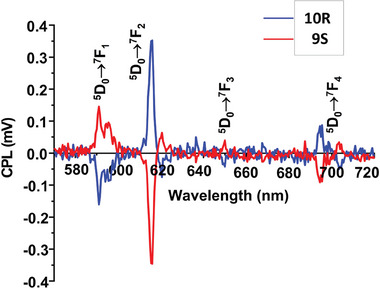
The CPL spectra recorded from the solid film samples of **9S** and **10R**, *λ*_ex_ = 325 nm, 50 averages collected.

Having established the solid state Eu(III) emission from these samples, we investigated if the chiral emission could be recorded as well.^[^ ^26^ ^]^ However, to achieve this, it was necessary to prepare solid state samples by dissolving the crystalline materials in methanol solution (c = 1 mg mL^−1^), which was subsequently drop‐casted on glass cover slips. The result was a formation of single crystals, which allowed for the CPL to be recorded, **Figure** **5**. It should be noted that only one layer of single crystals was obtained, and no crystals above or below were detected. This was confirmed by Laser Scanning Confocal Microscopy (LSCM) where the optical sectioning capability (at 355 nm excitation, x40 0.7NA objective, AU = 0.6) is 610 nm. The crystals were localized within a radius of 0.4 mm. Even though the solid‐state emission spectra are not of the same resolution as those shown in Figure 1c for the complexes in solution, the samples display the same CPL signals of opposite sign for the *ΔJ* = 1, 2, 3 and 4 transitions. The CPL spectra were recorded utilizing a photoelastic modulator (PEM) CPL spectrometer and *g*_lum_ were determined as −0.3 and +0.3 for *ΔJ* = 2 transitions in **9S** and **10R**, respectively, in a 45° orientation. These results are in excellent agreement with the *g*_lum_ values that were determined in the solution for the same set of complexes (see above). The reason for the difference in the resolution and intensity (overall detectable circularly polarized brightness (CPB)) of the CPL spectra for complexes **9S** and **10R** in solution (Figure 1c) and solid state (Figure 5) is the fact that the measurement of the CPL of Eu(III) complexes in the solid state is much more challenging as the concentration quenching may occur which in turn results in the lower resolution of the CPL spectra. For the solid‐state measurements, the excitation area is much smaller compared to the experiment performed for the solution. However, we positioned the glass coverslip, containing a single layer of crystals, in the beam path of an instrument that is designed for a solution state of a 1 × 1 cm cuvette with an 2.3 mm diameter excitation beam orthogonal to the detection path.

**Figure 6:**
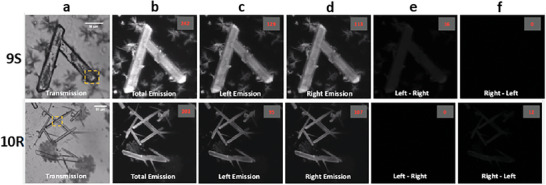
EDCC CPL‐LSCM of **9S** (*top*) and **10R** (*bottom*) on a glass substrate. a) Transmission image, b) total europium emission (*λ*_ex_= 355 nm, 20 mW, *λ*_em_ = 594/10 nm), c,d) left and right‐handed CPL channel respectively (*λ*_em_ = 594/10 nm), e) left‐handed EDCC image (left CPL–right CPL), f) right‐handed EDCC image (right CPL–left CPL). The objective used: ×40 0.7 NA air, 210 × 210 µm FOV, 100 AVG, 1.5 µm AXIAL SECTION, the whole dimensions of the crystal have been captured (length × width × depth). Scale bars = 50 µm, numbers in red are avg. Eight‐bit pixel intensity values for each image region.

Having demonstrated the CPL from the solid crystals, we subsequently used the aforementioned samples and utilized the recently developed technology of CPL‐LSCM.^[^ ^21^, ^27^ ^]^ We used a modified LSCM (SP5 II, Leica Microsystems) with excitation provided by a fiber coupled 80 mW variable power 355 nm Nd:YAG CW laser using a high transmission 594/10 nm bandpass filter selected for emission from the *ΔJ* = 1 band of Eu(III). *ΔJ* = 1 is the ideal manifold to focus on as it is the only band with exclusively negative or positive monosigned CPL signal, **Figure** **6**. The simultaneously recorded microscopy images of the emitted left and right handed CPL light have been processed to provide Enantioselective Differential Chiral Contrast (EDCC) imaging as demonstrated by Stachelek et al.^[^ ^21^ ^]^ The results from these imaging experiments are shown in Figure 6 (see Supporting Information for further examples), where the crystals of both enantiomers are compared; the top panel demonstrating the results from **9S** while **10R** is shown for comparison in the bottom panel. Here, the observation of the chiral nature of these crystals is made possible by the EDCC imaging by subtraction of the simultaneously recorded left‐handed CPL image from the right‐handed CPL (and vice versa) using ImageJ software (v1.49). For better visualization of both EDCC L‐R and R‐L images we enhanced their contrast by 40% and provided these images in the Supporting Information as the values in the red are unequivocal proof of EDCC measurement that is directly translated to CPL signal and more importantly helicity dominance. The EDCC analysis is of ground‐breaking quality from a measurement of one crystal and demonstrates the underlying potential of this new technique. From this work, we can conclude that the PEM‐CPL results show excellent correspondence with the CPL‐LSCM data confirming that both **9S** and **10R** have discernible CPL and CPB properties in the solid state. This has not been demonstrated before using such self‐assembled lanthanide complexes, proving that the chiral nature of the organic ligands is transferred, not only to the single Eu(III) complexes in solution but to their solid states too.^[^ ^28^ ^]^

## 3 Conclusion

In conclusion we have reported two exciting developments. First, the reported work shows that CPL technique can be used to prove that Eu(III) materials with modest photoluminescence quantum yield and *g*_lum_ values in solution can self‐assemble into enantiopure crystals with superior CPL and *g*_lum_ values. Second and even more excitingly the CPL technology, and specifically CPL‐LSCM, has evolved significantly to allow these crystals to be examined in detail by recording EDCC images. Combined, the aforementioned developments open new exciting avenues toward the development of CPL‐OLEDs and displays amongst others. Opening new avenues into CPL research of self‐assembled supramolecular systems.

Experimental Details can be found in Supporting Information.

## Conflict of Interest

The authors declare no conflict of interest.

## Author Contributions

The manuscript was written through the contributions of all authors. The synthesis, characterization, and self‐assembly studies of the compounds reported were carried out by N.S.M., D.F.C., O.K., S.C., R.D.P., P.S., and R.P. X‐ray crystallography was carried out by M.M.‐C. and J.A.K. T.G., O.K., and R.P. designed and supervised the project. All authors have approved the final version of the manuscript.

## Supporting information

Supporting Information

Supporting Information

## Data Availability

The data that support the findings of this study are available from the corresponding author upon reasonable request.
