# Supplementary material for: Ligand Chirality Transfer from Solution State to the Crystalline Self‐Assemblies in Circularly Polarized Luminescence (CPL) Active Lanthanide Systems
Source: Adv Sci (Weinh). 2024 Mar 6;11(18):2307448. doi: 10.1002/advs.202307448 (PMC11095229; doi:10.1002/advs.202307448)
Supplement: Supplementary file 1 — Supporting Information [file ADVS-11-2307448-s002.pdf]

ADVANCED SCIENCE
Open Access

Supporting Information

for *Adv. Sci.*, DOI 10.1002/advs.202307448

Ligand Chirality Transfer from Solution State to the Crystalline Self-Assemblies in Circularly Polarized Luminescence (CPL) Active Lanthanide Systems

David F. Caffrey, Tumpa Gorai, Bláithín Rawson, Miguel Martínez-Calvo, Jonathan A. Kitchen, Niamh S. Murray, Oxana Kotova, Steve Comby, Robert D. Peacock, Patrycja Stachelek, Robert Pal* and Thorfinnur Gunnlaugsson**

Ligand chirality transfer from solution state to the crystalline self-assemblies in circularly polarised luminescence (CPL) active lanthanide systems

Supporting Information

General Experimental Details

All chemicals were purchased from Sigma-Aldrich Ireland Ltd., Acros Organics, and TCI Ltd., and were used without further purification, unless otherwise stated. Deuterated solvents for NMR analysis were all obtained from Apollo Scientific. Dry solvents were prepared in accordance with standard procedures described by Vogel, with distillation prior to each use.[1] Chromatographic columns were performed manually using either silica gel 60 (230-240 mesh ASTM) or aluminium oxide (activated, neutral, Brockman I STD grade, 150 mesh). Chromatographic columns were also run on a Teldyne Isco Combiflash Companion automatic machine using pre-packed silica or alumina columns. Thin-layer chromatography (TLC) was conducted using both Merck Kiesegel 60 F254 silica plates and Polygram Alox N/UV254 aluminium oxide plates, and observed by UV light or developed in an iodine chamber. Melting Points were determined using an Electrothermal IA900 digital melting point apparatus.

NMR spectra were recorded using either a Bruker Spectrospin DPX-400 instrument, operating at 400.1 MHz for ^{1}H NMR and 100.6 MHz for ^{13}C NMR, or a Bruker AV-600 instrument, operating at 600.1 MHz for ^{1}H NMR and 150.2 MHz for ^{13}C NMR. All NMR spectra were measured at 293 K. Chemical shifts are expressed in parts per million (ppm or δ) relative to the non-deuterated solvent peak and, for ^{1}H NMR spectra, are reported alongside the number of protons, splitting pattern, coupling constant where applicable, and proton assignment (in that order). Multiplicities are abbreviated as follows; singlet (*s*), doublet (*d*), triplet (*t*), quartet (*q*), multiplet (*m*), and broad (*br*).

Electro mass spectra were recorded using a Mass Lynx NT V 3.4 on a Waters 600 controller connected to a 996 photodiode array detector, with HPLC grade carried solvents. Accurate molecular weights were determined by a peak-matching method, using leucine enkephaline (H-Tyr-Gly-Gly-Phe-Leu-OH) as the

standard reference (m/z = 556.2771); all accurate mass were reported within ± 5 ppm of the expected mass. Infrared spectra were recorded on a Perkin Elmer Spectrum One FT-IR spectrometer fitted with a universal ATR sampling accessory. Elemental analysis was carried out at the Microanalysis Laboratory, School of Chemistry and Chemical Biology, University College Dublin.

UV-vis absorption and luminescence spectroscopy

Using a 1.0 cm path length quartz cell, UV-vis absorption and luminescence spectra were recorded using a Varian CARY 50 and a Varian Cary Eclipse spectrophotometer, respectively. The solvents utilised were of either HPLC or spectrophotometric grade. The concentrations of the ligands and complexes analysed were the same for both UV-vis and luminescence titrations. All measurements were performed at 298 K, with this maintained using a thermostated unit block. Baseline correction measurements were employed for all UV-vis spectra, where the blank was a sample of the solvent system in which the titration was performed. Excitation spectra were recorded by monitoring the emission at the maximum emission wavelength of Eu(III) emission - 616 nm, and also recorded in delayed (phosphorescence) mode

Luminescence lifetime measurements

The luminescence lifetime measurements for the synthesised Eu(III) complexes were conducted using a Varian Carey Eclipse Fluorimeter in time-resolved mode at 298 K. All lifetime values were obtained from an average of six independent measurements, each recorded with a different gate time in the range 0.020 – 0.045 ms.

Quantum yield determination

The luminescence quantum yields (Q_L^{Ln}) were determined by relative method using $Cs_3[Eu.(dpa)_3]{\cdot}9H_2O$ complex in 0.1 M Tris buffer (pH = 7.45) (Q_L^{Ln} = 24.0 ± 0.25%) as the standard to which the absorbance and emission intensity of the samples were compared according to Equation:[2,3]

$$Q_x = Q_{std} \times \left(\frac{A_{std}\,(\lambda_{std})}{A_x\,(\lambda_x)}\right) \times \left(\frac{I_{std}\,(\lambda_{std})}{I_x\,(\lambda_x)}\right) \times \left(\frac{n_x^2}{n_{std}^2}\right) \times \left(\frac{E_x}{E_{std}}\right)$$

The estimated error for the quantum yield values is ± 10%.

Circular dichroism and circularly polarised luminescence measurements

CD spectra were recorded on a Jasco J-810-150S spectropolarimeter. Each CD trace represents an average of three scans. CPL spectra were recorded in solution by calibration of the emission monochromator managed by passing scattered light from a low power HeNe laser through the detection system. This optical detection system consisted of a photoelastic modulator (PEM, Hinds Int.) operating at 50 kHz and a linear polariser, which together combine to act as a circular analyser, followed by a long pass filter, focusing lens, and a 0.22 m double monochromator. The emitted light was detected by a cooled EMI-9558QB photomultiplier tube operating in photon counting mode. The 50 kHz reference signal from the photoelastic modulator was used to direct the incoming pulses into two separated counters. An up counter, which counts every photon pulse and, therefore, is a measure of the total luminescence signal ($I = I_L + I_R$), and an up-down counter, which adds pulses when the analyser is transmitting left-handed circularly polarised light and subtracts pulses when the analyser is transmitting right-handed circularly polarised light. The second counter then provides a measure of the differential emission intensity ($\Delta I = I_L - I_R$).

X-ray crystallographic characterisation

X-ray data was collected on either a Rigaku Saturn 724 CCD Diffractometer using graphite-monochromated MoKα radiation (α = 0.71073 Å) or a Bruker APEX II CCD Diffractometer using graphite-monochromated MoKα radiation (α = 0.71073 Å) or CuKα radiation (α = 1.54178 Å). Depending on the instrument used, the datasets were collected using either CrystalClear (Rigaku) or APEX2 (Bruker) software; with data integration, reduction, and correction for absorption and polarisation effects, as well as space group determinations, all performed using this software. The structures were solved by direct methods (SHELXS-97) and refined against all F2 data (SHELXS-97)[4] with the exception of OH and NH protons, all H atoms were positioned geometrically and refined using

riding model with d(CH) = 0.95 Å, Uiso = 1.2 Ueq (C). OH and NH protons were found from the difference map and fixed to the attached atoms with UH = 1.2 UX.

CPL spectroscopy

PEM-CPL spectrometer:[5] CPL was measured with a home-built (modular) spectrometer. The excitation source was a broad band (200 – 1000 nm) laser-driven light source EQ 99 (Elliot Scientific). The excitation wavelength was selected by feeding the broadband light into an Acton SP-2155 monochromator (Princeton Instruments); the collimated light was focused into the sample cell (1 cm quartz cuvette). Sample PL emission was collected perpendicular to the excitation direction with a lens (f = 150 mm). The emission was fed through a photoelastic modulator (PEM) (Hinds Series II/FS42AA) and through a linear sheet polariser (Comar). The light was then focused into a second scanning monochromator (Acton SP-2155) and subsequently on to a photomultiplier tube (PMT) (Hamamatsu H10723 series). The detection of the CPL signal was achieved using the field modulation lock-in technique. The electronic signal from the PMT was fed into a lock-in amplifier (Hinds Instruments Signaloc Model 2100). The reference signal for the lock-in detection was provided by the PEM control unit. The monchromators, PEM control unit and lock-in amplifier were interfaced to a desktop PC and controlled by a custom-written Labview graphic user interface. The lock-in amplifier provided two signals, an AC signal corresponding to (I_L- I_R) and a DC signal corresponding to (I_L + I_R) after background subtraction. The emission dissymmetry factor was therefore readily obtained from the experimental data, as 2 AC/DC.

Spectral calibration of the scanning monochromator was performed using a Hg-Ar calibration lamp (Ocean Optics). A correction factor for the wavelength dependence of the detection system was constructed using a calibrated lamp (Ocean Optics). The measured raw data was subsequently corrected using this correction factor. The validation of the CPL detection systems was achieved using light emitting diodes (LEDs) at various emission wavelengths. The LED was mounted in the sample holder and the light from the LED was fed through a broad band polarising filter and $\lambda/4$ plate (Ocean Optics) to generate circularly

polarised light. Prior to all measurements, the $\lambda/4$ plate and a LED were used to set the phase of the lock-in amplifier correctly. The emission spectra were recorded with 0.5 nm step size and the slits of the detection monochromator were set to a slit width corresponding to a spectral resolution of 0.25 nm. CPL spectra (as well as total emission spectra) were obtained through an averaging procedure of several scans. The CPL spectra were smoothed using a shape-preserving Savitzky-Golay smoothing (polynomial order 5, window size 9 with reflection at the boundaries) to reduce the influence of noise and enhance visual appearance; all calculations were carried out using raw spectral data. Analysis of smoothed vs raw data was used to help to estimate the uncertainty in the stated gem factors, which was typically ±10%.

SS-CPL spectrometer:[6] Sample excitation was provided by a 2.35 W 365 nm LED with a 9 nm spectral half-width [CUN6AF4A; Roithner LaserTechnik] mounted in a custom-built heat sink. Power to the LED was supplied by a bench-top power supply [PL303QMD 30V/3A; Aim TTI] operating in constant current mode. A collimating lens, ground-glass diffuser [N-BK7, Thorlabs], and a 240 – 395 nm bandpass filter [FGUV5, Thorlabs] placed prior to the sample ensured that excitation light was diffuse, unpolarised, and constrained to $\lambda < 400$ nm. Samples were placed within an enclosed holder [CVH100, Thorlabs]. All samples were measured in quartz cuvettes with a 10 mm by 10 mm cross-section [111-10-40; Hellma Analytics]. Sample emission was collected 90° to the excitation beam. The emitted light passed through an achromatic QWP [AQWP05M-600; Thorlabs], which converted circularly polarised light into two orthogonal, linearly polarised, signals, corresponding to L-CPL and R-CPL. The light then was then split into two spatially separated detection channels by a non-polarising 50/50 beam splitter [BS013; Thorlabs]. Each detection channel was capable of independently measuring L-CPL and R-CPL in a sequential manner by precisely rotating a linear polariser [LPVISE100-A; Thorlabs] mounted in a motorized precision rotation mount [PRM1/MZ8; Thorlabs] controlled via an electronic controller [KDC101; Thorlabs]. A long pass filter ($\lambda > 450$ nm) [FEL0450, Thorlabs] prior to detection ensured no stray excitation light could reach the detector. Emission intensities of left CPL and right CPL channels were quantified

by two matched high-sensitivity SS charge coupled device (CCD) spectrometer operating at 400 – 800 nm with ~0.2 nm sampling increments [Ocean Optics Maya2000Pro, H3 grating, 350 – 850 nm]. To maximise light throughput to the CCD detector, the spectrometer entrance aperture slit was removed.

From the intensity of each channel, the total emission $(I_{L-CPL} + I_{R-CPL})$, CPL $(I_{L-CPL} - I_{R-CPL})$ spectra and g_{em} (emission dissymmetry factor) $g_{em} = \frac{2(I_{L-CPL} - I_{R-CPL})}{(I_{L-CPL} + I_{R-CPL})}$ were calculated. Synchronized operation of both channels enabled rapid concurrent acquisition of full CPL spectra. SS-CPL operation was automated using custom-written LabVIEW programs [LabVIEW 2013, National Instruments].[7] Data was analysed post-hoc with custom-written Matlab programs [Matlab 2019a, MathWorks].

Time-resolved detection of whole-spectra was achieved by introducing a time delay between the pulsed LED excitation and the start of SS-CCD spectrometer acquisition. LED pulsation and spectrometer acquisition were synchronised to transistor–transistor logic (TTL) signals produced via a USB multifunction I/O device [USB-6210, National Instruments]. Acquisition at a rate of 43 Hz was found to minimize spurious noise (*e.g.* from mains frequency electronics at 50 Hz) whilst providing a suitable measurement window for time-resolved measurement of long-lived lanthanide emission (*i.e.* several milliseconds) with a 10-millisecond integration time.

Circular Polarisation Luminescence Laser Scanning Confocal Microscope (CPL-LSCM)[7]

CPL-LCSM was enabled by adapting a commercial LSCM (SP5 II, Leica Microsystems) with excitation provided by a fibre coupled 80 mW variable power 355 nm Nd:YAG CW laser using a x63, 1.40 NA LambdaBlue objective.[8] The CPL analysis module was external to an output port and all elements were mounted in a 30 mm cage mount system for optimal alignment (assorted 30 mm components, Thorlabs). First, light from the sample focal plane excites the mirror controlled X1 emission port and passes through a high transmission bandpass filter (it is BP589/10 and BP595/10 nm [Edmunds Optics, 65162 and 86737]) mounted in a switchable filter selector apparatus (CFS1/M, Thorlabs). These

bandpass filters selected for emission from the ΔJ = 1 emission band of Europium. The circularly polarised emission is then converted to linearly polarised light by an achromatic quarter wave plate (AQWP05M-600, Thorlabs) and is separated into two detection arms by a simple 50/50 beam-splitter cube (BS013, Thorlabs). In each arm, the linearly polarised light is selectively analysed by linear polarisers (LPVISE100-A, Thorlabs) mounted within ultra-high precision computer-controlled rotation mounts with ± 60 μrad unidirectional repeatability (K10CR1/M, Thorlabs), orientated to select for left or right CPL states via computer control software (Kinesis, Thorlabs). The intensity of emission in each path was quantified by fibre-coupled (200 micron) high performance matched tandem avalanche photodiodes (Leica ADPs, Becker & Hickl ID-120). The two detection arms were aligned to achieve matched sensitivity to enable rapid simultaneous acquisition of left and right CPL images. Calibration of the linear polarisers for enantioselective localisation was executed based upon the procedure reported in Mackenzie *et al.*[6]

Confocal Microscopy

Leica SP8 STED confocal microscopy with a 40X oil immersion lens was used to visualize the crystals. Image analysis was performed using Leica Application Suite software. Crystals were excited by a 405 nm argon laser, emission 411-515 nm and 557-650 nm. Images are representative of three independent experiments.

Confocal microscopy, and CPL sample preparation procedure:

For confocal imaging, propargyl substituted **dpa**(*S*,*S*)-Eu(III) complex (**9S** derivative) (1mg/mL) solution (20 μL) in both methanol and acetonitrile were dropcasted on Ibidi 8 well glass slide and images were recorded in Leica SP8 scanning confocal microscopy.

For circularly polarized luminescence (CPL) measurement, **9S** and **10R** (20 μL) solutions in methanol (1mg/mL) were dropcasted on a coverslip and air-dried.

Synthesis

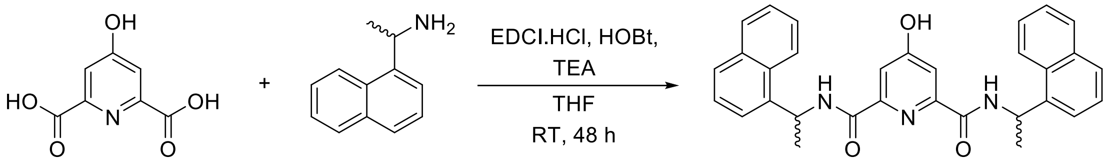

***Scheme S1:** Synthesis of ligand **1**(S,S or R,R) using either S or R stereochemistry for the naphthalene.*

4-Hydroxy-*N,N'*-bis((*S*)-1-(naphthalen-1-yl)ethyl)pyridine-2,6-dicarboxamide 1*(S,S)*

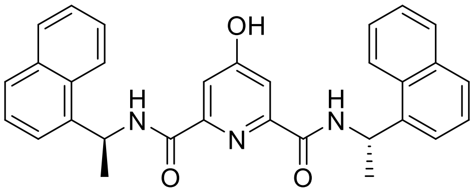

To a stirred solution of (*S*)-(-)-1-(1-naphthyl)-ethyl-amine (1.96 g, 1.84 mL, 11.47 mmol, 2.10 eq.) in anhydrous THF (35 mL), HOBt (1.48 g, 10.92 mmol, 2.00 eq.) and chelidamic acid (1.00 g, 5.46 mmol, 1.00 eq.) were added. The solution was stirred for 30 min at 0 °C before addition of EDCI·HCl (2.15 g, 11.19 mmol, 2.05 eq.) and triethylamine (1.10 g, 1.52 mL, 10.92 mmol, 2.00 eq.), with stirring for a further 30 min under argon. The reaction was allowed to reach room temperature and the mixture was left stirring for an additional 48 h. The insoluble residue was removed by suction filtration and the solvent removed under reduced pressure. CH_2Cl_2 was added to the crude oil before washing twice with 1 M HCl, a saturated solution of $NaHCO_3$, and H_2O. The organic layer was then dried over $MgSO_4$, filtered, and the solvent removed under reduced pressure. Purification was achieved by flash silica column chromatography (gradient elution 100:0 → 95:5; CH_2Cl_2/MeOH) followed by precipitation, done by dissolution in a minimum volume of MeOH, and dropping into H_2O. The product was isolated by filtration and dried over P_2O_5, affording **1***(S,S)* as a cream colored solid (1.68 g, 3.43 mmol, 63% yield). m.p. 159 – 161 °C; HRMS (*m/z*) (ES$^+$) Calculated for $C_{31}H_{27}N_3O_3Na$ *m/z* = 512.1950 [M + Na]$^+$. Found *m/z* = 512.1964; ^{1}H NMR (600 MHz, CD_3CN-*d*$_3$) δ_H: 8.55 (1H, d, *J* = 1.3 Hz, NH), 8.25 (2H, d, *J* = 1.4 Hz, naph-H), 7.97 (2H, d, *J* = 1.6 Hz, naph-H), 7.87 (2H, d, *J* = 1.4 Hz, naph-H), 7.70 (2H, d, *J* = 0.7 Hz, py-H), 7.68 (2H, d, *J* = 1.2 Hz, naph-H), 7.58 (2H, m, naph-H), 7.55 (2H, m, naph-H), 7.54 (2H, m, naph-H), 6.07 (2H, m, CH), 1.73 (6H, d, *J* = 1.1 Hz, CH_3); ^{13}C NMR (150 MHz, CD_3CN-*d*$_3$) δ_C: 167.07, 163.71, 152.22,

140.20, 134.81, 131.85, 129.74, 128.75, 127.27, 126.68, 126.38, 124.14, 123.85, 113.10, 46.14, 21.40; IR υ_{max} (cm^{-1}): 3283, 1653, 1600, 1521, 1449, 1355, 1238, 1178, 1136, 997, 889, 857, 799, 775, 691.

4-Hydroxy-*N*,*N'*-bis((*R*)-1-(naphthalen-1-yl)ethyl)pyridine-2,6-dicarboxamide 1*(R,R)*

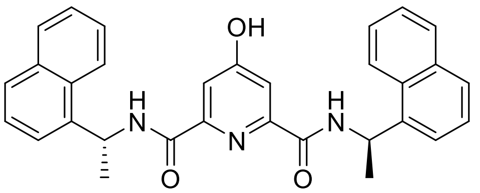

To a stirred solution of (*R*)-(+)-1-(1-naphthyl)-ethyl-amine (1.96 g, 1.84 mL, 11.47 mmol, 2.10 eq.) in anhydrous THF (35 mL), HOBt (1.48 g, 10.92 mmol, 2.00 eq.) and chelidamic acid (1.00 g, 5.46 mmol, 1.00 eq.) were added. The solution was stirred for 30 min at 0 °C before addition of EDCI·HCl (2.15 g, 11.19 mmol, 2.05 eq.) and triethylamine (1.10 g, 1.52 mL, 10.92 mmol, 2.00 eq.), with stirring for a further 30 min under argon. The reaction was allowed to reach room temperature and the mixture was left stirring for an additional 48 h. The insoluble residue was removed by suction filtration and the solvent removed under reduced pressure. CH_2Cl_2 was added to the crude oil before washing twice with 1 M HCl, a saturated solution of $NaHCO_3$, and H_2O. The organic layer was then dried over $MgSO_4$, filtered, and the solvent removed under reduced pressure. Purification was achieved by flash silica column chromatography (gradient elution 100:0 → 95:5; CH_2Cl_2/MeOH) followed by precipitation, done by dissolution in a minimum volume of MeOH, and dropping into H_2O. The product was isolated by filtration and dried over P_2O_5, affording **1***(R,R)* as a cream coloured solid (1.50 g, 3.06 mmol, 56% yield). m.p. 159 – 161 °C; HRMS (*m/z*) (ES$^-$) Calculated for $C_{31}H_{26}N_3O_3$ *m/z* = 488.1974 [M − H]$^-$. Found *m/z* = 488.1978; ^{1}H NMR (600 MHz, CD_3CN-*d*$_3$) δ_H: 8.55 (1H, d, *J* = 1.3 Hz, N<u>H</u>), 8.25 (2H, d, *J* = 1.4 Hz, naph-<u>H</u>), 7.97 (2H, d, *J* = 1.6 Hz, naph-<u>H</u>), 7.87 (2H, d, *J* = 1.4 Hz, naph-<u>H</u>), 7.70 (2H, d, *J* = 0.7 Hz, py-<u>H</u>), 7.68 (2H, d, *J* = 1.2 Hz, naph-<u>H</u>), 7.58 (2H, m, naph-<u>H</u>), 7.55 (2H, m, naph-<u>H</u>), 7.54 (2H, m, naph-<u>H</u>), 6.07 (2H, m, C<u>H</u>), 1.73 (6H, d, *J* = 1.1 Hz, C<u>H</u>$_3$); ^{13}C NMR (150 MHz, CD_3CN-*d*$_3$) δ_C: 167.10, 163.74, 152.23, 140.23, 134.84, 131.90, 129.76, 128.77, 127.29, 126.69, 126.39, 124.15, 123.85, 113.11, 46.15, 21.41; IR υ_{max} (cm^{-1}): 3283, 1653, 1600, 1521, 1449, 1355, 1238, 1178, 1136, 997, 889, 857, 799, 775, 691.

Charactersation of ligands 2-4 (*S,S* and *R,R*)

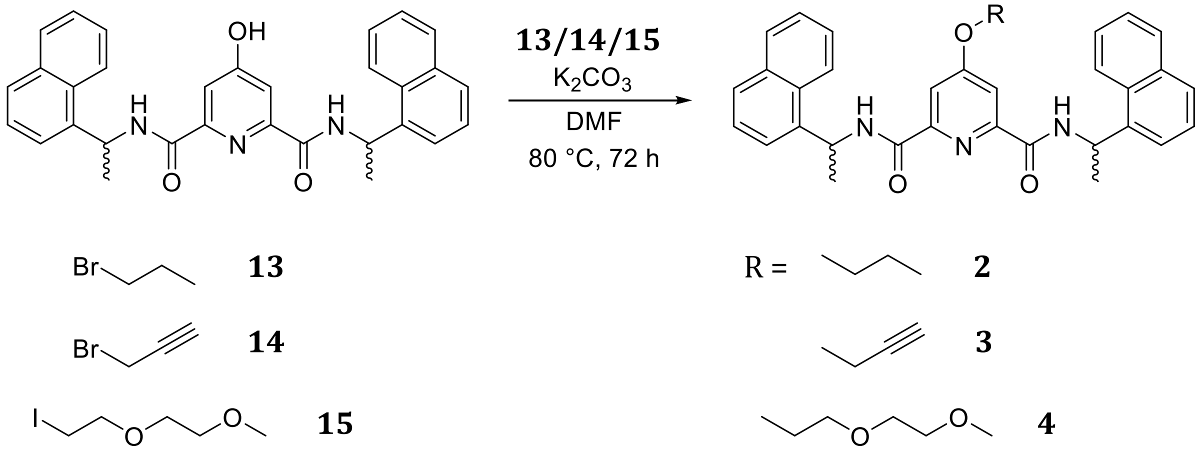

Scheme S2: *Synthesis of ligands* ***2-4*** *from ligand* ***1****(either S,S or R,R) using the alkyl halides* ***13-15*** *(formed as either S,S or R,R ligands from the S,S or R,R version of* ***1****).*

Procedure 1: Nucleophile substitution reactions of 1*(S,S)* **and 1***(R,R)*

Nucleophilic substitution at the 4-hydroxy group of **1***(S,S) or* **1***(R,R)* was accomplished by first stirring **1***(S,S) or* **1***(R,R)* with anhydrous K_2CO_3 in anhydrous DMF for 30 min at 25 °C. The relevant organic halide was then added, along with a catalytic amount of KI, and the solution heated at 80 °C for 72 h. After reaction completion, the solvent was removed under reduced pressure and CH_2Cl_2 was added to the residue. The organic layer was subsequently washed twice with 1% aqueous acetic acid and H_2O, before drying over $MgSO_4$. In each case, removal of the solvent under reduced pressure yielded a crude oil requiring further purification.

4-Propoxy-*N,N'*-bis((*S*)-1-(naphthalen-1-yl)ethyl)pyridine-2,6-dicarboxamide (2(*S,S*))

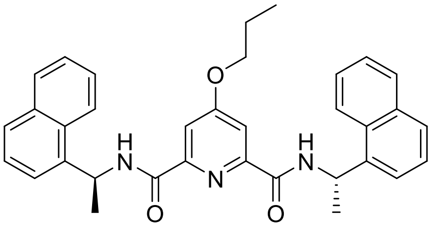

Compound **2**(*S,S*) was synthesized according to **Procedure 1** using 1-bromopropane as the alkylating agent. The quantities of the respective reagents and solvent were as follows; **1**(*S,S*) (0.20 g, 0.41 mmol, 1.00 eq.), K_2CO_3 (0.11 g, 0.82 mmol, 2.00 eq.), 1-bromopropane (**13**, 0.15 g, 0.11 mL, 1.23 mmol, 3.00 eq.), DMF (5 mL). The crude product was recrystallized from EtOH to

give **2**(*S,S*) as a white crystalline solid (0.07 g, 0.13 mmol, 32% yield). m.p. 197 – 199 °C; HRMS (*m/z*) (ES+) Calculated for $C_{34}H_{34}N_3O_3$ *m/z* = 532.2600 [M + H]+. Found *m/z* = 532.2603; ^{1}H NMR (600 MHz, $CDCl_3$) δ_H: 8.15 (2H, m, naph-H), 7.92 (2H, d, *J* = 8.4 Hz, NH), 7.88 (2H, m, naph-H), 7.84 – 7.78 (4H, m, py-H + naph-H), 7.54 – 7.47 (4H, m, naph-H), 7.45 – 7.40 (4H, m, naph-H), 6.01 (2H, m, CH), 4.04 (2H, t, *J* = 6.5 Hz, OCH_2), 1.83 (2H, m, OCH_2CH_2), 1.63 (6H, d, *J* = 6.9 Hz, CH_3), 1.02 (3H, t, *J* = 7.3 Hz, $OCH_2CH_2CH_3$); ^{13}C NMR (150 MHz, $CDCl_3$) δ_C: 168.08, 162.54, 150.64, 138.41, 134.13, 131.11, 129.07, 128.58, 126.75, 126.05, 125.43, 123.43, 122.87, 111.61, 70.59, 45.45, 22.26, 21.18, 10.43; IR υ_{max} (cm^{-1}): 3285, 3050, 2968, 2877, 1659, 1638, 1597, 1509, 1474, 1453, 1354, 1305, 1262, 1236, 1210, 1172, 1120, 1094, 1081, 1033, 996, 949, 910, 882, 855, 813, 800, 776, 753, 733, 697, 620, 604, 587, 565.

4-Propoxy-*N,N'*-bis((*R*)-1-(naphthalen-1-yl)ethyl)pyridine-2,6-dicarboxamide (2(*R,R*))

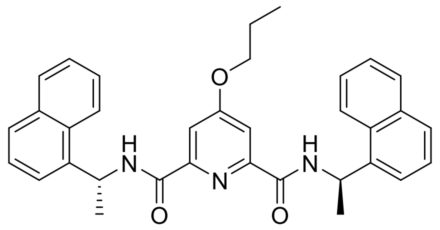

Compound **2**(*R,R*) was synthesized according to **Procedure 1** using 1-bromopropane as the alkylating agent. The quantities of the respective reagents and solvent were as follows; **1**(*R,R*) (0.20 g, 0.41 mmol, 1.00 eq.), K_2CO_3 (0.11 g, 0.82 mmol, 2.00 eq.), 1-bromopropane (**13**, 0.15 g, 0.11 mL, 1.23 mmol, 3.00 eq.), DMF (5 mL). The crude product was recrystallized from EtOH to give **2(*RR*)** as a white crystalline solid (0.08 g, 0.15 mmol, 37% yield). m.p. 197 – 199 °C; HRMS (*m/z*) (ES+) Calculated for $C_{34}H_{34}N_3O_3$ *m/z* = 532.2600 [M + H]+. Found *m/z* = 532.2582; ^{1}H NMR (400 MHz, $CDCl_3$) δ_H: 8.15 (2H, m, naph-H), 7.95 (2H, d, *J* = 8.4 Hz, NH), 7.88 (2H, m, naph-H), 7.84 – 7.78 (4H, m, py-H + naph-H), 7.54 – 7.47 (4H, m, naph-H), 7.45 – 7.39 (4H, m, naph-H), 6.01 (2H, m, CH), 4.04 (2H, t, *J* = 6.5 Hz, OCH_2), 1.83 (2H, m, OCH_2CH_2), 1.63 (6H, d, *J* = 6.9 Hz, CH_3), 1.02 (3H, t, *J* = 7.3 Hz, $OCH_2CH_2CH_3$); ^{13}C NMR (150 MHz, $CDCl_3$) δ_C: 168.14, 162.38, 150.49, 138.35, 134.09, 131.06, 129.05, 128.54, 126.71, 126.02, 125.41, 123.40, 122.87, 111.67, 70.62, 45.46, 22.24, 21.13, 10.41; IR υ_{max} (cm^{-1}): 3282, 3050, 2963, 2877, 1658, 1636, 1597, 1509, 1474, 1453, 1354, 1304, 1261, 1237, 1210,

1173, 1119, 1095, 1081, 1033, 996, 949, 911, 882, 855, 813, 799, 775, 754, 733, 691, 620, 604, 587, 564.

4-(Prop-2-ynyloxy)-*N*,*N'*-bis((*S*)-1-(naphthalen-1-yl)ethyl)pyridine-2,6-dicarboxamide (3(*S,S*))

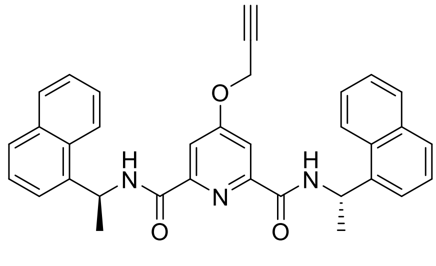

Compound **3**(*S,S*) was synthesized according to **Procedure 1** using propargyl bromide as the alkylating agent. The quantities of the respective reagents and solvent were as follows; **1**(*S,S*) (0.50 g, 1.02 mmol, 1.00 eq.), K_2CO_3 (0.28 g, 2.04 mmol, 2.00 eq.), propargyl bromide solution (**14**, 80% *w/v* in toluene) (0.75 g, 0.56 mL, 5.10 mmol, 5.00 eq.), DMF (20 mL). The crude product was recrystallized from EtOH to give **3**(*S,S*) as a white fibrous solid (0.31 g, 0.59 mmol, 58% yield). m.p. 130 – 133 °C; Calculated for $C_{36}H_{37}N_3O_5{\cdot}0.5H_2O$: C, 76.57; H, 5.57; N, 7.79. Found C, 76.73; H, 5.18; N, 7.70; HRMS (*m/z*) (ES$^+$) Calculated for $C_{34}H_{29}N_3O_3Na$ *m/z* = 550.2107 [M + Na]$^+$. Found *m/z* = 550.2116; ^{1}H NMR (600 MHz, $CDCl_3$) δ_H: 8.15 (2H, m, naph-H), 7.91 – 7.84 (6H, m, NH + py-H + naph-H), 7.81 (2H, m, naph-H), 7.53 – 7.48 (4H, m, naph-H), 7.44 – 7.40 (4H, m, naph-H), 6.00 (2H, m, NCHH), 4.80 (2H, t, *J* = 3.2 Hz, CH_2), 2.58 (1H, t, *J* = 2.3 Hz, alkyne-CH), 1.63 (6H, d, *J* = 6.7 Hz, CH_3); ^{13}C NMR (150 MHz, $CDCl_3$) δ_C: 166.36, 162.22, 150.78, 138.21, 134.00, 130.94, 128.95, 128.46, 126.62, 125.94, 125.27, 123.25, 122.71, 111.69, 56.27, 45.35, 21.02; IR υ_{max} (cm^{-1}): 3273, 2971, 1643, 1597, 1510, 1445, 1372, 1343, 1305, 1261, 1236, 1172, 1132, 1032, 997, 950, 914, 881, 800, 776, 695, 636, 587, 574.

4-(Prop-2-ynyloxy)-*N*,*N'*-bis((*R*)-1-(naphthalen-1-yl)ethyl)pyridine-2,6-dicarboxamide (3(*R,R*))

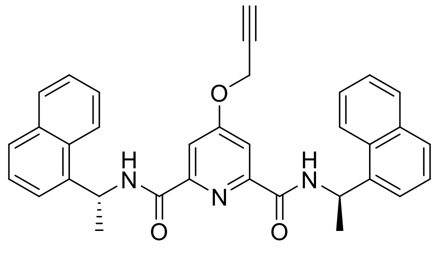

Compound **3**(*R,R*) was synthesized according to **Procedure 1** using propargyl bromide as the alkylating agent. The quantities of the respective reagents and solvent were as follows; **1**(*R,R*) (0.50 g, 1.02 mmol, 1.00 eq.), K_2CO_3 (0.28 g, 2.04 mmol, 2.00 eq.), propargyl

bromide solution (**14**, 80% *w/v* in toluene) (0.75 g, 0.56 mL, 5.10 mmol, 5.00 eq.), DMF (20 mL). The crude product was recrystallized from EtOH to give **3**(*R,R*) as a white fibrous solid (0.36 g, 0.68 mmol, 67% yield). m.p. 130 – 133 °C; Calculated for $C_{34}H_{29}N_3O_3 \cdot 0.2CHCl_3$: C, 74.48; H, 5.34; N, 7.62. Found C, 74.32; H, 5.02; N, 7.38; HRMS (*m/z*) (ES$^+$) Calculated for $C_{34}H_{30}N_3O_3$ *m/z* = 528.2287 [M + H]$^+$. Found *m/z* = 528.2283; ^{1}H NMR (600 MHz, $CDCl_3$) δ_H: 8.15 (2H, m, naph-H), 7.92 – 7.86 (6H, m, NH + py-H + naph-H), 7.82 (2H, m, naph-H), 7.53 – 7.48 (4H, m, naph-H), 7.44 – 7.41 (4H, m, naph-H), 6.01 (2H, m, NHCH), 4.80 (2H, t, *J* = 3.2 Hz, CH_2), 2.58 (1H, t, *J* = 2.3 Hz, alkyne-CH), 1.63 (6H, d, *J* = 6.7 Hz, CH_3); ^{13}C NMR (150 MHz, $CDCl_3$) δ_C: 166.56, 162.29, 150.89, 138.34, 134.14, 131.09, 129.09, 128.61, 126.77, 126.08, 125.43, 123.39, 122.86, 111.89, 56.45, 45.52, 21.17; IR υ_{max} (cm^{-1}): 3281, 2979, 1645, 1598, 1511, 1446, 1373, 1343, 1305, 1261, 1236, 1173, 1132, 1041, 997, 949, 914, 882, 801, 776, 696, 639, 587, 575.

4-(2-(2-Methoxy-ethoxy)ethoxy)-*N,N'*-bis((*S*)-1-(naphthalen-1-yl)ethyl)pyridine-2,6-dicarboxamide (4(*S,S*))

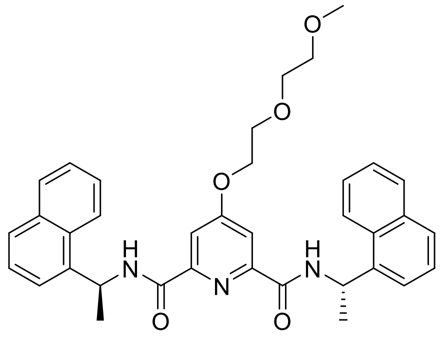

Compound **4**(*S,S*) was synthesized according to **Procedure 1** using 1-iodo-2-(2-methoxy-ethoxy)ethane as the alkylating agent. The quantities of the respective reagents and solvent were as follows; **1** (0.50 g, 1.02 mmol, 1.00 eq.), K_2CO_3 (0.28 g, 2.04 mmol, 2.00 eq.), **15** (0.35 g, 1.53 mmol, 1.50 eq.), DMF (20 mL). The crude product was recrystallized from EtOH to give **4**(*S,S*) as a white crystalline solid (0.31 g, 0.52 mmol, 51% yield). m.p. 155 – 157 °C; Calculated for $C_{36}H_{37}N_3O_5 \cdot 0.25H_2O$: C, 72.52; H, 6.34; N, 7.05. Found C, 72.50; H, 6.06; N, 6.89; HRMS (*m/z*) (ES$^+$) Calculated for $C_{36}H_{37}N_3O_5Na$ *m/z* = 614.2631 [M + Na]$^+$. Found *m/z* = 614.2631; ^{1}H NMR (600 MHz, $CDCl_3$) δ_H: 8.15 (2H, m, naph-H), 7.89 (2H, m, naph-H), 7.85 – 7.79 (6H, m, NH + py-H + naph-H), 7.53 – 7.49 (4H, m, naph-H), 7.45 – 7.41 (4H, m, naph-H), 6.00 (2H, m, CH), 4.27 (2H, m, CH_2), 3.88 (2H, m, CH_2), 3.71 (2H, m, CH_2), 3.57 (2H, m, CH_2), 3.38 (3H, s, OCH_3), 1.64 (6H, d, *J* = 6.7 Hz, CH_3); ^{13}C NMR (150 MHz, $CDCl_3$) δ_C: 167.48, 162.28, 150.61, 138.15, 133.93, 130.89, 128.92, 128.43, 126.57, 125.88, 125.20, 123.23, 122.65, 111.36, 71.92,

70.84, 69.07, 68.29, 59.07, 45.23, 20.90; IR υ_{max} (cm^{-1}): 3287, 3049, 2973, 2928, 2872, 1655, 1636, 1598, 1568, 1512, 1443, 1398, 1371, 1349, 1335, 1307, 1279, 1236, 1209, 1172, 1128, 1117, 1105, 1081, 1061, 1028, 996, 963, 950, 889, 862, 853, 803, 775, 753, 736, 725, 696, 687.

4-(2-(2-Methoxy-ethoxy)ethoxy)-*N,N'*-bis((*R*)-1-(naphthalen-1-yl)ethyl)pyridine-2,6-dicarboxamide (4(*R,R*))

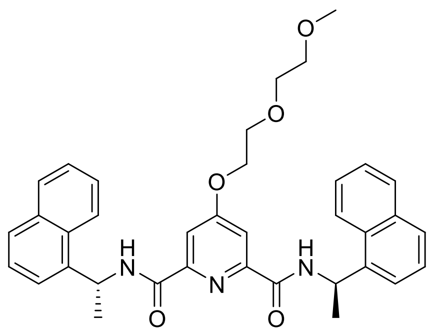

Compound **4**(*R,R*) was synthesized according to **Procedure 1** using 1-iodo-2-(2-methoxy-ethoxy)ethane as the alkylating agent. The quantities of the respective reagents and solvent were as follows; **1**(*R,R*) (0.50 g, 1.02 mmol, 1.00 eq.), K_2CO_3 (0.28 g, 2.04 mmol, 2.00 eq.), **15** (0.35 g, 1.53 mmol, 1.50 eq.), DMF (20 mL). The crude product was recrystallized from EtOH to give **4**(*R,R*) as a white crystalline solid (0.29 g, 0.49 mmol, 48% yield). m.p. 155 – 157 °C; Calculated for $C_{36}H_{37}N_3O_5\cdot 0.25H_2O$: C, 72.52; H, 6.34; N, 7.05. Found C, 72.52; H, 6.10; N, 6.94; HRMS (*m/z*) (ES$^+$) Calculated for $C_{36}H_{38}N_3O_5$ *m/z* = 592.2811 [M + H]$^+$. Found *m/z* = 592.2828; ^{1}H NMR (600 MHz, $CDCl_3$) δ_H: 8.15 (2H, m, naph-H), 7.89 (2H, m, naph-H), 7.86 – 7.79 (6H, m, NH + py-H + naph-H), 7.53 – 7.49 (4H, m, naph-H), 7.45 – 7.41 (4H, m, naph-H), 6.01 (2H, m, CH), 4.27 (2H, m, CH_2), 3.88 (2H, m, CH_2), 3.71 (2H, m, CH_2), 3.57 (2H, m, CH_2), 3.39 (3H, s, OCH_3), 1.64 (6H, d, *J* = 6.7 Hz, CH_3); ^{13}C NMR (150 MHz, $CDCl_3$) δ_C: 167.64, 162.21, 150.50, 138.18, 133.95, 130.91, 128.90, 128.41, 126.58, 125.90, 125.26, 123.24, 122.68, 111.48, 71.88, 70.87, 69.07, 68.32, 59.08, 45.32, 21.00; IR υ_{max} (cm^{-1}): 3287, 2972, 2927, 2872, 1655, 1636, 1599, 1568, 1513, 1444, 1397, 1371, 1349, 1307, 1236, 1172, 1118, 1105, 1081, 1061, 1028, 996, 889, 862, 803, 775, 753, 725, 688.

Charactersation of Eu(III) complexes of ligands 1-4 (*S,S* and *R,R*)

Synthesis of Eu(III)complexes of 5S and 6R

Compound **1** (*S,S* or *R,R*) (0.083 mmol) and $Eu(CF_3SO_3)_3$ (0.027 mmol) were added to a 10 mL RBF that contained freshly dried MeOH (5 mL). The solution was freeze-thawed three times, placed under an argon atmosphere, and left

stirring at reflux for 24 hrs. The resulting solution was cooled to room temperature and then precipitated slowly in diethyl ether (100 mL). The solid was filtered off, recrystallised from MeOH, and dried under vacuum.

Complex 5S

m.p. 230 - 232°C; Calculated for $C_{96}H_{81}EuF_9N_9O_{18}S_3$ $(CH_3CH_2)_2O$: C, 55.44; H, 4.51; N, 6.06%. Found $C_{96}H_{81}EuF_9N_9O_{18}S_3$ $(CH_3CH_2)_2O$: C, 56.76; H, 4.30; N, 6.35%; Calculated for $C_{31}H_{27}O_3N_3Eu$: $(M/2)^+$ m/z = 1620.5448; Found 1620.5370; δ_H (400 MHz, CD_3OD, 298 K) 10.30, 10.28, 8.29, 7.43, 7.18, 7.16, 6.56, 6.55, 5.99, 4.92, 3.36, 2.97, 2.31, 1.29, 0.11; δ_H (600 MHz, CD_3CN, 298 K) 10.09, 9.85, 8.27, 7.46, 7.22, 6.63, 6.07, 5.11, 3.24, 2.30. IR υ_{max} (cm^{-1}) 330.05, 2975.28, 1616.80, 1555.10, 1450.91, 1373.92, 1239.72, 1165.27, 1023, 961.47, 878.79, 798.51, 774.99.

Complex 6R

m.p. 230 - 232°C; Calculated for $C_{96}H_{81}EuF_9N_9O_{18}S_3$ $(CH_3CH_2)_2O$: C, 55.44; H, 4.51; N, 6.06%. Found $C_{96}H_{81}EuF_9N_9O_{18}S_3$ $(CH_3CH_2)_2O$: C, 53.48; H, 4.16; N, 5.69%; Calculated for $C_{31}H_{27}O_3N_3Eu$: $(M/2)^+$ m/z = 1620.5448; Found 1620.5370; δ_H (400 MHz, CD_3OD, 298 K) 10.30, 10.28, 8.29, 7.43, 7.18, 7.16, 6.56, 6.55, 5.99, 4.92, 3.36, 2.97, 2.31, 1.29, 0.11; δ_H (600 MHz, CD_3CN, 298 K) 10.09, 9.85, 8.27, 7.46, 7.22, 6.63, 6.07, 5.11, 3.24, 2.30. IR υ_{max} (cm^{-1}) 330.05, 2975.28, 1616.80, 1555.10, 1450.91, 1373.92, 1239.72, 1165.27, 1023, 961.47, 878.79, 798.51, 774.99.

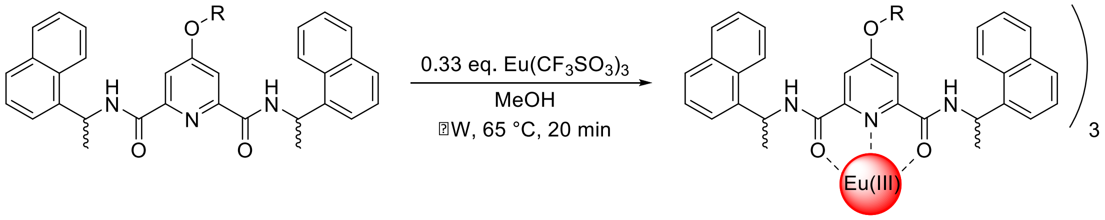

***Scheme S3:** General synthesis of Eu(III) complexes **7-12** using **2-4** (as either **S,S** or **R,R** based ligands) under microwave conditions.*

Procedure 2 Synthesis of Eu(III) complexes **7S/8R, 9S/10R** and **11S/12R**

Complexes **7S/8R, 9S/10R** and **11S/12R** were prepared by refluxing, under microwave irradiation, the pertinent ligand with 0.33 eq. of $Eu(CF_3SO_3)_3$ in HPLC grade solvent (MeOH or CH_3CN) for 20 min. The resulting clear solution was subjected to slow vapor diffusion of diethyl ether, precipitating the desired complex solid. The product was then isolated by filtration and dried under vacuum. Owing to the paramagnetic nature of the Eu(III) ion, ^1H NMR spectra of the complexes consisted of broad signals suggesting however the formation of highly symmetrical complexes. These paramagnetic properties also prevent ^{13}C NMR spectra from being recorded.

Complex 7S

Complex **7S** was synthesized according to **Procedure 2** using ligand **2**(*S,S*) (0.018 g, 0.033 mmol, 1.00 eq.) and $Eu(CF_3SO_3)_3$ (0.007 g, 0.011 mmol, 0.33 eq.) in MeOH (5 mL). A white crystalline solid was obtained (0.019 g, 0.009 mmol, 82% yield). m.p. decomposed above 230 °C; Calculated for $C_{102}H_{99}N_9O_9Eu$ + $3CF_3SO_3$ + $0.75CHCl_3$: C, 55.62; H, 4.40; N, 5.52. Found C, 55.69; H, 3.93; N, 5.10; HRMS (*m/z*) (MALDI$^+$) Calculated for $C_{102}H_{97}N_9O_9Eu$ *m/z* = 1744.6640 [M − $3CF_3SO_3$ − 2H]$^+$. Found *m/z* = 1744.6824; ^1H NMR (400 MHz, CD_3OD-d_4) δ_H: 10.27, 10.18, 8.27, 7.42, 7.13, 6.58, 6.05, 3.03, 2.33, 1.66, 1.00; IR υ_{max} (cm^{-1}): 3277, 3098, 2972, 1618, 1596, 1555, 1448, 1361, 1291, 1237, 1164, 1032, 964, 913, 856, 796, 774, 731, 636, 573.

Complex 8R

Complex **8R** was synthesised according to **Procedure 2** using ligand **2**(*R,R*) (0.018 g, 0.033 mmol, 1.00 eq.) and $Eu(CF_3SO_3)_3$ (0.007 g, 0.011 mmol, 0.33 eq.) in MeOH (5 mL). A white crystalline solid was obtained (0.015 g, 0.007 mmol, 64% yield). m.p. decomposed above 220 °C; Calculated for $C_{102}H_{99}N_9O_9Eu$ + $3CF_3SO_3$: C, 57.48; H, 4.55; N, 5.75. Found C, 57.23; H, 4.34; N, 5.48; HRMS (*m/z*) (MALDI$^+$) Calculated for $C_{102}H_{97}N_9O_9Eu$ *m/z* = 1744.6640 [M − $3CF_3SO_3$ − 2H]$^+$. Found *m/z* = 1744.6713; ^1H NMR (400 MHz, CD_3OD-d_4) δ_H: 10.38, 8.31, 7.42, 7.11, 6.54, 6.00, 2.98, 2.35, 1.64, 0.99; IR υ_{max} (cm^{-1}): 3280, 3103, 2976, 1618,

1596, 1556, 1449, 1361, 1291, 1237, 1164, 1032, 964, 918, 856, 796, 774, 731, 636, 573.

Complex 9S

Complex **9S** was synthesized according to **Procedure 2** using ligand **3**(*S*,*S*) (0.030 g, 0.057 mmol, 1.00 eq.) and $Eu(CF_3SO_3)_3$ (0.011 g, 0.019 mmol, 0.33 eq.) in MeOH (5 mL). A white crystalline solid was obtained (0.031 g, 0.014 mmol, 74% yield). m.p. decomposed above 220 °C; Calculated for $C_{102}H_{87}N_9O_9Eu$ + $3CF_3SO_3$ + $1.5CH_2Cl_2$: C, 55.39; H, 3.93; N, 5.46. Found C, 55.68; H, 3.66; N, 5.24; HRMS (*m/z*) (MALDI$^+$) Calculated for $C_{102}H_{85}N_9O_9Eu$ *m/z* = 1732.5701 [M – $3CF_3SO_3$ – 2H]$^+$. Found *m/z* = 1732.5735; ^1H NMR (400 MHz, CD_3OD-d_4) δ_H: 10.31, 8.29, 7.45, 7.22, 6.62, 6.04, 4.15, 3.80, 3.50, 2.35; IR υ_{max} (cm^{-1}): 3273, 3100, 2978, 1747, 1618, 1597, 1556, 1447, 1369, 1274, 1234, 1152, 1047, 1030, 946, 921, 877, 798, 776, 749, 636, 574.

Complex 10R

Complex **10R** was synthesized according to **Procedure 2** using ligand **3**(*R*,*R*) (0.030 g, 0.057 mmol, 1.00 eq.) and $Eu(CF_3SO_3)_3$ (0.011 g, 0.019 mmol, 0.33 eq.) in MeOH (5 mL). A white crystalline solid was obtained (0.028 g, 0.013 mmol, 68% yield). m.p. decomposed above 230 °C; Calculated for $C_{102}H_{87}N_9O_9Eu$ + $3CF_3SO_3$ + $1.5CH_2Cl_2$: C, 55.39; H, 3.93; N, 5.46. Found C, 55.37; H, 3.63; N, 5.19; HRMS (*m/z*) (MALDI$^+$) Calculated for $C_{102}H_{85}N_9O_9Eu$ *m/z* = 1732.5701 [M – $3CF_3SO_3$ – 2H]$^+$. Found *m/z* = 1732.5709; ^1H NMR (400 MHz, CD_3OD-d_4) δ_H: 10.29, 8.29, 7.47, 7.24, 6.65, 6.08, 4.16, 3.82, 3.53, 2.34; IR υ_{max} (cm^{-1}): 3272, 3098, 2979, 1746, 1619, 1597, 1556, 1447, 1368, 1272, 1234, 1152, 1046, 1030, 946, 920, 876, 801, 777, 749, 637, 574.

Complex 11S

Complex **11S** was synthesized according to **Procedure 2** using ligand **4**(*S*,*S*) (0.028 g, 0.047 mmol, 1.00 eq.) and $Eu(CF_3SO_3)_3$ (0.010 g, 0.016 mmol, 0.33 eq.) in MeOH (5 mL). A white crystalline solid was obtained (0.026 g, 0.011 mmol, 69% yield). m.p. decomposed above 240 °C; Calculated for $C_{108}H_{111}N_9O_{15}Eu$ +

$3CF_3SO_3 + 5H_2O$: C, 54.10; H, 4.95; N, 5.12. Found C, 53.89; H, 4.33; N, 4.69; HRMS (*m/z*) (MALDI$^+$) Calculated for $C_{108}H_{109}N_9O_{15}Eu$ *m/z* = 1924.7277 [M – $3CF_3SO_3$ – 2H]$^+$. Found *m/z* = 1924.7225; ^{1}H NMR (400 MHz, CD_3OD-d_4) δ_H: 10.26, 10.11, 8.26, 7.41, 7.27, 6.59, 5.99, 3.58, 3.51, 3.41, 3.24, 2.25; IR υ_{max} (cm^{-1}): 3284, 2971, 2935, 1739, 1619, 1597, 1557, 1448, 1354, 1287, 1237, 1136, 1060, 1032, 1004, 963, 923, 881, 857, 797, 775, 759, 749, 716.

Complex 12R

Complex **12R** was synthesized according to **Procedure 2** using ligand **4**(*R*,*R*) (0.028 g, 0.047 mmol, 1.00 eq.) and $Eu(CF_3SO_3)_3$ (0.010 g, 0.016 mmol, 0.33 eq.) in MeOH (5 mL). A white crystalline solid was obtained (0.028 g, 0.012 mmol, 75% yield). m.p. decomposed above 240 °C; Calculated for $C_{108}H_{111}N_9O_{15}Eu$ + $3CF_3SO_3 + 3H_2O$: C, 54.90; H, 5.19; N, 5.12. Found C, 54.86; H, 4.45; N, 4.98; HRMS (*m/z*) (MALDI$^+$) Calculated for $C_{108}H_{109}N_9O_{15}Eu$ *m/z* = 1924.7277 [M – $3CF_3SO_3$ – 2H]$^+$. Found *m/z* = 1924.7211; ^{1}H NMR (400 MHz, CD_3OD-d_4) δ_H: 10.29, 10.13, 8.28, 7.43, 7.29, 6.61, 6.00, 3.60, 3.53, 3.42, 3.26, 2.27; IR υ_{max} (cm^{-1}): 3283, 2971, 2936, 1739, 1619, 1597, 1556, 1447, 1353, 1288, 1237, 1135, 1059, 1032, 1004, 962, 923, 882, 857, 797, 775, 759, 749, 716.

Supporting Figures and Tables

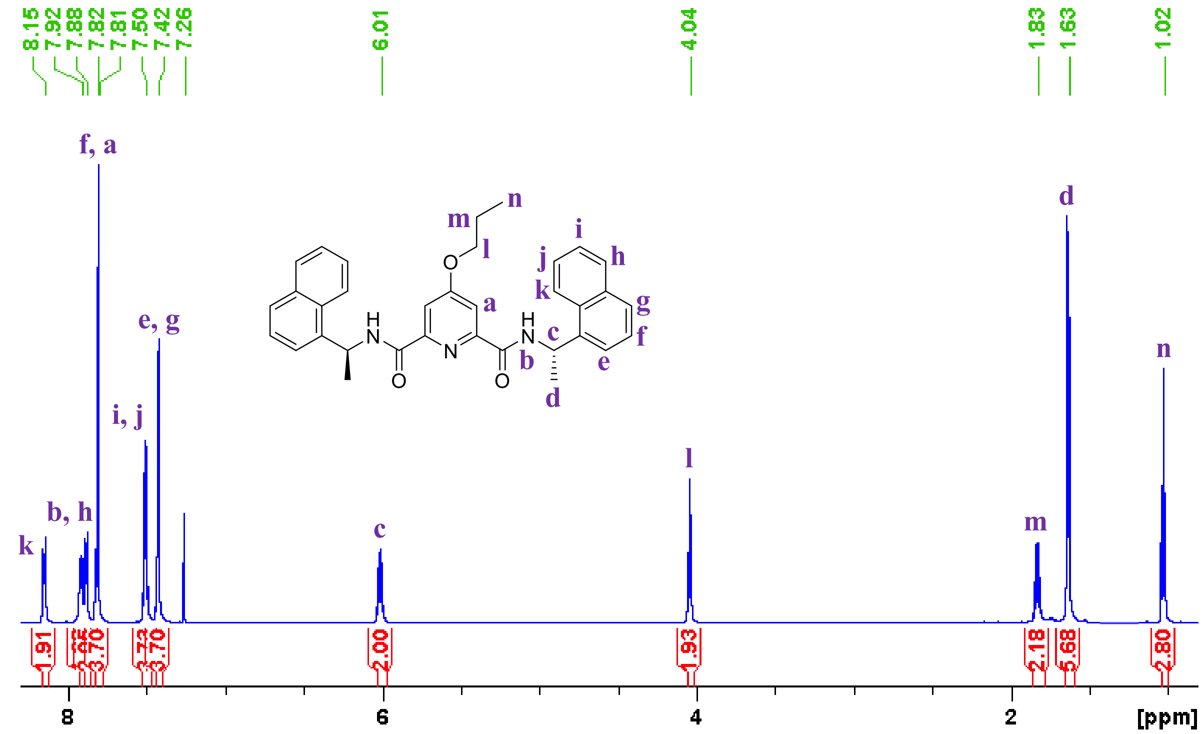

Figure S1: *^{1}H NMR (600 MHz, $CDCl_3$) spectrum of ligand **2**(S,S).*

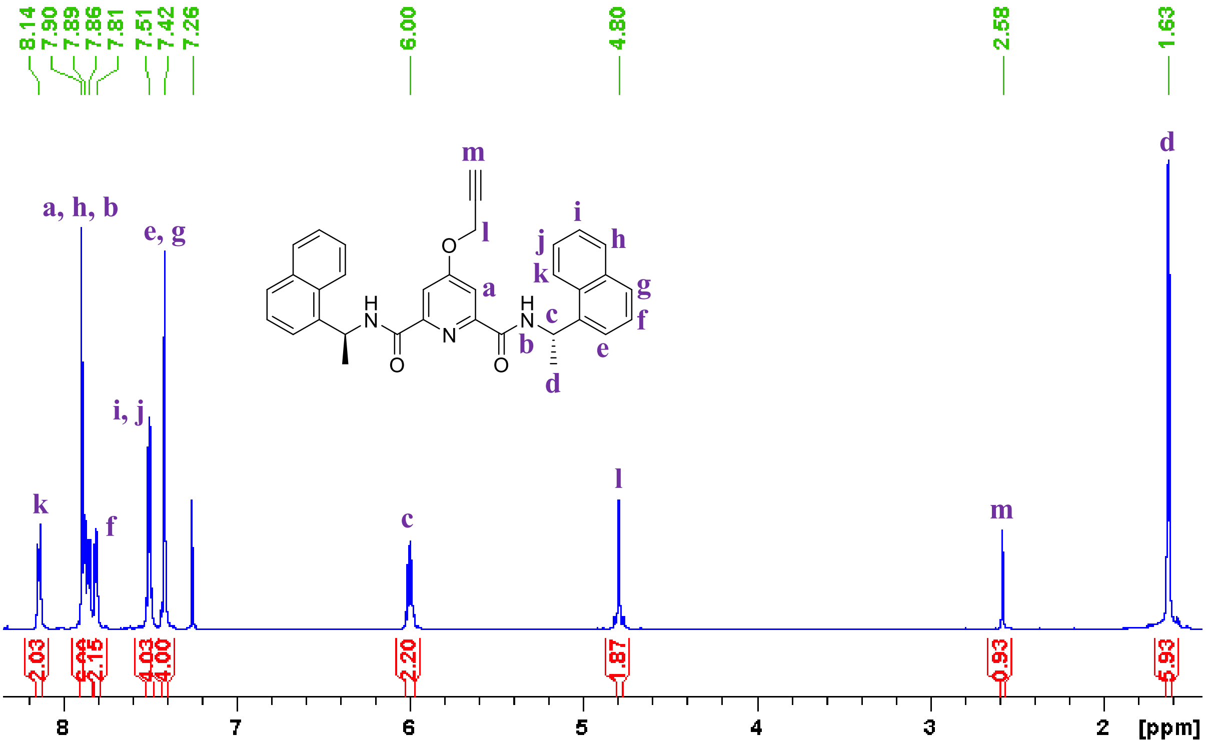

Figure S2: *^{1}H NMR (600 MHz, $CDCl_3$) spectrum of ligand **3**(S,S).*

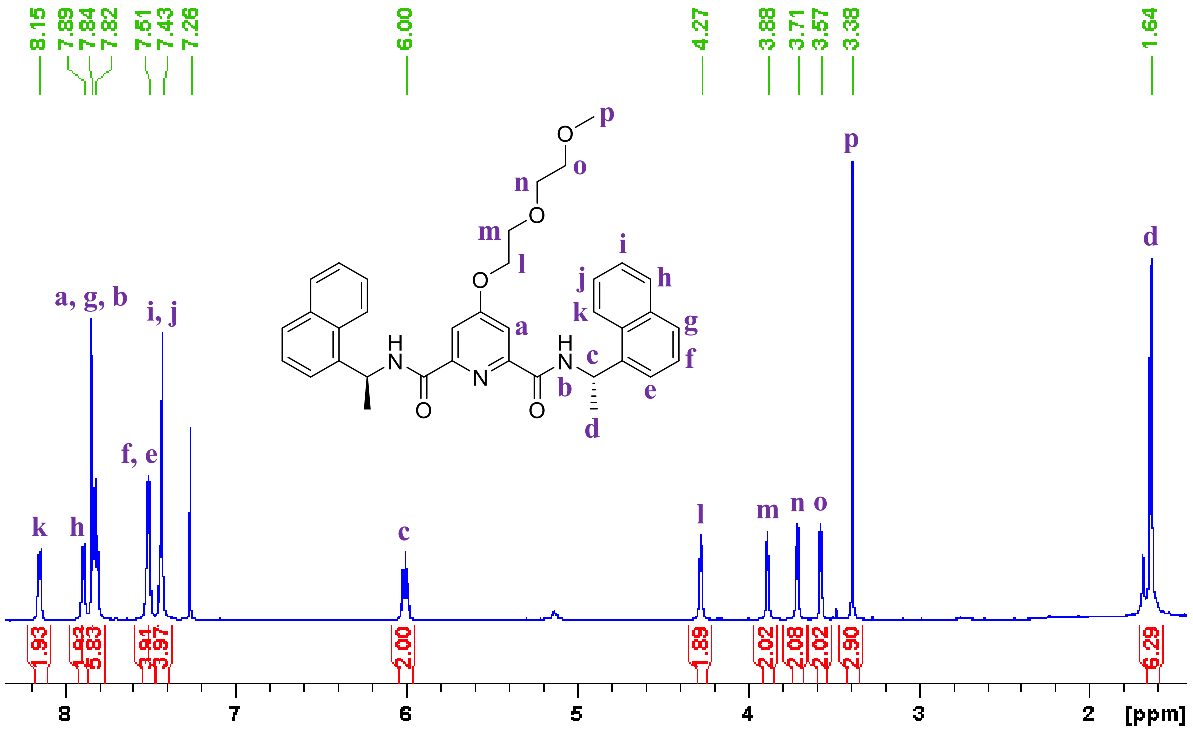

Figure S3: *^{1}H NMR (600 MHz, $CDCl_3$) spectrum of ligand **4**(S,S).*

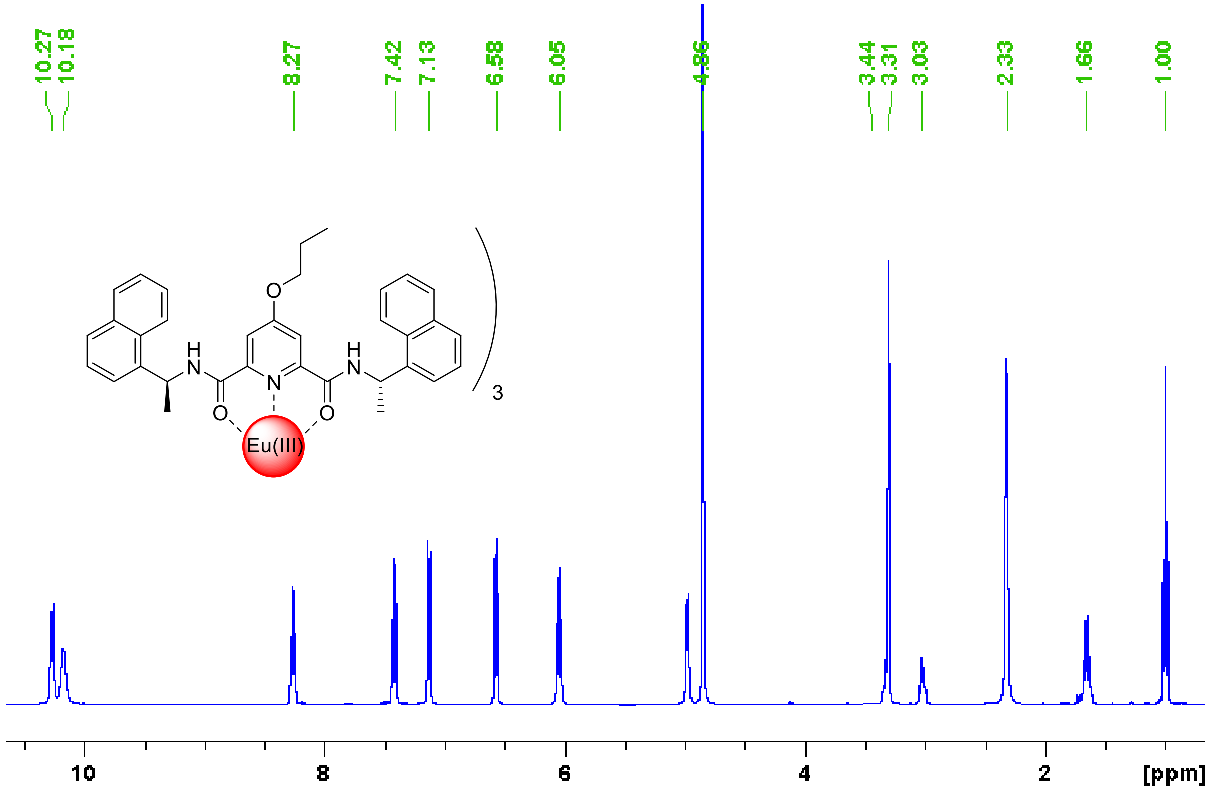

Figure S4: *^{1}H NMR (400 MHz, CD_3OD-d_4) spectrum of complex **7S**.*

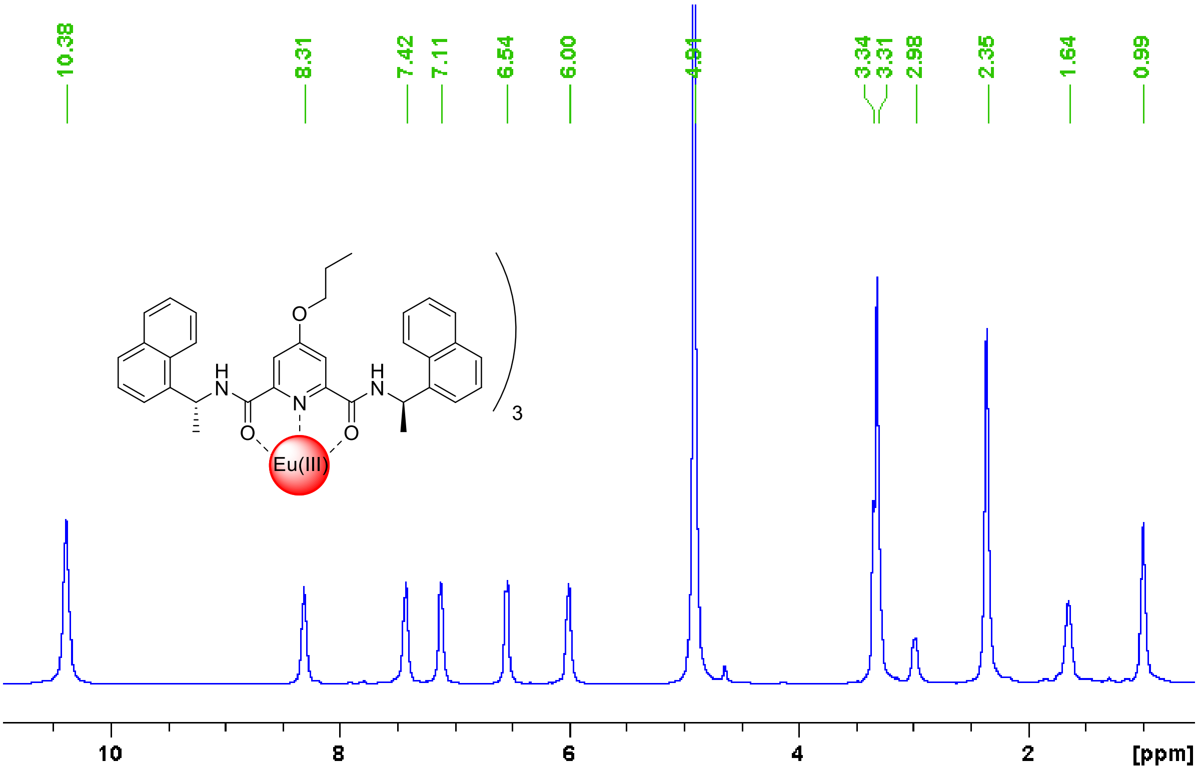

Figure S5: *^{1}H NMR (400 MHz, CD_3OD-d_4) spectrum of complex* ***8R***.

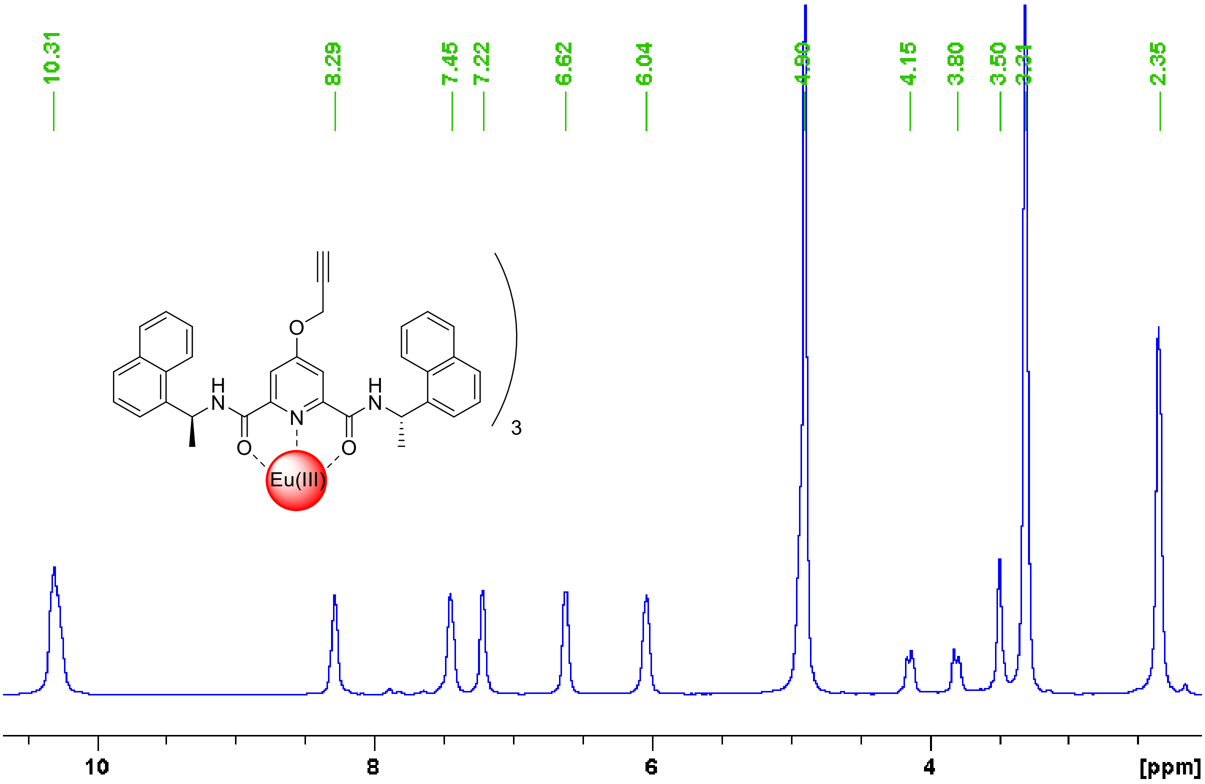

Figure S6: *^{1}H NMR (400 MHz, CD_3OD-d_4) spectrum of complex* ***9S***.

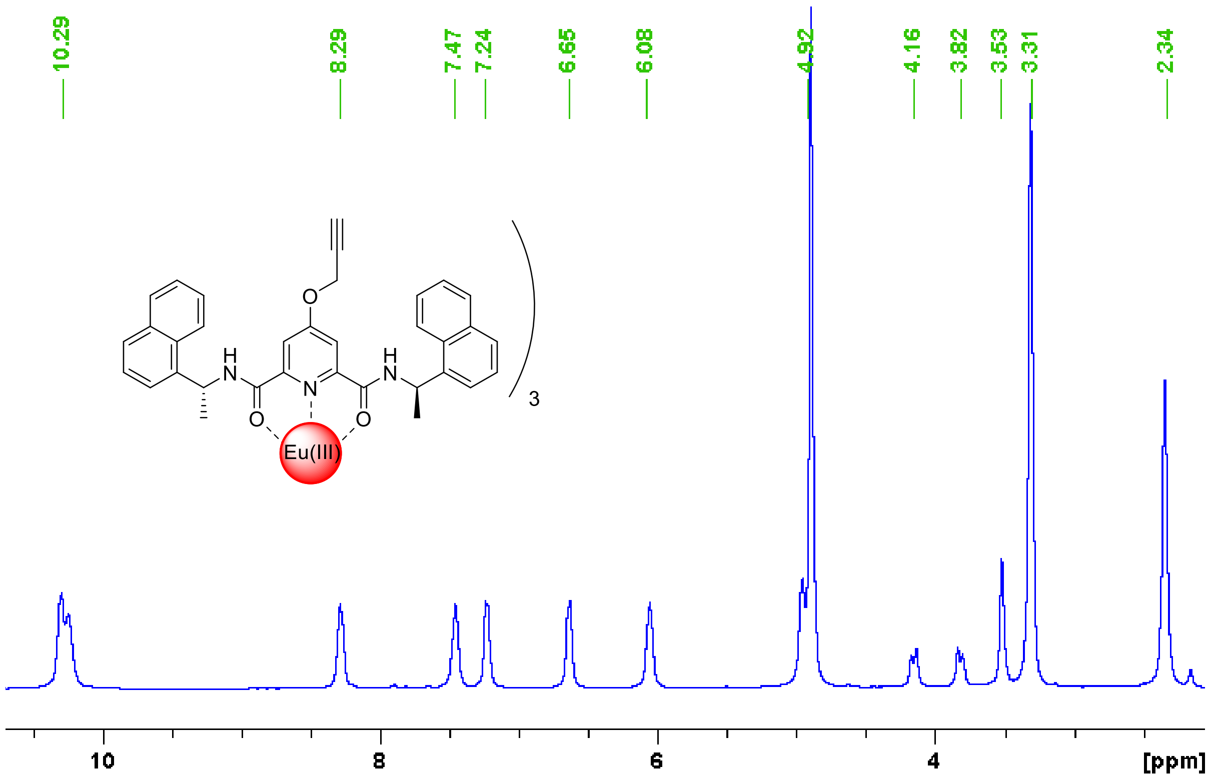

Figure S7: *¹H NMR (400 MHz, CD_3OD-d_4) spectrum of complex* ***10R****.*

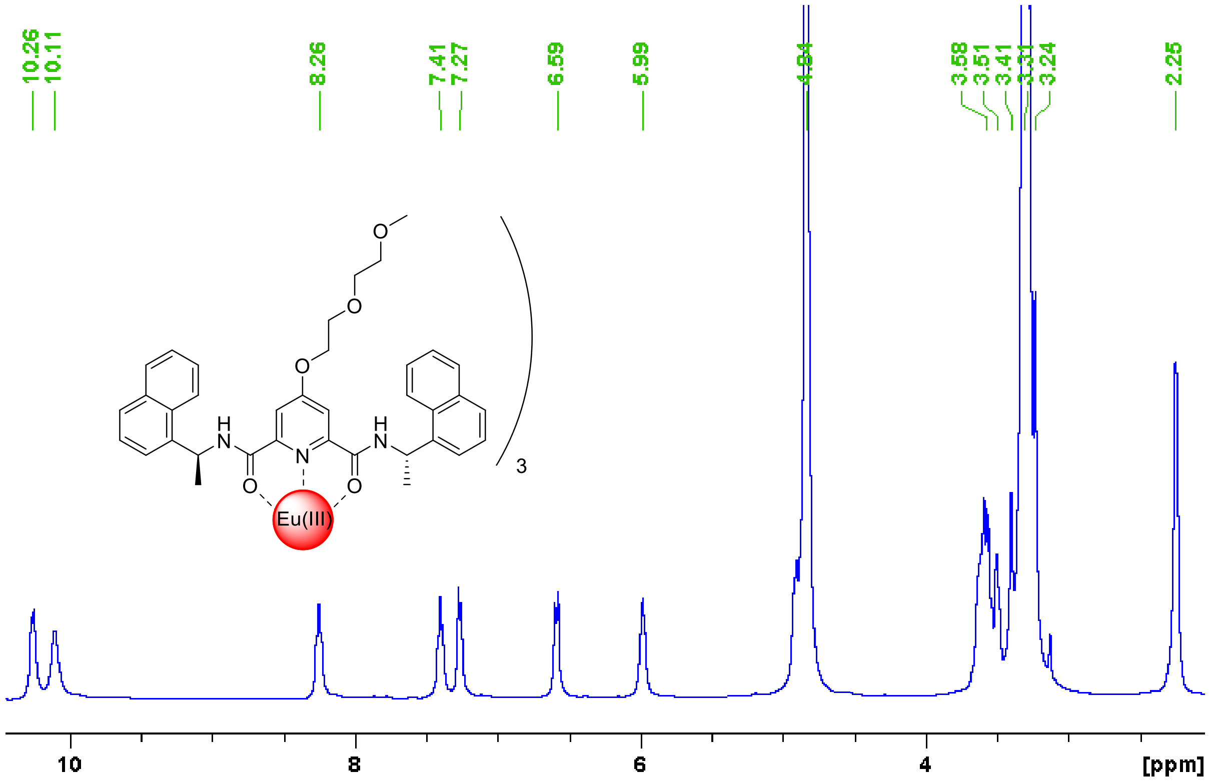

Figure S8: *¹H NMR (400 MHz, CD_3OD-d_4) spectrum of complex* ***11S****.*

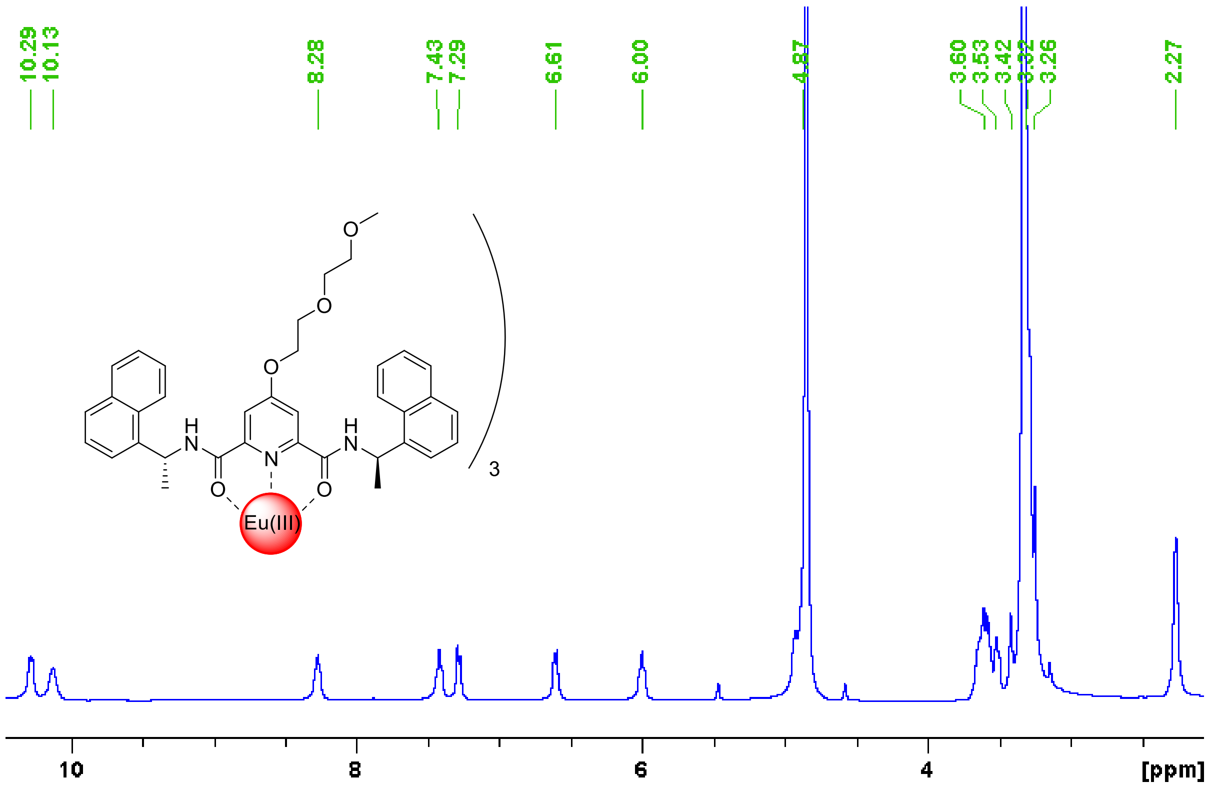

Figure S9: *^{1}H NMR (400 MHz, CD_3OD-d_4) spectrum of complex **12R**.*

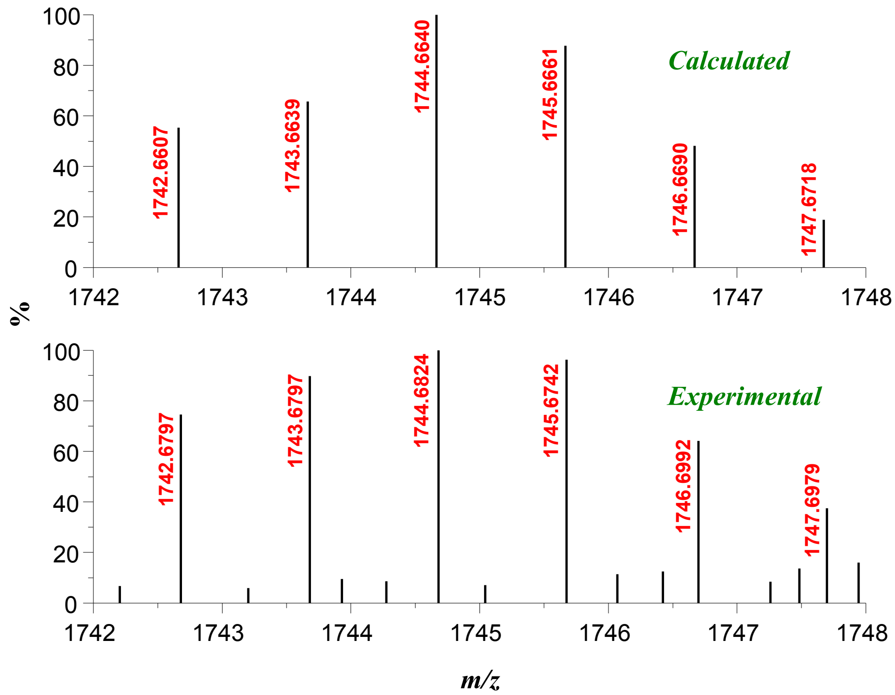

Figure S10: *The calculated and experimental isotopic distribution patterns (MALDI$^+$) for complex **7S** showing the 1:3 (M:L) stoichiometric pattern for a molecular species of formula $[M - 3CF_3SO_3 - 2H]^+$ (spectra are normalised – dominant peak set to 100%).*

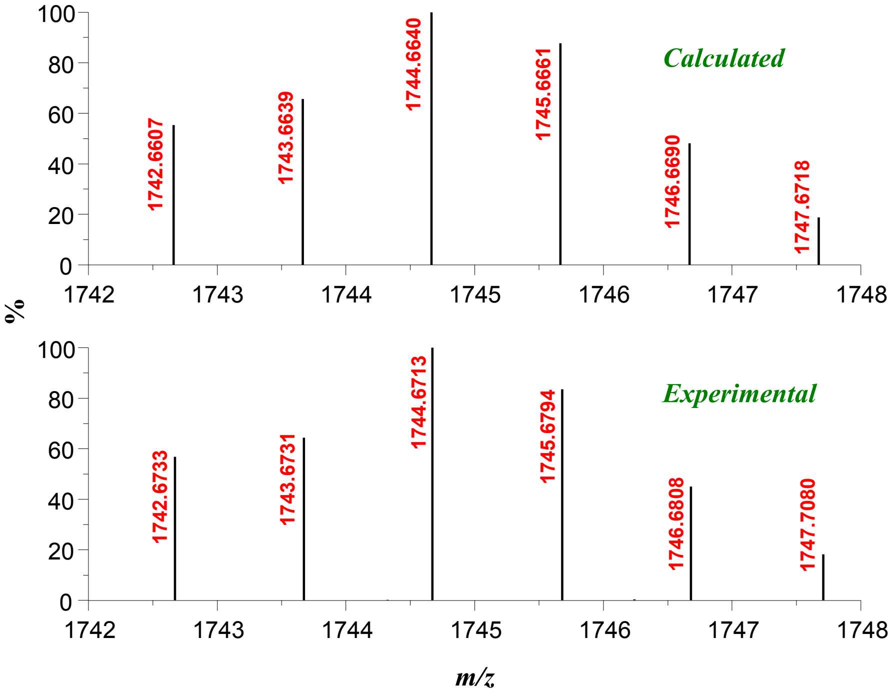

Figure S11: *The calculated and experimental isotopic distribution patterns (MALDI+) for complex* ***8R,*** *showing the 1:3 (M:L) stoichiometric pattern for a molecular species of formula [M – 3CF$_3$SO$_3$ – 2H]$^+$ (spectra are normalised – dominant peak set to 100%).*

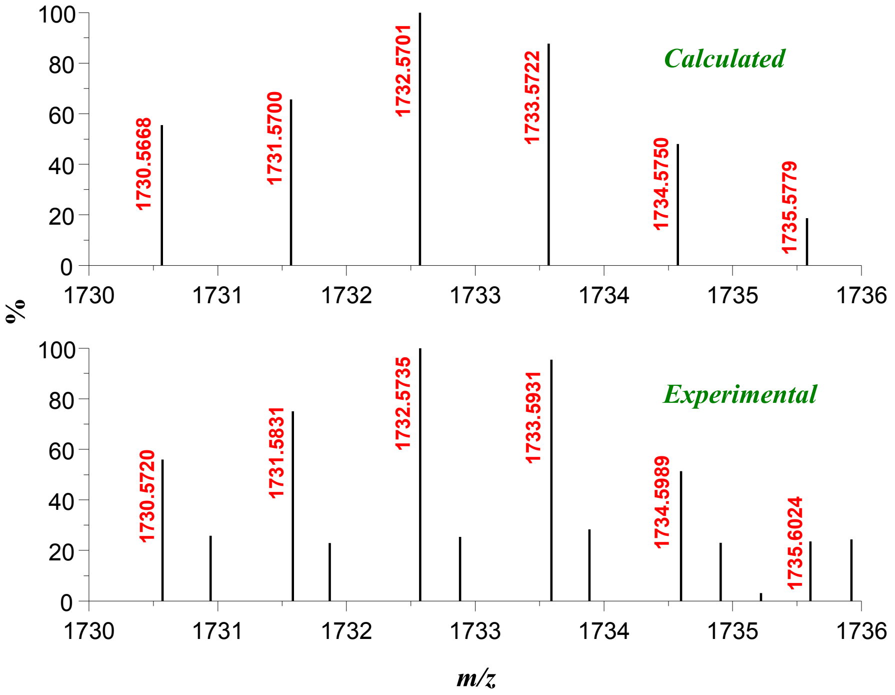

Figure S12: *The calculated and experimental isotopic distribution patterns (MALDI+) for complex* ***9S*** *showing the 1:3 (M:L) stoichiometric pattern for a molecular species of formula [M – 3CF$_3$SO$_3$ – 2H]$^+$ (spectra are normalised – dominant peak set to 100%).*

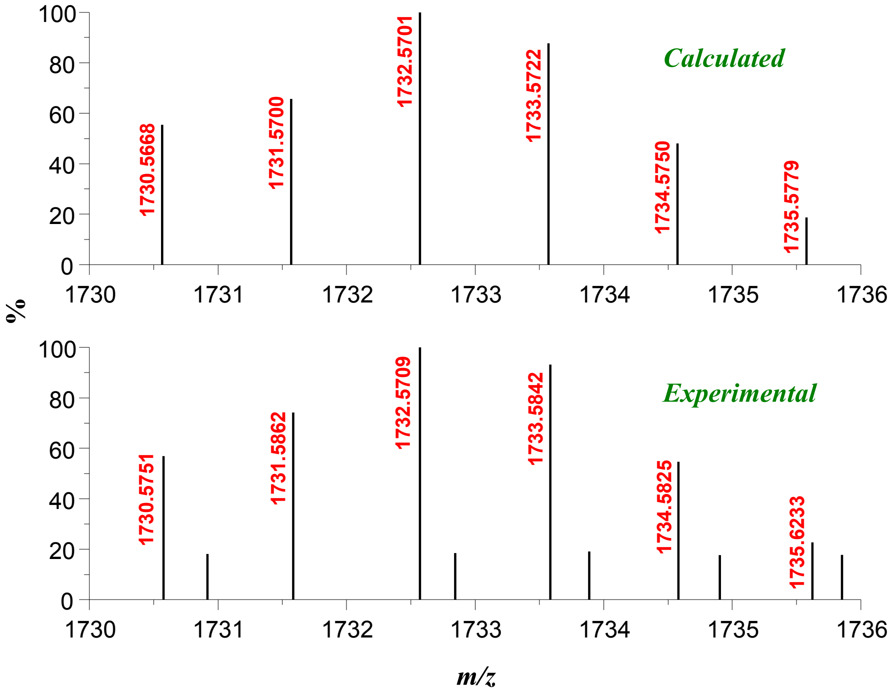

Figure S13: *The calculated and experimental isotopic distribution patterns (MALDI⁺) for complex* ***10R*** *showing the 1:3 (M:L) stoichiometric pattern for a molecular species of formula [M – 3CF_3SO_3 – 2H]$^+$ (spectra are normalised – dominant peak set to 100%).*

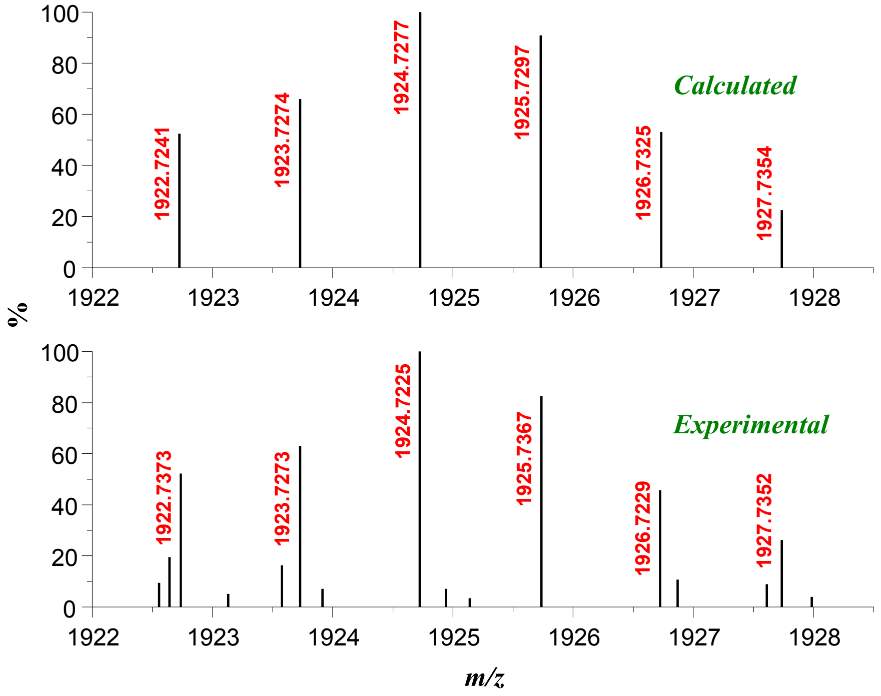

Figure S14: *The calculated and experimental isotopic distribution patterns (MALDI⁺) for complex* ***11S*** *showing the 1:3 (M:L) stoichiometric pattern for a molecular species of formula [M – 3CF_3SO_3 – 2H]$^+$ (spectra are normalised – dominant peak set to 100%).*

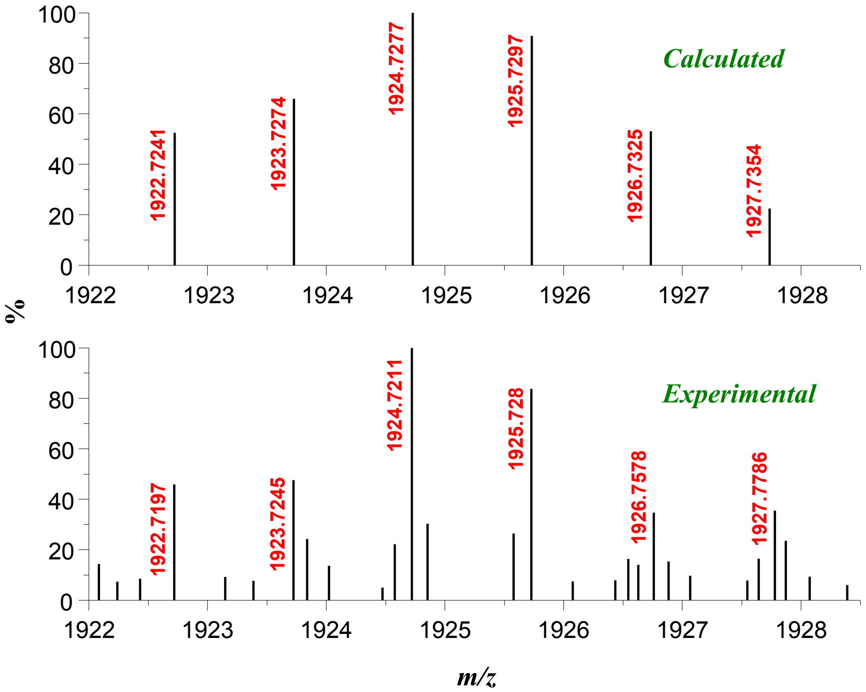

Figure S15: *The calculated and experimental isotopic distribution patterns (MALDI$^+$) for complex* ***12R*** *showing the 1:3 (M:L) stoichiometric pattern for a molecular species of formula [M – 3CF_3SO_3 – 2H]$^+$ (spectra are normalised – dominant peak set to 100%).*

Table S1: *Summary of the experimentally found m/z peaks for* ***7S/8R***, ***9S/10R***, ***11S/12R***, *and their corresponding values calculated for a molecular species of formula [M – 3CF_3SO_3 – 2H]$^+$.*

Complex	*m/z* (experimental)	*m/z* (calculated)
**7S**	1744.6824	1744.6640
**8R**	1744.6713	1744.6640
**9S**	1732.5735	1732.5701
**10R**	1732.5709	1732.5701
**11S**	1924.7225	1924.7277
**12R**	1924.7211	1924.7277

Table S2: *Summary of the IR stretching frequencies (cm^{-1}) of the amide carbonyl groups of the free ligands* ***2-4*** *and their corresponding complexes* ***Eu.(7-12)$_3$***.

Free Ligand/Complex	Free Ligand C=O Stretch (cm^{-1})	Complex C=O Stretch (cm^{-1})
**2**(*S,S*) / **7S**	1659	1618
**2**(*R,R*) / **8R**	1658	1618
**3**(*S,S*) / **9S**	1643	1618
**3**(*R,R*) / **10R**	1645	1619
**4**(*S,S*) / **11S**	1655	1619
**4**(*R,R*) / **12R**	1655	1619

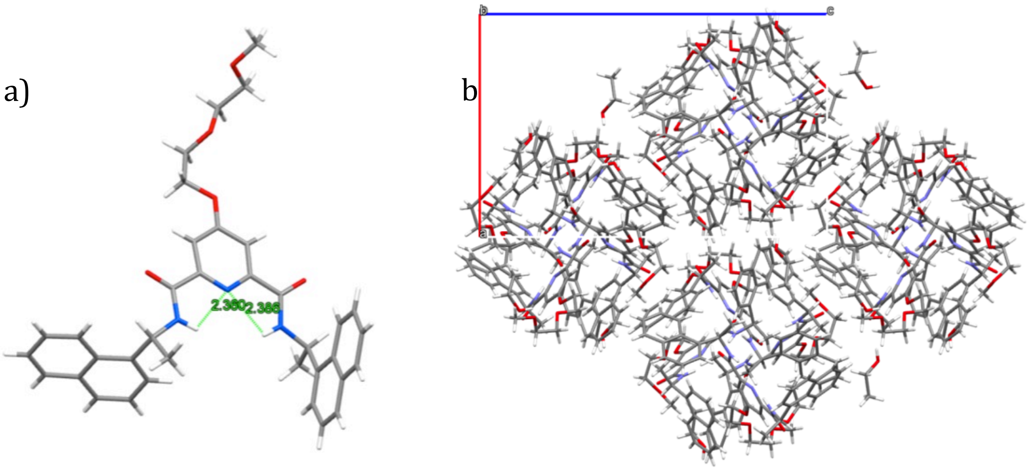

Figure S16: *a) X-ray crystal structure (capped stick) of ligand* ***4****(R,R) highlighting the hydrogen bonding between the amide NH protons and pyridyl nitrogen atom (second ligand molecule and solvent molecules omitted for clarity). b) X-ray packing diagram of* ***4****(R,R) viewed down the b-axis.*

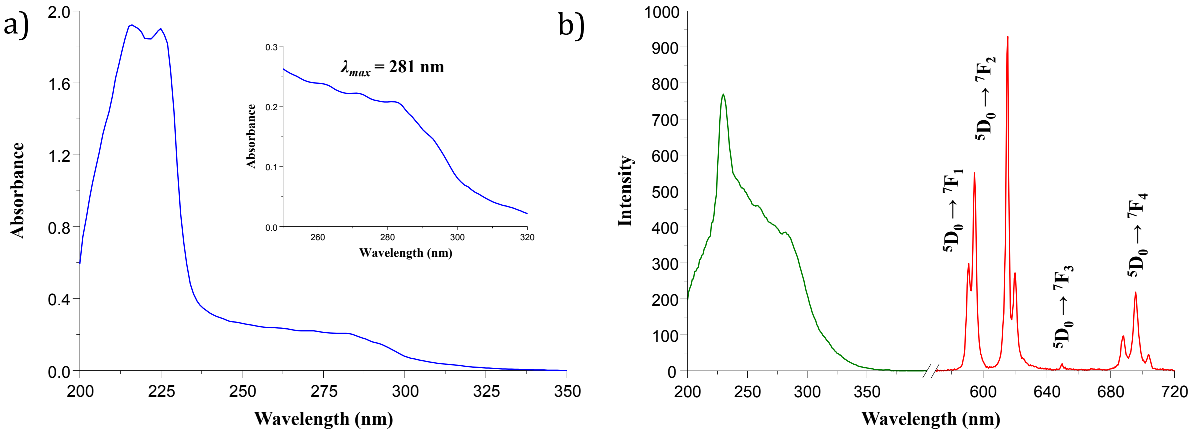

Figure S17: *a) UV-vis absorption and b) phosphorescence excitation (green) and emission (red) spectra (λ_{exc} = 281 nm) of* ***7S*** *(ca. 5.3 × 10^{-6} M) recorded in MeOH at 298 K.*

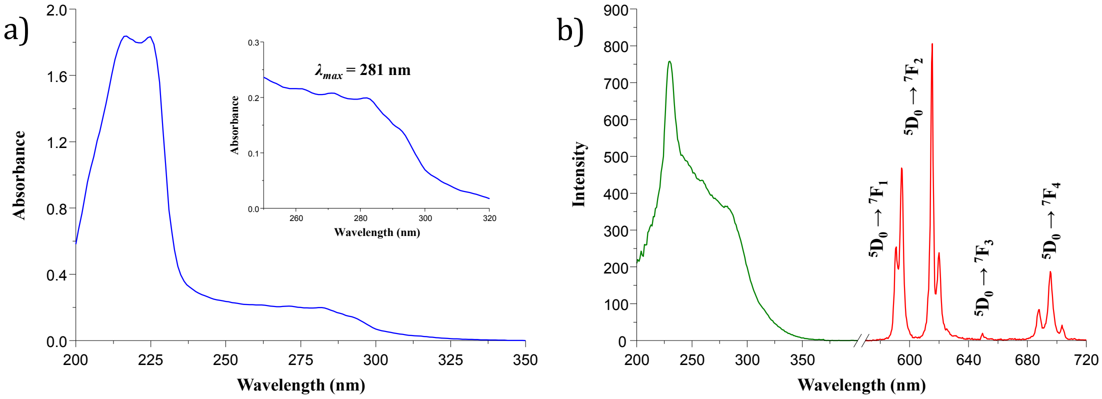

Figure S18: *a) UV-vis absorption and b) phosphorescence excitation (green) and emission (red) spectra (λ_{exc} = 281 nm) of* ***8R*** *(ca. 5.1 × 10^{-6} M) recorded in MeOH at 298 K.*

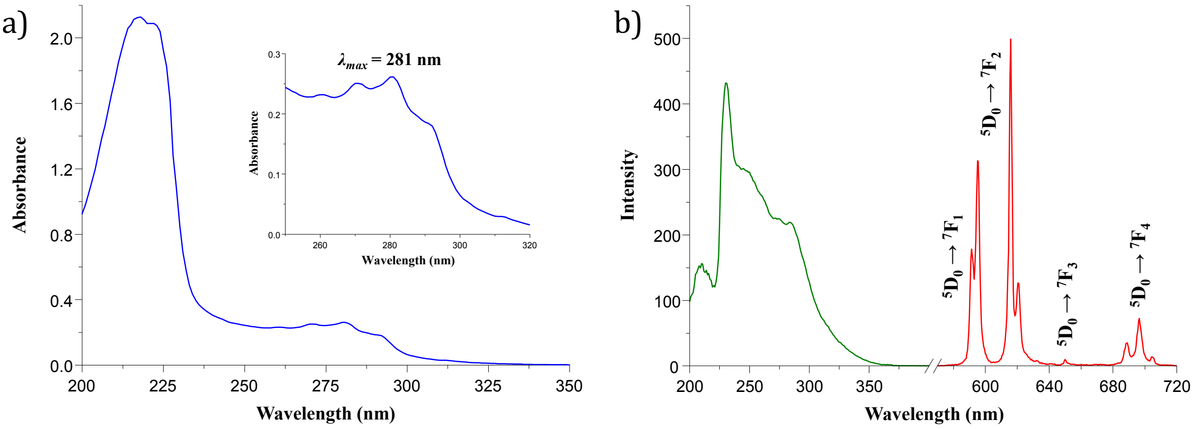

Figure S19: *a) UV-vis absorption and b) phosphorescence excitation (green) and emission (red) spectra (λ_{exc} = 281 nm) of* ***9S*** *(ca. 6.4 × 10^{-6} M) recorded in MeOH at 298 K.*

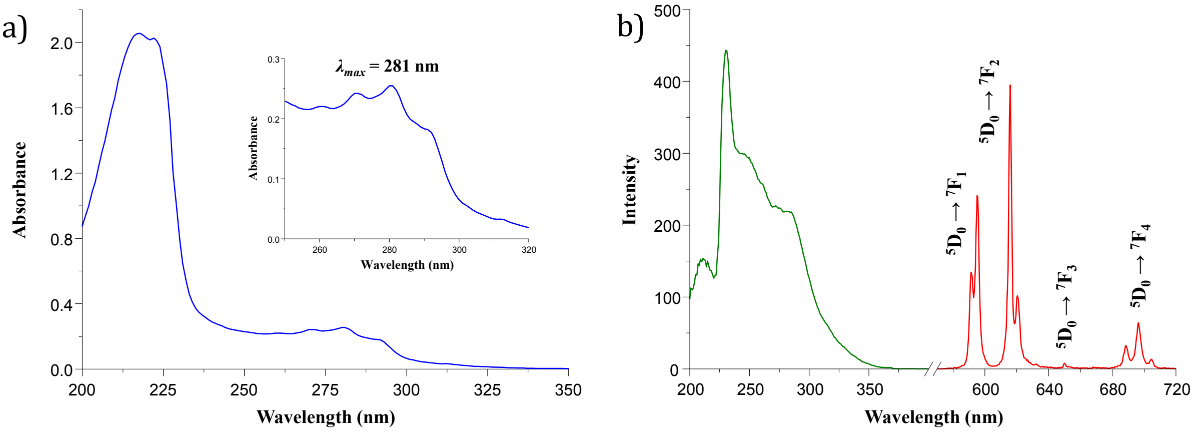

Figure S20: *a) UV-vis absorption and b) phosphorescence excitation (green) and emission (red) spectra (λ_{exc} = 281 nm) of* ***10R*** *(ca. 6.3 × 10^{-6} M) recorded in MeOH at 298 K.*

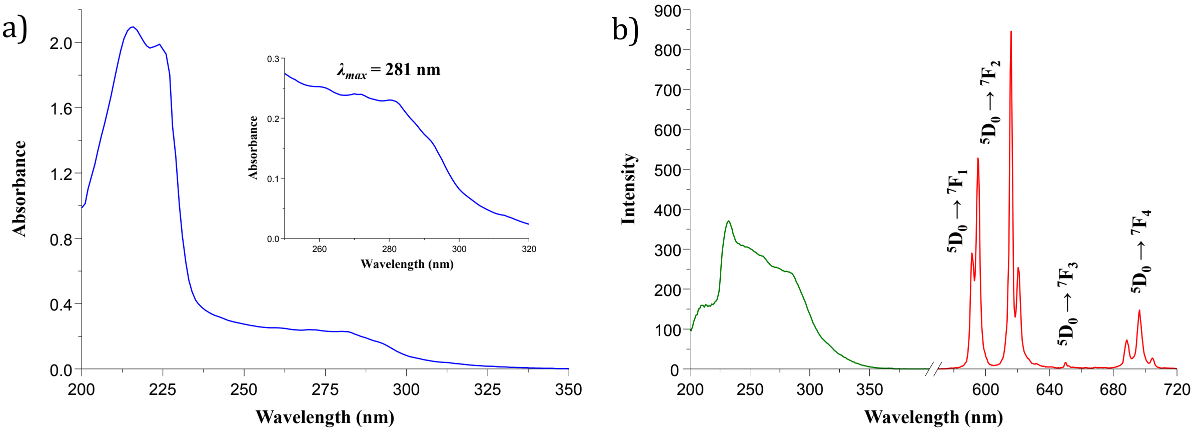

Figure S21: *a) UV-vis absorption and b) phosphorescence excitation (green) and emission (red) spectra (λ_{exc} = 281 nm) of* ***12R*** *(ca. 8.4 × 10^{-6} M) recorded in MeOH at 298 K.*

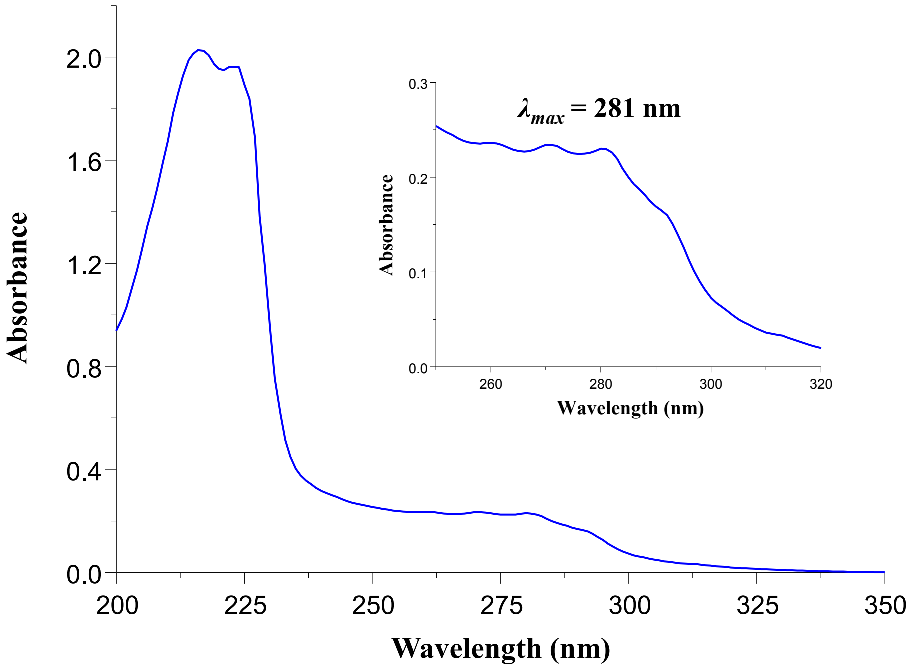

Figure S22: *The UV-vis absorption spectrum of **11S** (ca. 8.3 × 10*$^{-6}$ *M) recorded in MeOH at 298 K.*

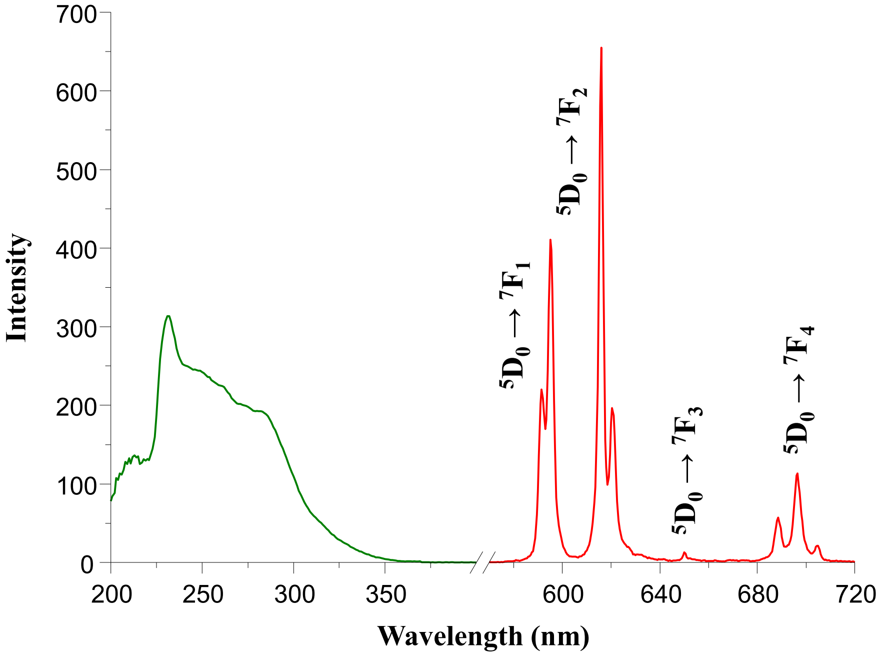

Figure S23. *Phosphorescence excitation (green) and emission (red) spectra (*λ_{exc} *= 281 nm) of **11S** (ca. 8.3 × 10*$^{-6}$ *M) recorded in MeOH at 298 K. Shown here in full.*

Table S3: *Lifetime measurements of the Eu(III) complexes* ***7S, 8R, 9S, 10R, 11S*** *and* ***12R*** *in water (H_2O and D_2O) and methanol (MeOH and MeOD) with their rate constants (k) and calculated q values. Each lifetime is an average of six measurements with different gate times.*

Complex	Solvent	$\tau_{O\text{-}H}$ (ms)	$\tau_{O\text{-}D}$ (ms)	$k_{O\text{-}H}$ (ms^{-1})	$k_{O\text{-}D}$ (ms^{-1})	q (± 0.5)
**7S**	Water	1.34 ± 0.02	1.61 ± 0.02	0.75	0.62	– 0.15
	Methanol	1.68 ± 0.01	4.48 ± 0.01	0.60	0.22	+ 0.24
**8R**	Water	1.43 ± 0.01	1.58 ± 0.02	0.70	0.63	– 0.22
	Methanol	1.67 ± 0.02	4.47 ± 0.01	0.60	0.22	+ 0.25
**9S**	Water	1.54 ± 0.01	2.33 ± 0.02	0.65	0.43	– 0.04
	Methanol	1.63 ± 0.03	4.20 ± 0.04	0.61	0.24	+ 0.25
**10R**	Water	1.52 ± 0.01	3.20 ± 0.04	0.66	0.31	+ 0.11
	Methanol	1.63 ± 0.02	4.01 ± 0.01	0.61	0.25	+ 0.23
**11S**	Water	1.51 ± 0.03	3.00 ± 0.09	0.66	0.33	+ 0.09
	Methanol	1.68 ± 0.01	4.47 ± 0.03	0.60	0.22	+ 0.24
**12R**	Water	1.57 ± 0.02	2.88 ± 0.10	0.64	0.35	+ 0.05
	Methanol	1.67 ± 0.01	4.37 ± 0.02	0.60	0.23	+ 0.24

Table S4: *Summary of the luminescence and intrinsic quantum yields, Eu(III) lifetimes and radiative lifetimes, and overall sensitisation efficiencies of Eu(III) complexes* ***5S, 6R, 7S, 8R, 9S, 10R, 11S*** *and* ***12R*** *in CH_3CN at 298 K. All data obtained from measurements of the same solutions.*

Complex	Q_L^{Ln} (%)	τ_{obs} (ms)	τ_{rad} (ms)	Q_{Ln}^{Ln} (%)	η_{sens} (%)
**5S**	4.21 ± 0.15	1.72 ± 0.01	10.63	16.16	26.06
**6R**	4.17 ± 0.10	1.72 ± 0.01	10.57	16.22	25.71
**7S**	4.44 ± 0.09	1.79 ± 0.03	9.41	19.03	23.34
**8R**	4.87 ± 0.14	1.78 ± 0.05	9.64	18.51	26.32
**9S**	5.75 ± 0.21	1.76 ± 0.05	9.46	18.61	30.90
**10R**	5.82 ± 0.26	1.76 ± 0.01	9.53	18.49	31.47
**11S**	4.56 ± 0.14	1.77 ± 0.02	9.54	18.52	24.62
**12R**	4.84 ± 0.11	1.73 ± 0.03	9.60	18.06	26.80

Table S5: *Summary of the luminescence and intrinsic quantum yields, Eu(III) lifetimes and radiative lifetimes, and overall sensitisation efficiencies of* ***11S*** *and* ***12R*** *in CH_3CN, MeOH, and H_2O/MeOH (1:1; v/v) at 298 K. All data obtained from measurements of the same solutions.*

Solvent	Complex	Q_L^{Ln} (%)	τ_{obs} (ms)	τ_{rad} (ms)	Q_{Ln}^{Ln} (%)	η_{sens} (%)
**CH_3CN**	**11S**	4.56 ± 0.14	1.77 ± 0.02	9.54	18.52	24.62
	**12R**	4.84 ± 0.11	1.73 ± 0.03	9.60	18.06	26.80
**MeOH**	**11S**	4.37 ± 0.15	1.67 ± 0.01	10.56	15.86	27.56
	**12R**	4.18 ± 0.19	1.66 ± 0.03	10.51	15.82	26.42
**H_2O/MeOH (1:1; *v/v*)**	**11S**	7.25 ± 0.25	1.67 ± 0.01	10.24	16.35	44.33
	**12R**	7.39 ± 0.27	1.64 ± 0.01	10.33	15.88	46.53

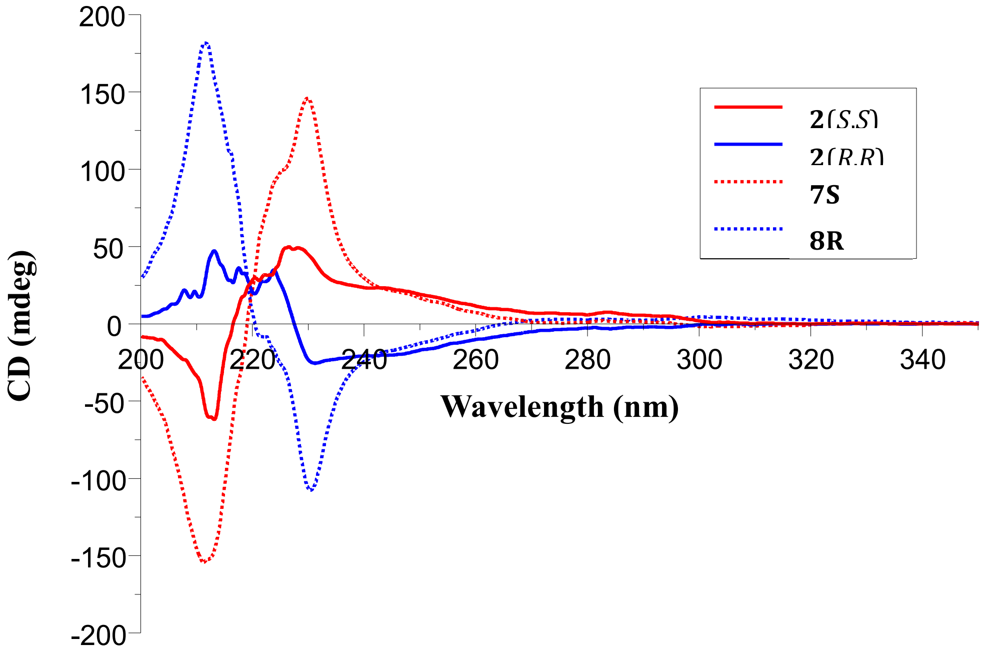

Figure S24: *CD spectra of ligand solutions (2.0 × 10^{-5} M) of* ***2****(S,S) and* ***2****(R,R) recorded in CH_3CN at 298 K before (solid lines) and after (dashed lines) the addition of 0.33 eq. of $Eu(CF_3SO_3)_3$ to give the complexes and* ***7S*** *and* ***8R****, respectively.*

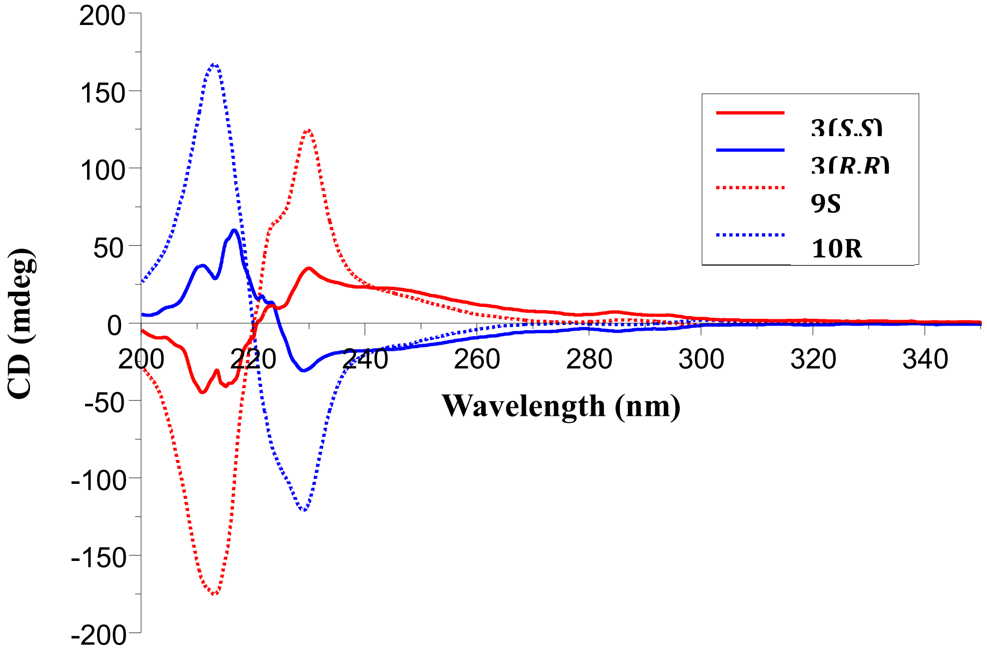

Figure S25: *CD spectra of ligand solutions (2.0 × 10^{-5} M) of* ***3(S,S)*** *and* ***3(R,R)*** *recorded in CH_3CN at 298 K before (solid lines) and after (dashed lines) the addition of 0.33 eq. of $Eu(CF_3SO_3)_3$ to give the complexes* ***9S*** *and* ***10R****, respectively.*

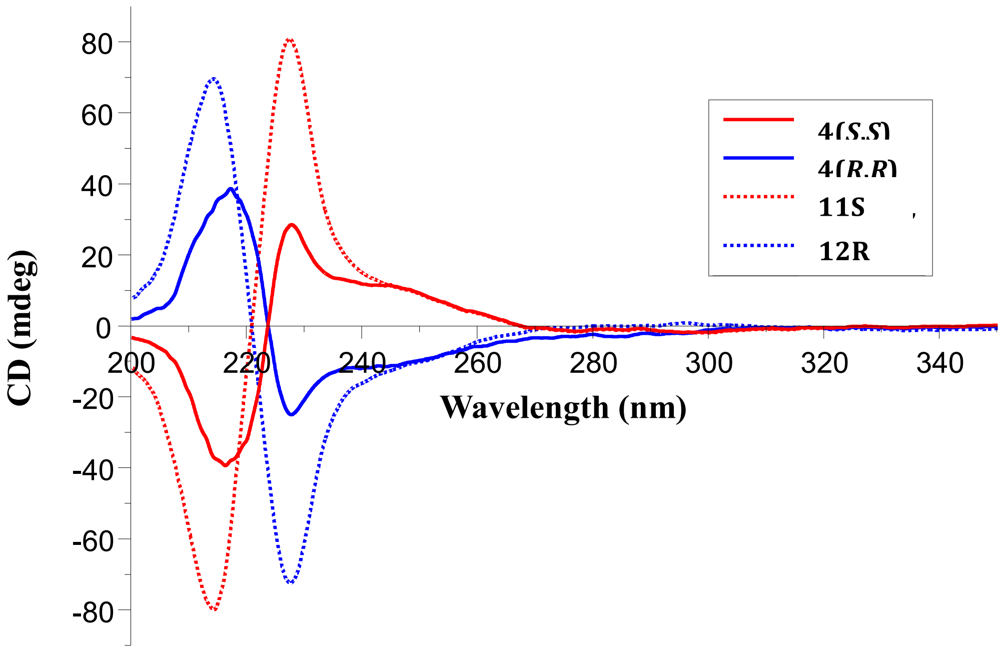

Figure S26: *CD spectra of ligand solutions (1.7 × 10^{-5} M) of* ***4(S,S)*** *and* ***4(R,R)*** *recorded in CH_3CN at 298 K before (solid lines) and after (dashed lines) the addition of 0.33 eq. of $Eu(CF_3SO_3)_3$ to give the complexes* ***11S*** and ***12R****, respectively.*

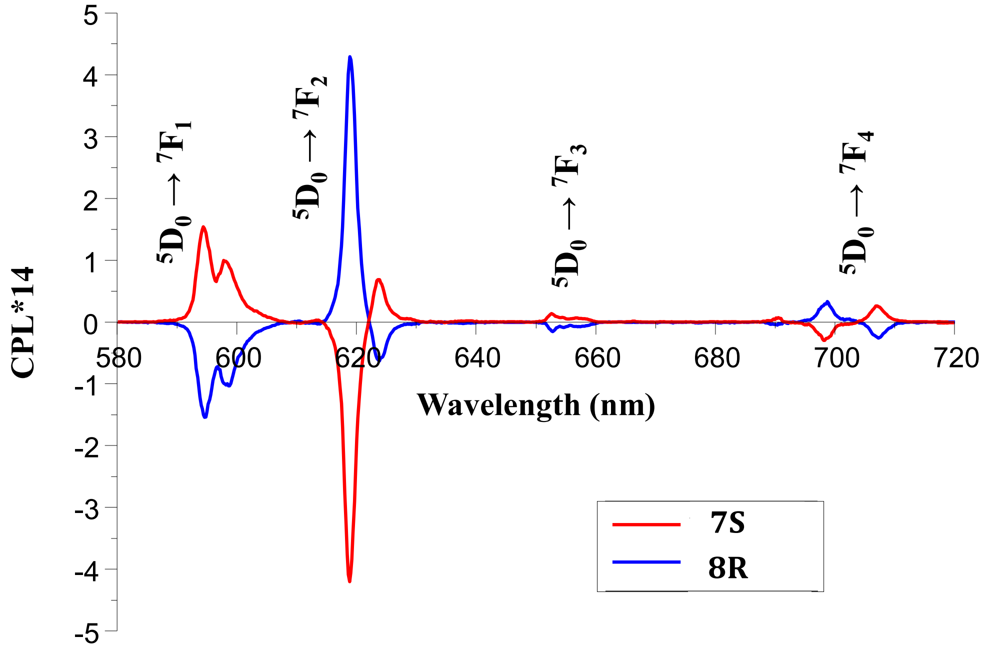

Figure S27: *CPL emission spectra of* ***7S*** *and* ***8R****, recorded in CH_3CN at 298 K (λ_{exc} = 281 nm).*

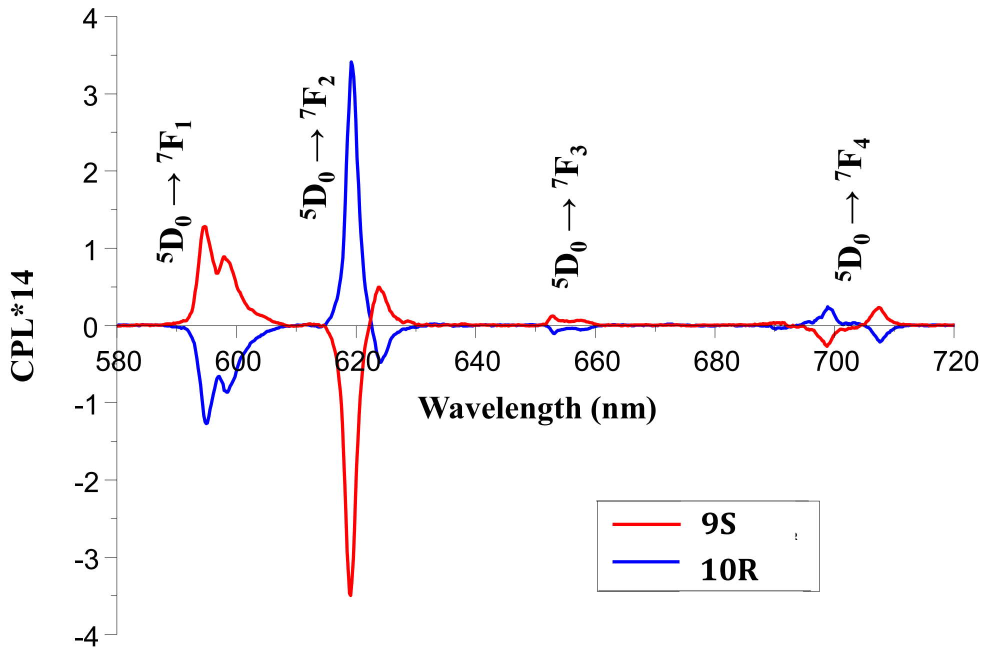

Figure S28: *CPL emission spectra of* ***9S*** *and* ***10R****, recorded in* CH_3CN *at 298 K (*λ_{exc} *= 281 nm).*

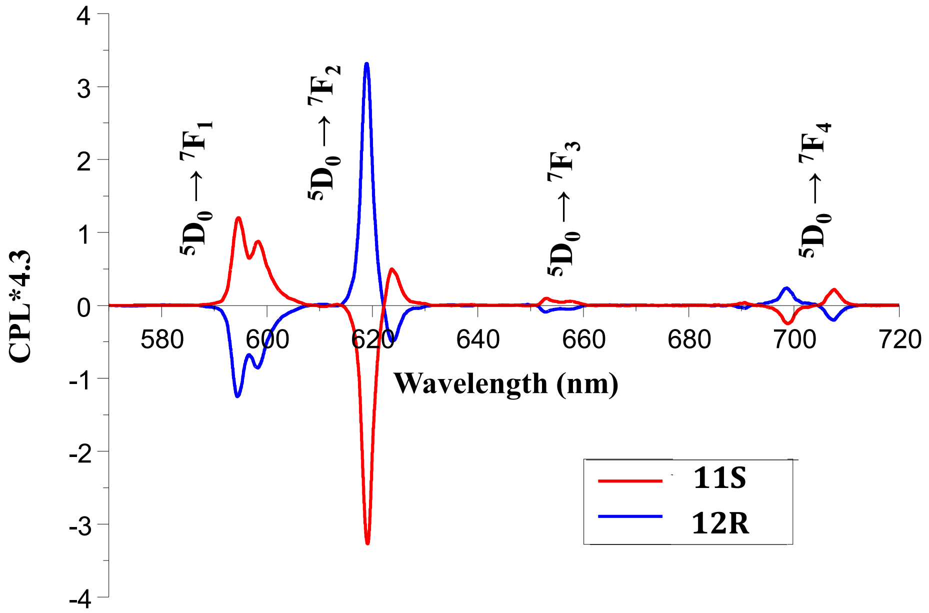

Figure S29: *CPL emission spectra of* ***11S*** *and* ***12R****, recorded in* CH_3CN *at 298 K (*λ_{exc} *= 281 nm).*

Table S6: *Summary of the g_{lum} dissymmetry factor values calculated from the CPL spectra of Eu(III) complexes **7S, 8R, 9S, 10R, 11S** and **12R** for the J = 1 and 2 transitions at 595 nm and 619 nm, respectively.*

Complex	g_{lum} value 595 nm (J = 1)	g_{lum} value 619 nm (J = 2)
**7S**	+ 0.31	− 0.26
**8R**	− 0.29	+ 0.22
**9S**	+ 0.27	− 0.23
**10R**	− 0.27	+ 0.21
**11S**	+ 0.31	− 0.26
**12R**	− 0.32	+ 0.27

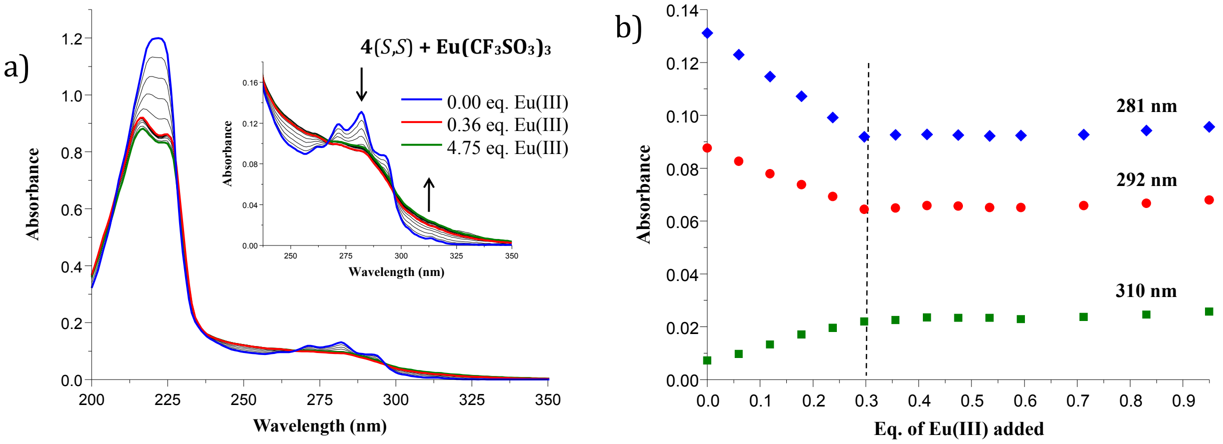

Figure S30: *a) Changes in the absorption spectra of* ***4****(S,S) (1 × 10^{-5} M) upon titrating with Eu(CF_3SO_3)$_3$ (0.00 → 4.75 eq.) in CH_3CN at 298 K. b) Binding isotherm for the titration measured at 281 nm, 292 nm, and 310 nm.*

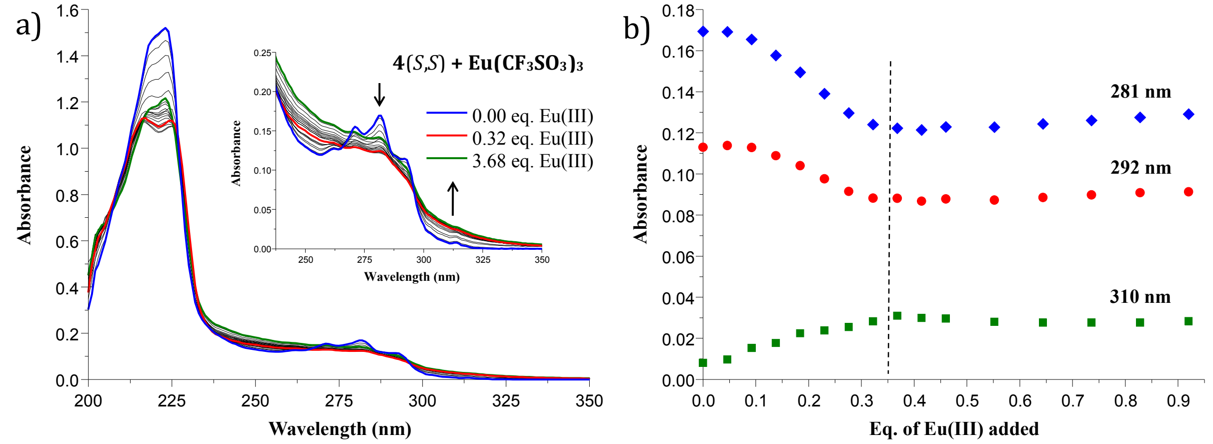

Figure S31: *a) Changes in the absorption spectra of* ***4****(S,S) (1 × 10^{-5} M) upon titrating with Eu(CF_3SO_3)$_3$ (0.00 → 3.68 eq.) in MeOH at 298 K. b) Binding isotherm for the titration measured at 281 nm, 292 nm, and 310 nm.*

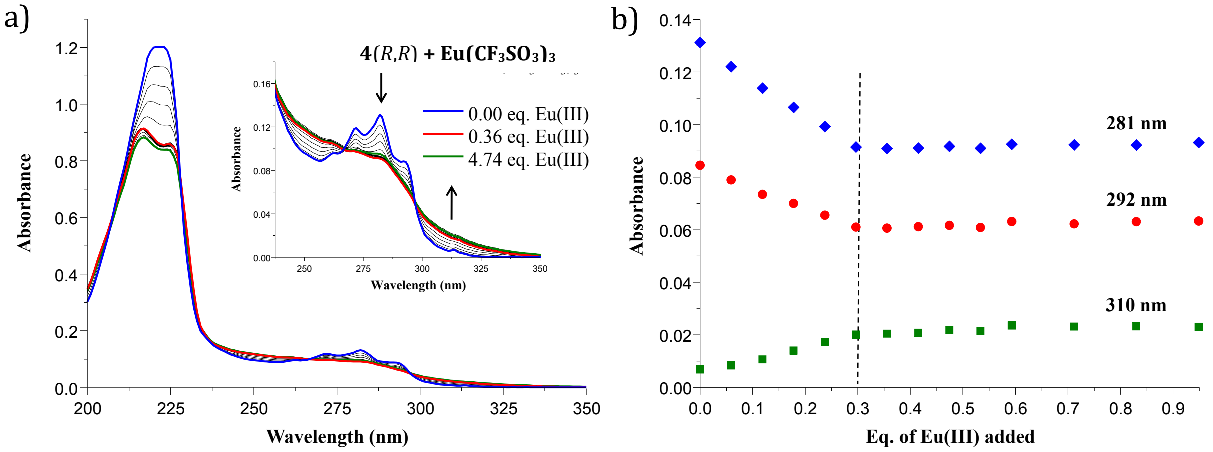

Figure S32: *a) Changes in the absorption spectra of* ***4****(R,R) (1 × 10^{-5} M) upon titrating with Eu(CF_3SO_3)$_3$ (0.00 → 4.74 eq.) in CH_3CN at 298 K. b) Binding isotherms for the titration measured at 281 nm, 292 nm, and 310 nm.*

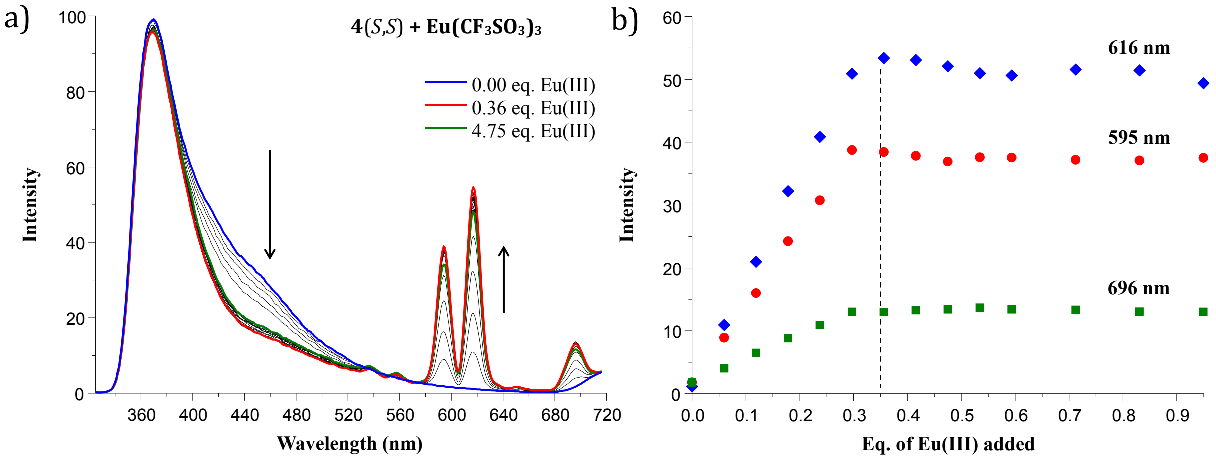

***Figure S33:** a) Changes in the fluorescence spectra of **4**(S,S) (1 × 10^{-5} M) upon titrating with Eu(CF_3SO_3)$_3$ (0.00 → 4.75 eq.) in CH_3CN at 298 K. b) Binding isotherms for the titration measured at 595 nm, 616 nm, and 696 nm.*

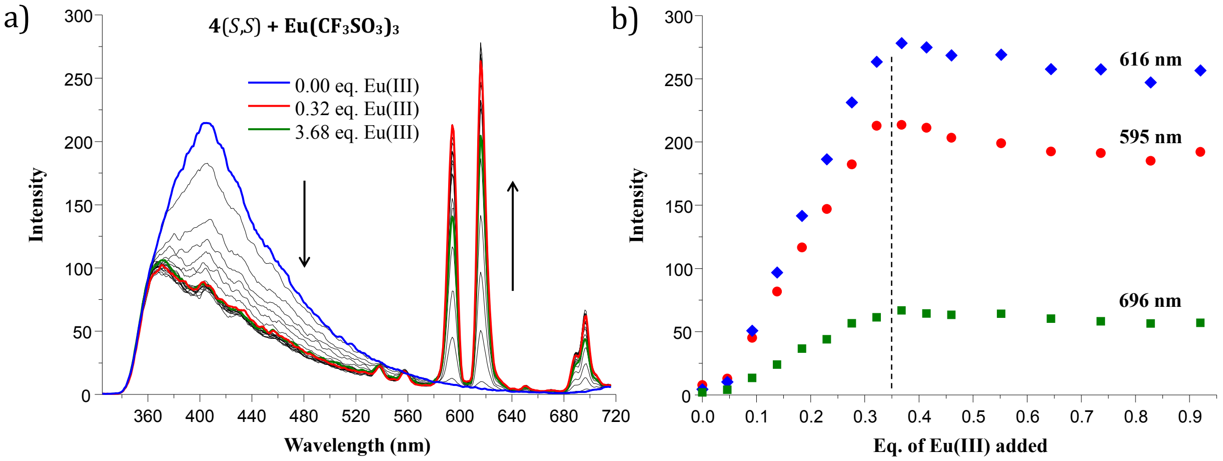

***Figure S34:** a) Changes in the fluorescence spectra of **4**(S,S) (1 × 10^{-5} M) upon titrating with Eu(CF_3SO_3)$_3$ (0.00 → 3.68 eq.) in MeOH at 298 K. b) Binding isotherms for the titration measured at 595 nm, 616 nm, and 696 nm.*

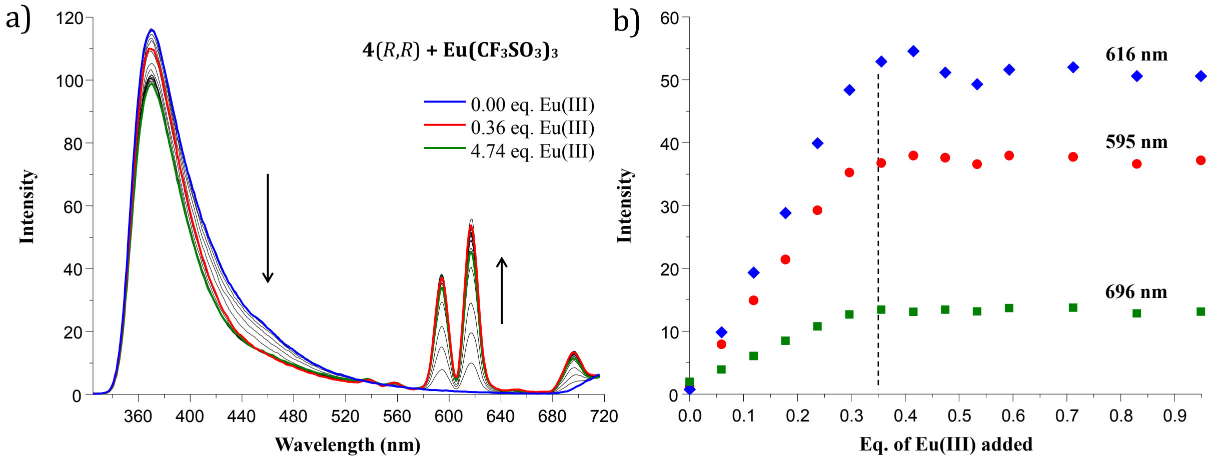

***Figure S35:** a) Changes in the fluorescence spectra of **4**(R,R) (1 × 10^{-5} M) upon titrating with Eu(CF_3SO_3)$_3$ (0.00 → 4.74 eq.) in CH_3CN at 298 K. b) Binding isotherms for the titration measured at 595 nm, 616 nm, and 696 nm.*

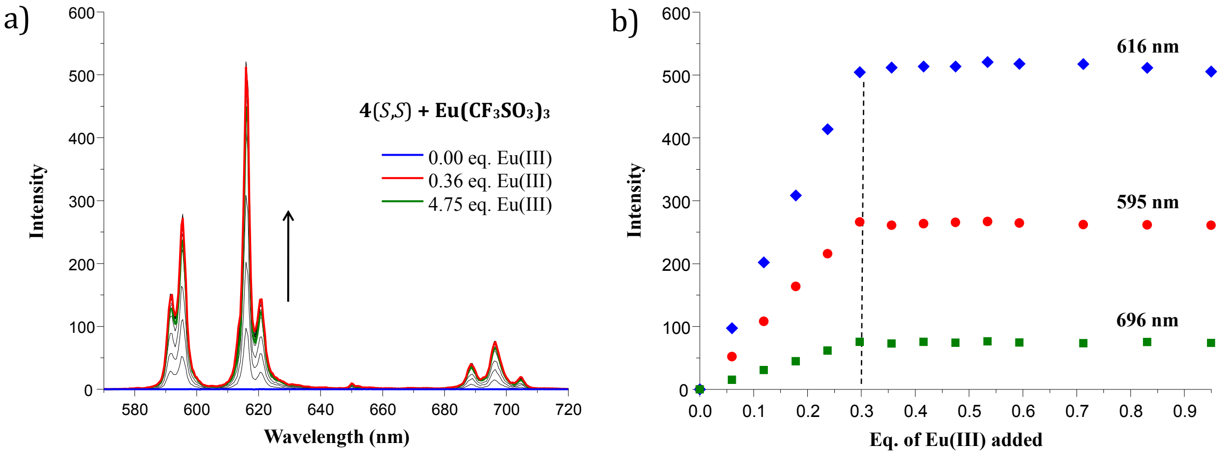

***Figure S36**: a) Evolution of the phosphorescence emission in the titration of **4**(S,S) (1 × 10⁻⁵ M) with Eu(CF₃SO₃)₃ (0.00 → 4.75 eq.) in CH₃CN at 298 K (λ_exc = 281 nm). b) Binding isotherm for the titration measured at 595 nm, 616 nm, and 696 nm.*

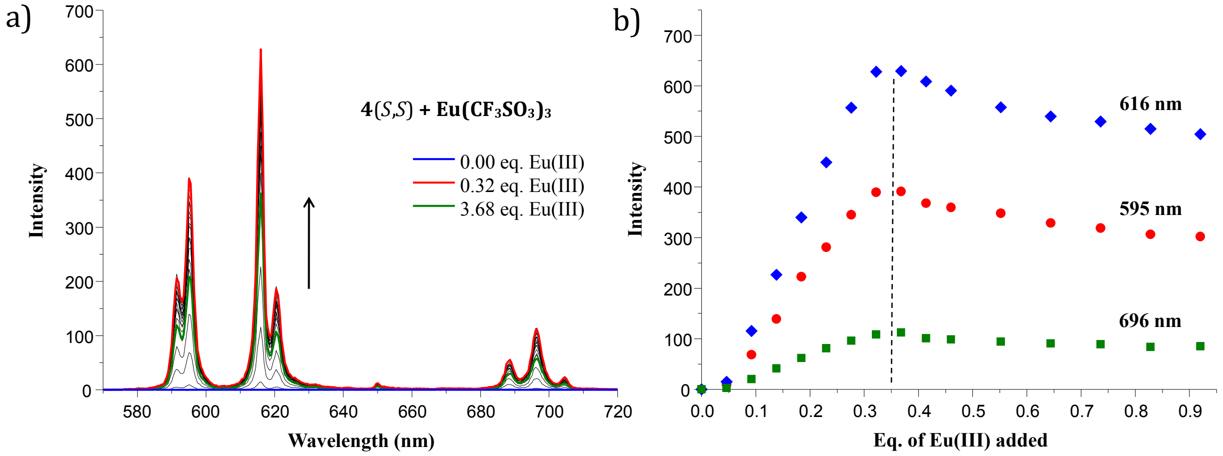

***Figure S37:** a) Evolution of the phosphorescence emission in the titration of **4**(S,S) (1 × 10⁻⁵ M) with Eu(CF₃SO₃)₃ (0.00 → 3.68 eq.) in MeOH at 298 K (λ_exc = 281 nm). b) Binding isotherm for the titration measured at 595 nm, 616 nm, and 696 nm.*

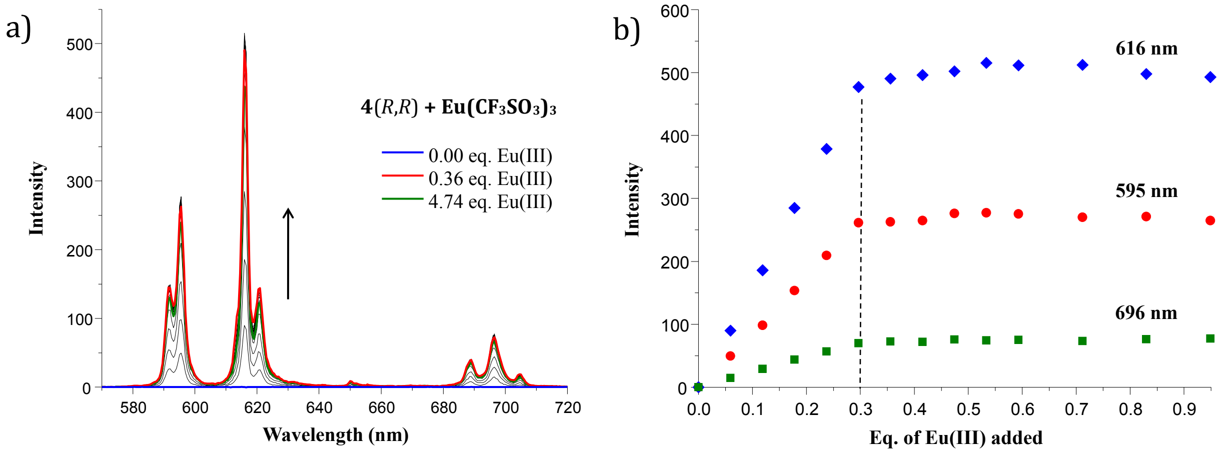

***Figure S38:** a) Evolution of the phosphorescence emission in the titration of **4**(R,R) (1 × 10⁻⁵ M) with Eu(CF₃SO₃)₃ (0.00 → 4.74 eq.) in CH₃CN at 298 K (λ_exc = 281 nm). b) Binding isotherms for the titration measured at 595 nm, 616 nm, and 696 nm.*

Table S7: *Summary of the binding constants obtained through data fitting for the UV/vis and phosphorescence titrations of* ***1-4*** *(1 × 10^{-5} M) with Eu(CF_3SO_3)$_3$ in CH_3CN, with percentage composition of the 1:3 (M:L) species at 0.33 eq. of Eu(III). *Value fixed.*

Ligand	Mode	log $\beta_{1:3}$	log $\beta_{1:2}$	log $\beta_{1:1}$	% of 1:3 (M:L) species at 0.33 eq. of Eu(III)
**1S**	**UV**	21.84 ± 0.87	–	7.76 ± 0.49	93
	**Pho**	21.48 ± 0.00*	–	6.89 ± 0.79	94
**1R**	**UV**	21.71 ± 0.65	–	7.49 ± 0.33	92
	**Pho**	21.48 ± 0.50	–	7.00 ± 0.31	93
**2S**	**UV**	23.25 ± 0.89	15.41 ± 0.77	8.04 ± 0.52	90
	**Pho**	22.95 ± 0.38	15.30 ± 0.00*	8.17 ± 0.22	90
**2R**	**UV**	22.12 ± 0.71	15.30 ± 0.61	7.41 ± 0.38	80
	**Pho**	22.12 ± 0.00*	14.85 ± 0.29	7.72 ± 0.18	86
**3S**	**UV**	–	–	–	–
	**Pho**	21.99 ± 0.61	13.75 ± 0.87	7.38 ± 0.35	93
**3R**	**UV**	21.51 ± 0.80	–	6.93 ± 0.44	94
	**Pho**	21.72 ± 0.00*	14.28 ± 0.35	7.23 ± 0.20	87
**4S**	**UV**	19.41 ± 0.36	–	6.35 ± 0.18	85
	**Pho**	19.86 ± 0.27	–	7.06 ± 0.18	83
**4R**	**UV**	20.60 ± 0.55	–	7.01 ± 0.27	89
	**Pho**	20.50 ± 0.31	–	7.03 ± 0.20	89

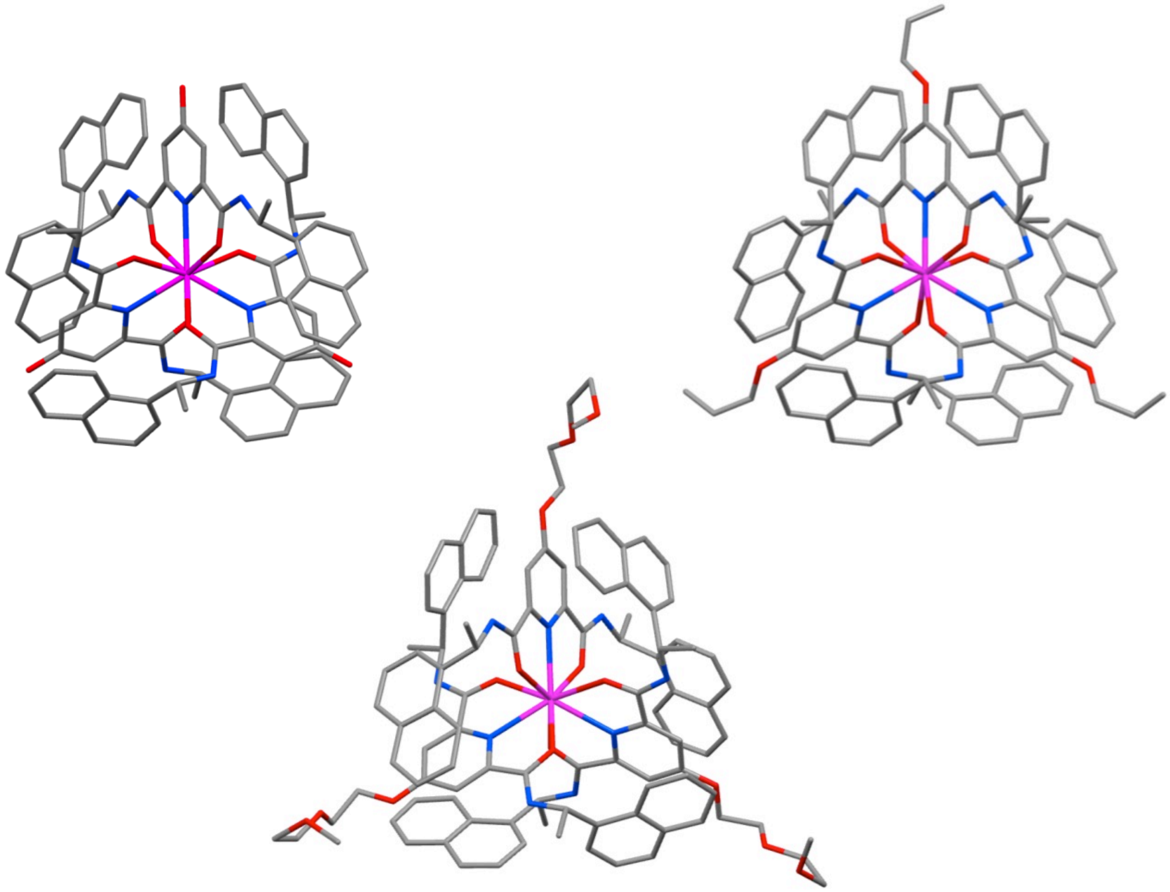

Figure S39: *X-ray crystal structures (capped stick) of the Eu(III) complexes a)* ***6R****, b)* ***7S****, and c)* ***11S*** *showing the helical arrangement of ligands around the central Eu(III) Son (counter-anions and solvent molecules omitted for clarity).*

a) b)

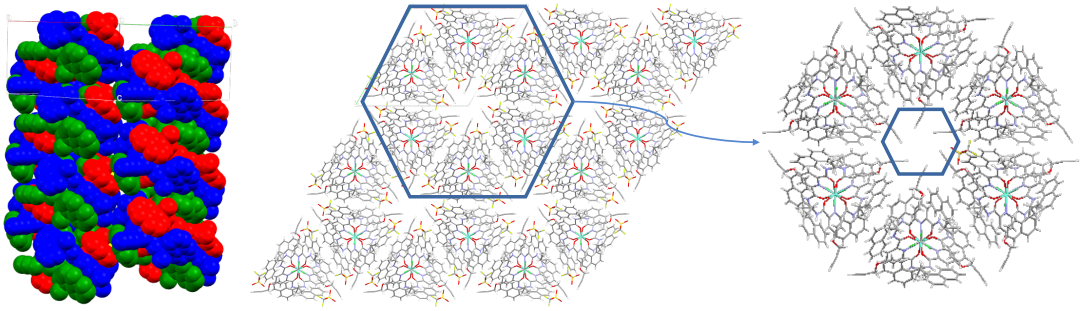

Figure S40: *a) X-ray packing diagram (space-filled) of* ***10R*** *propagated down the c-axis. b) X-ray packing diagram (capped stick) of* ***10R*** *viewed down the c-axis showing both the orientation of the ligand propargyl chains into a central cavity (as seen for* ***11S*** *in the main body of the article).*

Table S8: *Summary of the Eu-O and Eu-N bond lengths procured from the X-ray crystal structures of* ***5S****,* ***6R****,* ***7S****, and* ***11S****.*

Bond	Bond Length (Å)			
	5S	6R	7S	11S
**Eu-O**	2.415	2.413	2.415	2.414
**Eu-O**	2.415	2.413	2.415	2.415
**Eu-O**	2.415	2.413	2.415	2.415
**Eu-O**	2.415	2.413	2.415	2.422
**Eu-O**	2.415	2.413	2.415	2.422
**Eu-O**	2.415	2.413	2.415	2.422
**Eu-N**	2.520	2.520	2.529	2.545
**Eu-N**	2.520	2.520	2.529	2.545
**Eu-N**	2.520	2.520	2.529	2.546

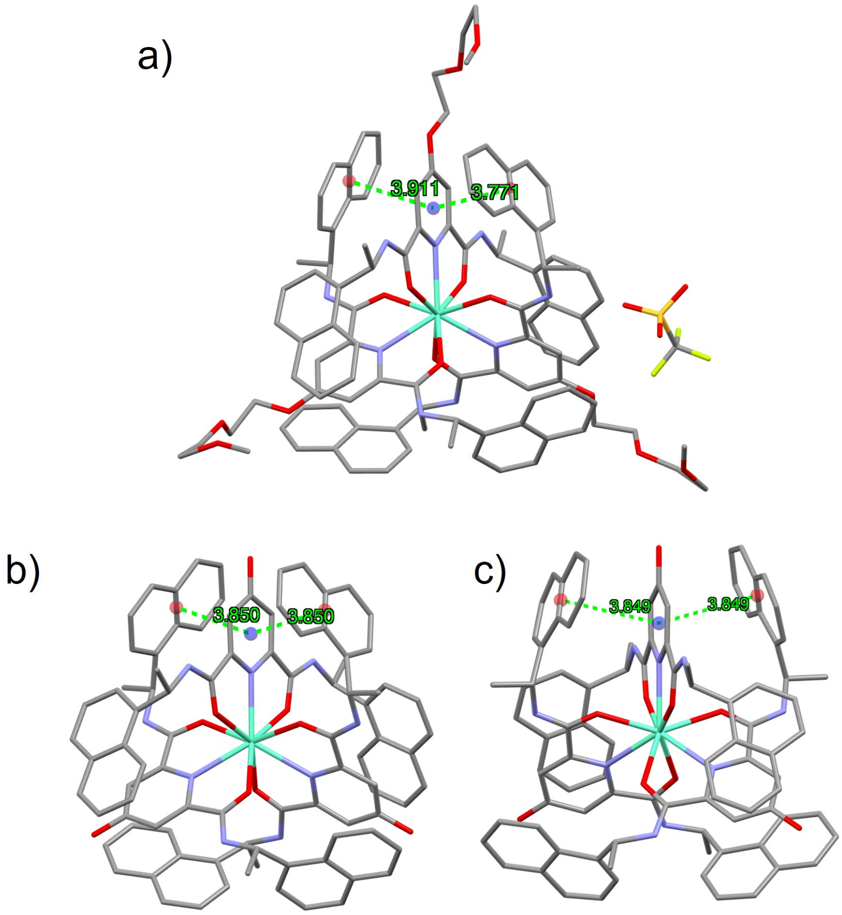

Figure S41*: X-ray crystal structures (capped stick) of the Eu(III) complexes showing the stabilization of Bundle-type structure of the complexes due to the π-π stacking interactions between the pyridine ring of each ligand molecule intercalated between the naphthyl groups of the other two a)* ***11S****, b)* ***5S*** *and c)* ***6R****.*

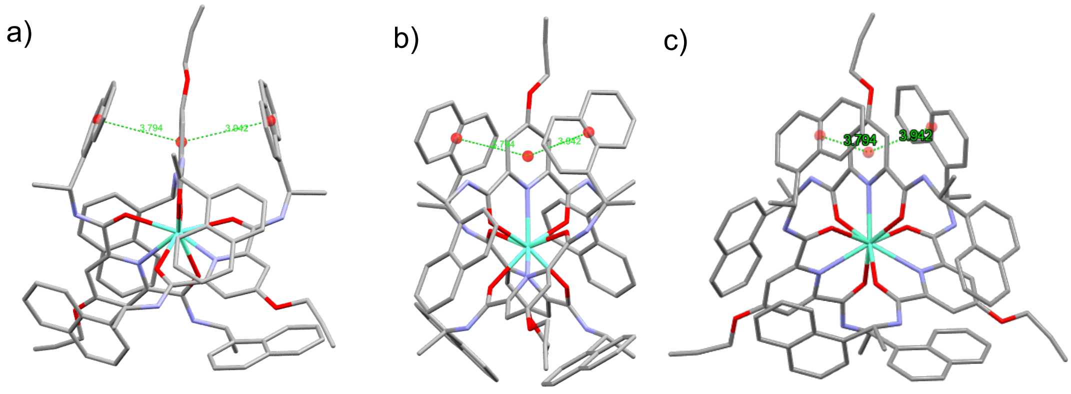

***Figure S42**: X-ray crystal structures (capped stick) of the Eu(III) complex **10R** showing the stabilization of Bundle-type structure of the complexes due to the π-π stacking interactions between the pyridine ring of each ligand molecule intercalated between the naphthyl groups.*

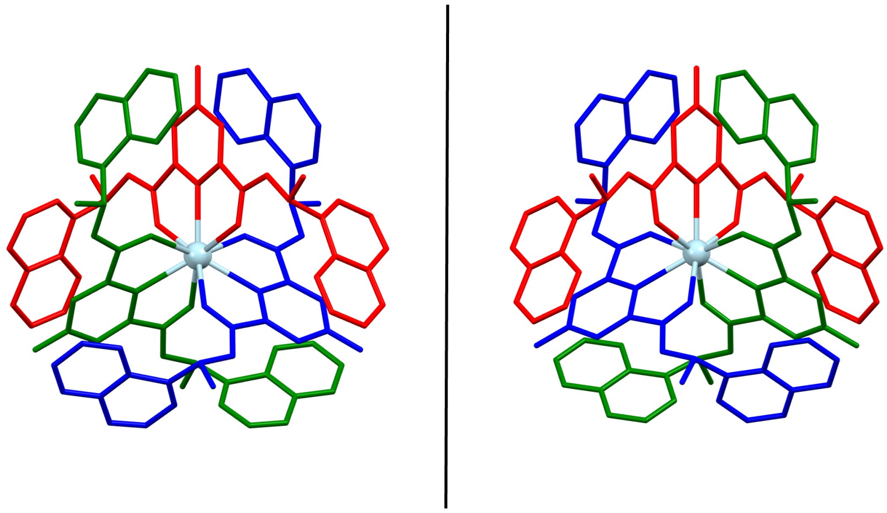

6R **5S**

***Figure S43:** Representation of **6R** and **5S**, showing that are clearly mirror images of each other.*

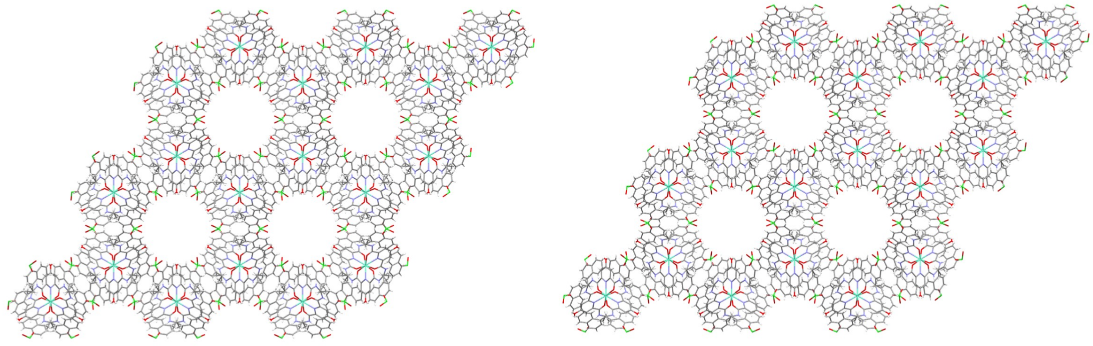

6R **5S**

Figure S44: *X-ray packing diagram (capped stick) of* ***6R*** *and* ***5S*** *viewed down the c-axis showing both the presence of channels.*

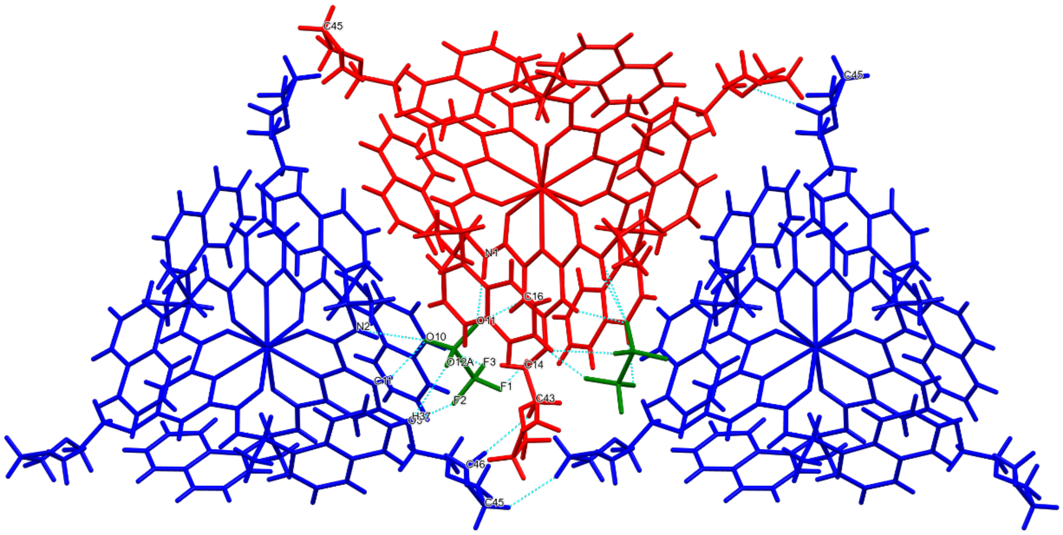

Figure S45: *Crystal structure of* ***11S*** *showing that the packing of the complexes is stabilized by the interaction with $CF_3SO_3^-$ counter anions.*

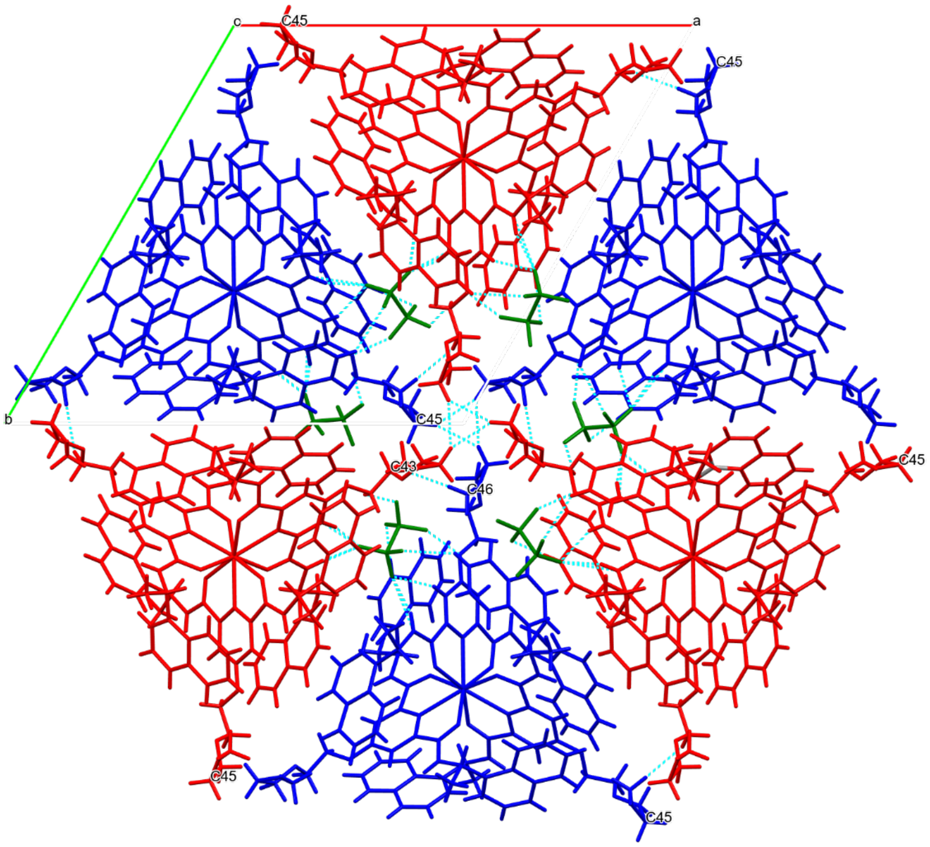

Figure S46: *Crystal structure of* ***11S*** *showing that the 2-(2-methoxy-ethoxy)ethane chains oriented towards the cavity interact through CH···CH soft contacts of the C45 and two C45 from neighbouring complexes.*

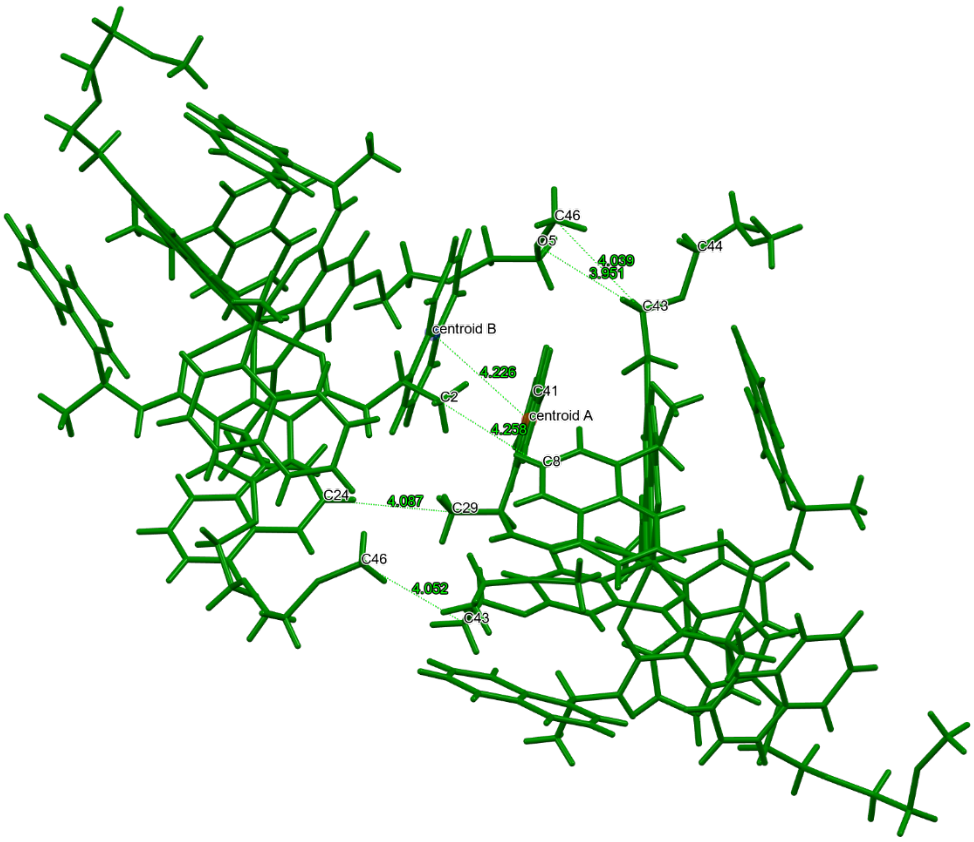

Figure S47: *Crystal structure of* ***11S*** *showing that the helical packing of the complexes within the crystal structure is due to the interaction between the two naphthalene rings of neighboring complexes and other soft contacts.*

***Table S9:** Crystal data and structure refinement for **4**(R,R).*

Empirical formula	$C_{76}H_{82}N_6O_{12}$	
Formula weight	1271.47	
Temperature	293(2) K	
Wavelength	0.71073 Å	
Crystal system	Orthorhombic	
Space group	P 21 21 21	
Unit cell dimensions	a = 14.906(5) Å	α= 90.000(5)°.
	b = 19.311(5) Å	β= 90.000(5)°.
	c = 23.190(5) Å	γ = 90.000(5)°.
Volume	6675(3) $Å^3$	
Z	4	
Density (calculated)	1.265 Mg/m^3	
Absorption coefficient	0.086 mm^{-1}	
F(000)	2704	
Crystal size	0.10 x 0.18 x 0.27 mm^3	
Theta range for data collection	1.372 to 27.861°.	
Index ranges	-19<=h<=19, -25<=k<=25, -29<=l<=30	
Reflections collected	73933	
Independent reflections	73933 [R(int) = ?]	
Completeness to theta = 25.242°	99.9 %	
Refinement method	Full-matrix least-squares on F^2	
Data / restraints / parameters	73933 / 7 / 847	
Goodness-of-fit on F^2	1.005	
Final R indices [I>2sigma(I)]	R_1 = 0.0681, wR_2 = 0.1857	
R indices (all data)	R_1 = 0.0821, wR_2 = 0.2015	
Absolute structure parameter	-0.15(15)	
Largest diff. peak and hole	1.125 and -0.686 e.$Å^{-3}$	

Table S10:** Crystal data and structure refinement for **5S.

Empirical formula	$C_{93}H_{81}Cl_3EuN_9O_{21}$	
Formula weight	1918.97	
Temperature	293(2) K	
Wavelength	71.073 pm	
Crystal system	Hexagonal	
Space group	P 63 2 2	
Unit cell dimensions	a = 2297.7(4) pm	α= 90°.
	b = 2297.7(4) pm	β= 90°.
	c = 1293.6(2) pm	γ = 120°.
Volume	5.92(1) nm^3	
Z	2	
Density (calculated)	1.078 Mg/m^3	
Absorption coefficient	0.656 mm^{-1}	
F(000)	1968	
Crystal size	0.15 x 0.18 x 0.26 mm^3	
Theta range for data collection	1.773 to 26.379°.	
Index ranges	-27<=h<=27, -28<=k<=28, -16<=l<=16	
Reflections collected	83846	
Independent reflections	83846 [R(int) = ?]	
Completeness to theta = 25.242°	99.9 %	
Refinement method	Full-matrix least-squares on F^2	
Data / restraints / parameters	83846 / 74 / 170	
Goodness-of-fit on F^2	0.869	
Final R indices [I>2sigma(I)]	R_1 = 0.0636, wR_2 = 0.1906	
R indices (all data)	R_1 = 0.0730, wR_2 = 0.2037	
Absolute structure parameter	-0.015(10)	
Largest diff. peak and hole	1.510 and -0.771 e.Å^{-3}	

Table S11: *Crystal data and structure refinement for* ***6R****.*

Empirical formula	$C_{62}H_{54}Cl_2Eu_{0.67}N_6O_{14}$	
Formula weight	1279.32	
Temperature	296(2) K	
Wavelength	0.71073 Å	
Crystal system	Hexagonal	
Space group	P 63 2 2	
Unit cell dimensions	a = 22.917(4) Å	α= 90°.
	b = 22.917(4) Å	β= 90°.
	c = 12.943(2) Å	γ = 120°.
Volume	5887(2) $Å^3$	
Z	3	
Density (calculated)	1.083 Mg/m^3	
Absorption coefficient	0.660 mm^{-1}	
F(000)	1968	
Crystal size	0.181 x 0.160 x 0.124 mm^3	
Theta range for data collection	1.777 to 26.018°.	
Index ranges	-25<=h<=28, -28<=k<=28, -15<=l<=15	
Reflections collected	41577	
Independent reflections	41577 [R(int) = ?]	
Completeness to theta = 25.242°	99.9 %	
Refinement method	Full-matrix least-squares on F^2	
Data / restraints / parameters	41577 / 43 / 183	
Goodness-of-fit on F^2	0.884	
Final R indices [I>2sigma(I)]	R_1 = 0.0599, wR_2 = 0.1790	
R indices (all data)	R_1 = 0.0680, wR_2 = 0.1901	
Absolute structure parameter	-0.016(9)	
Largest diff. peak and hole	1.370 and -0.887 e.$Å^{-3}$	

Table S12: *Crystal data and structure refinement for* ***7S****.*

Empirical formula	$C_{100}H_{100}Cl_3EuF_9N_9O_{15}$	
Formula weight	2097.20	
Temperature	100(2) K	
Wavelength	1.54178 Å	
Crystal system	Monoclinic	
Space group	$P6_322$	
Unit cell dimensions	a = 22.880(6) Å	α = 90°
	b = 22.880(6) Å	β = 90°
	c = 12.981(4) Å	γ = 120°
Volume	5885(3) $Å^3$	
Z	2	
Density (calculated)	1.184 mg m^{-3}	
Absorption coefficient	5.043 mm^{-1}	
F(000)	2156	
Crystal size	0.36 × 0.07 × 0.04 mm	
Theta range for data collection	3.86 – 64.45°	
Index ranges	−25 ≤ h ≤ 24, −23 ≤ k ≤ 20, −14 ≤ l ≤ 15	
Reflections collected	14291	
Independent reflections	3255 [R(int) = 0.0464]	
Completeness to theta = 64.45°	98.9%	
Absorption correction	None	
Refinement method	Full-matrix least-squares on F^2	
Data / restraints / parameters	3255 / 4 / 169	
Goodness-of-fit on F^2	1.048	
Final R indices [I>2sigma(I)]	R1 = 0.0820, wR2 = 0.2283	
R indices (all data)	R1 = 0.0900, wR2 = 0.2384	
Largest diff. peak and hole	0.711 and −0.607 e.$Å^{-3}$	

Table S13: *Crystal data and structure refinement for* ***9S****.*

Empirical formula	C105.32H84.32Cl0.95EuF9N9O18S3
Formula weight	2216.75
Temperature/K	100.00
Crystal system	hexagonal
Space group	P63
a/Å	22.8424(2)
b/Å	22.8424(2)
c/Å	12.9269(2)
α/°	90
β/°	90
γ/°	120
Volume/Å3	5841.28(14)
Z	2
ρcalcg/cm3	1.260
μ/mm-1	0.689
F(000)	2263.0
Crystal size/mm3	0.662 × 0.079 × 0.066
Radiation	MoKα (λ = 0.71073)
2Θ range for data collection/°	5.186 to 56.626
Index ranges	-30 ≤ h ≤ 30, -30 ≤ k ≤ 30, -17 ≤ l ≤ 17
Reflections collected	151807
Independent reflections	9667 [Rint = 0.0576, Rsigma = 0.0345]
Data/restraints/parameters	9667/1589/539
Goodness-of-fit on F2	1.045
Final R indexes [I>=2σ (I)]	R1 = 0.0715, wR2 = 0.1941
Final R indexes [all data]	R1 = 0.0853, wR2 = 0.2075
Largest diff. peak/hole / e Å-3	1.57/-0.81
Flack parameter	0.072(9)

Table S14: *Crystal data and structure refinement for* ***10R****.*

Empirical formula	$C_{105.5}H_{87.5}Cl_{1.5}EuF_9N_9O_{18}S_3$
Formula weight	2241.65
Temperature/K	100.00
Crystal system	hexagonal
Space group	P63
a/Å	22.9098(5)
b/Å	22.9098(5)
c/Å	12.8924(4)
α/°	90
β/°	90
γ/°	120
Volume/Å3	5860.1(3)
Z	2
ρcalcg/cm3	1.270
μ/mm-1	0.700
F(000)	2290.0
Crystal size/mm3	0.55 × 0.082 × 0.064
Radiation	Mo Kα (λ = 0.71073)
2Θ range for data collection/°	5.182 to 54.26
Index ranges	-29 ≤ h ≤ 29, -29 ≤ k ≤ 29, -16 ≤ l ≤ 16
Reflections collected	168122
Independent reflections	8652 [Rint = 0.0568, Rsigma = 0.0270]
Data/restraints/parameters	8652/1605/528
Goodness-of-fit on F2	1.087
Final R indexes [I>=2σ (I)]	R1 = 0.0613, wR2 = 0.1717
Final R indexes [all data]	R1 = 0.0657, wR2 = 0.1752
Largest diff. peak/hole / e Å-3	0.98/-0.67
Flack parameter	0.080(6)

***Table S15:** Crystal data and structure refinement for **11S**.*

Empirical formula	$C_{111}H_{111}EuF_9N_9O_{24}S_3$	
Formula weight	2374.22	
Temperature	105(2) K	
Wavelength	0.71073 Å	
Crystal system	Hexagonal	
Space group	P 63	
Unit cell dimensions	a = 22.7265(11) Å	α= 90°.
	b = 22.7265(11) Å	β= 90°.
	c = 13.0251(6) Å	γ = 120°.
Volume	5826.1(6) $Å^3$	
Z	2	
Density (calculated)	1.353 Mg/m^3	
Absorption coefficient	0.678 mm^{-1}	
F(000)	2448	
Crystal size	0.105 x 0.11 x 0.489 mm^3	
Theta range for data collection	1.035 to 26.371°.	
Index ranges	-28<=h<=28, -28<=k<=28, -16<=l<=16	
Reflections collected	42836	
Independent reflections	7884 [R(int) = 0.0271]	
Completeness to theta = 25.242°	100.0 %	
Refinement method	Full-matrix least-squares on F^2	
Data / restraints / parameters	7884 / 32 / 473	
Goodness-of-fit on F^2	1.094	
Final R indices [I>2sigma(I)]	R_1 = 0.0482, wR_2 = 0.1244	
R indices (all data)	R_1 = 0.0539, wR_2 = 0.1270	
Absolute structure parameter	-0.017(19)	
Largest diff. peak and hole	1.424 and -0.621 e.$Å^{-3}$	

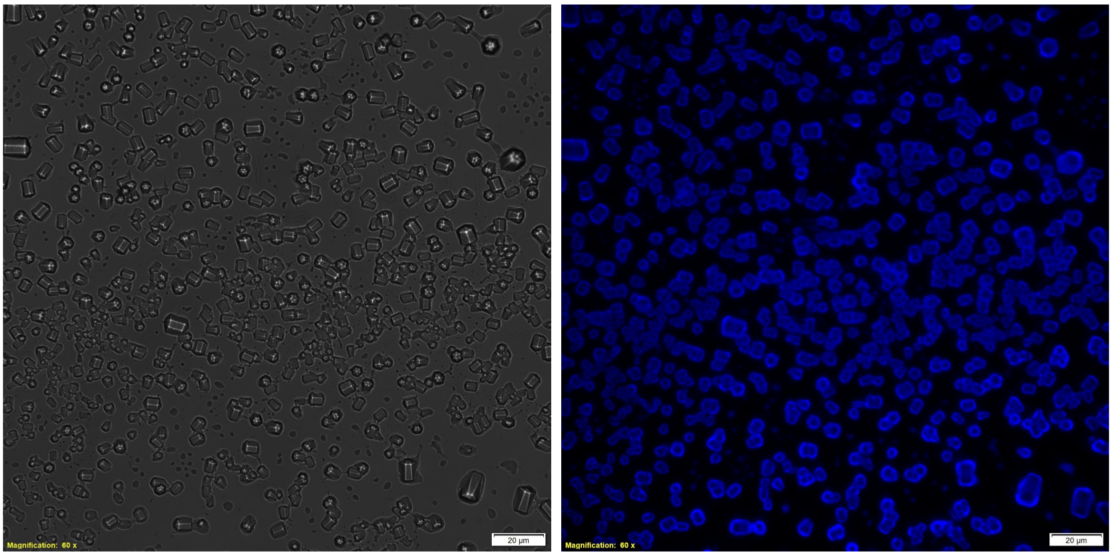

Figure S48: *Confocal images of sample of* ***9S*** *deposited form MeOH.*

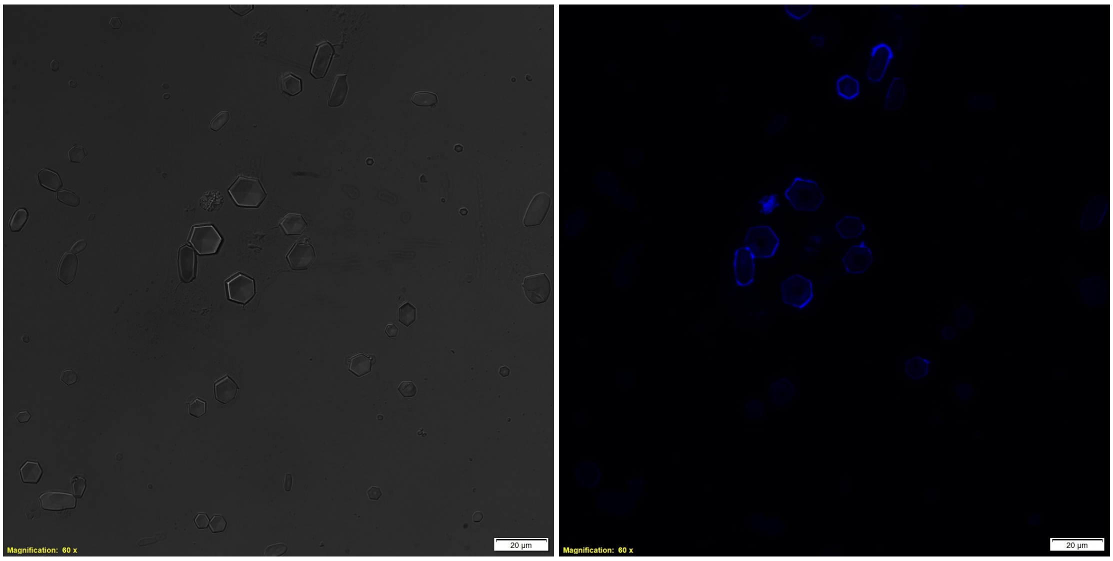

Figure S49: *Confocal images of sample of* ***9S*** *deposited form MeCN.*

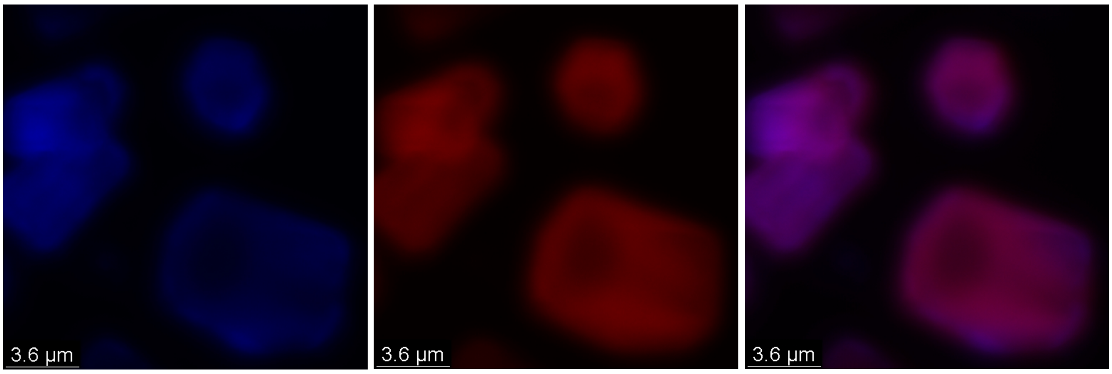

Channel 1: 499 nm-540 nm

Channel 2: 605 nm-620 nm

Overlay image

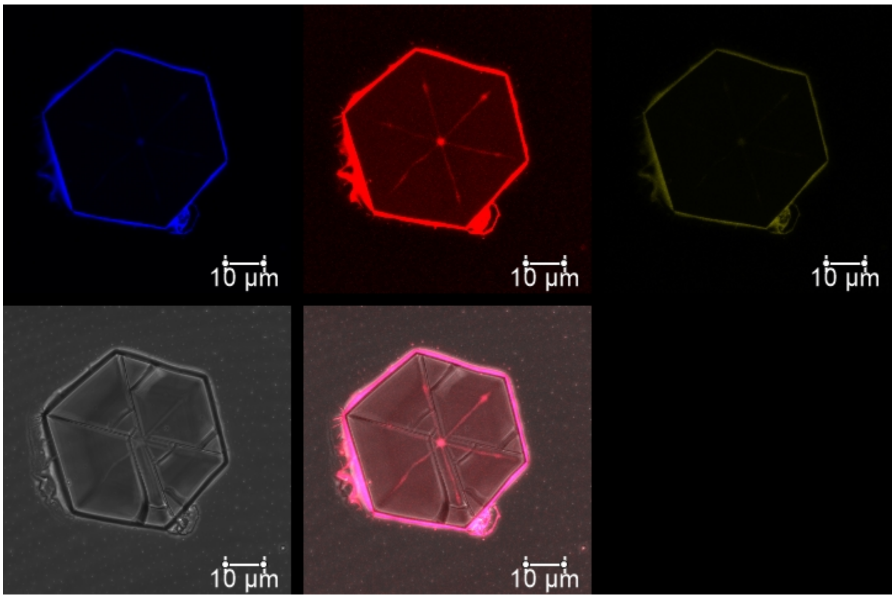

Maximum projection option in leica software show this image

Laser Lines

Laser Line	Intensity
(405 nm)	Shutter: on, Intensity: 5.9999%
(458 nm)	Shutter: off, Intensity: 0.0000%
(476 nm)	Shutter: off, Intensity: 0.0000%

Detectors

Name	Channel	Type	Location	Active	Gain	Offset	Gate Start	Gate End	Gate Ref. Wavelength
PMT 1	Channel 1	PMT (380nm - 385nm)	Internal	Inactive	0	0	-- Time Gating not supported --		
HyD 2	Channel 2	HyD (411nm - 515nm) Standard mode	Internal	Active	37.8	-0.01	0.3 ns	6 ns	-1 nm
PMT 3	Channel 3	PMT (557nm - 650nm)	Internal	Active	960.1	0	-- Time Gating not supported --		
HyD 4	Channel 4	HyD (660nm - 763nm) Standard mode	Internal	Active	69.8	-0.01	-- Time Gating not activated --		
PMT 5	Channel 5	PMT (794nm - 799nm)	Internal	Inactive	0	0	-- Time Gating not supported --		
PMT Trans	Transmission Channel	PMT	TLD	Active	261.2	0	-- Time Gating not supported --		

Figure S50: *Confocal images of sample of a single crystal isolated from* ***9S*** *and the settings employed in their recordings.*

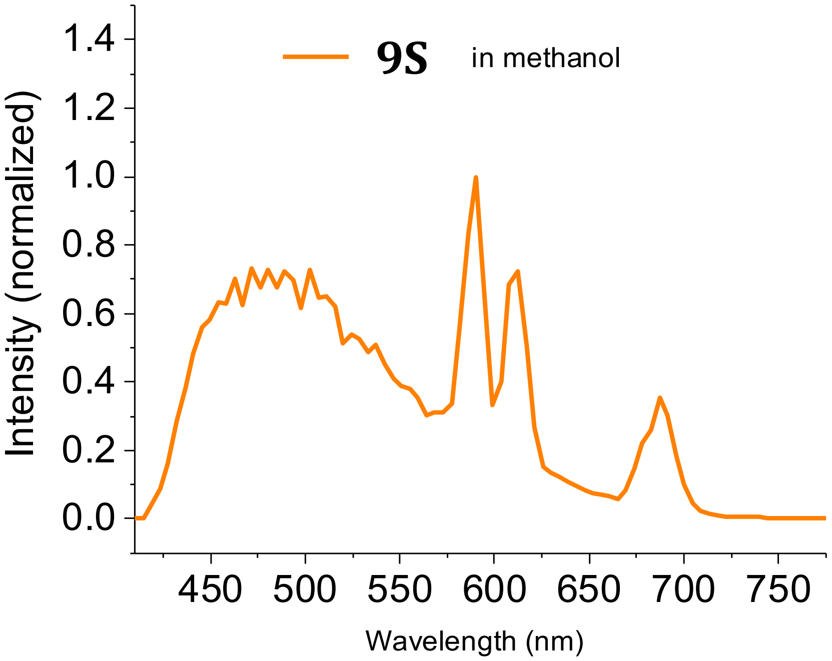

Figure S51: *The Eu(III) emission from crystalline sample of **9S** (deposited from MeOH) recorded in the fluorescence confocal microscope.*

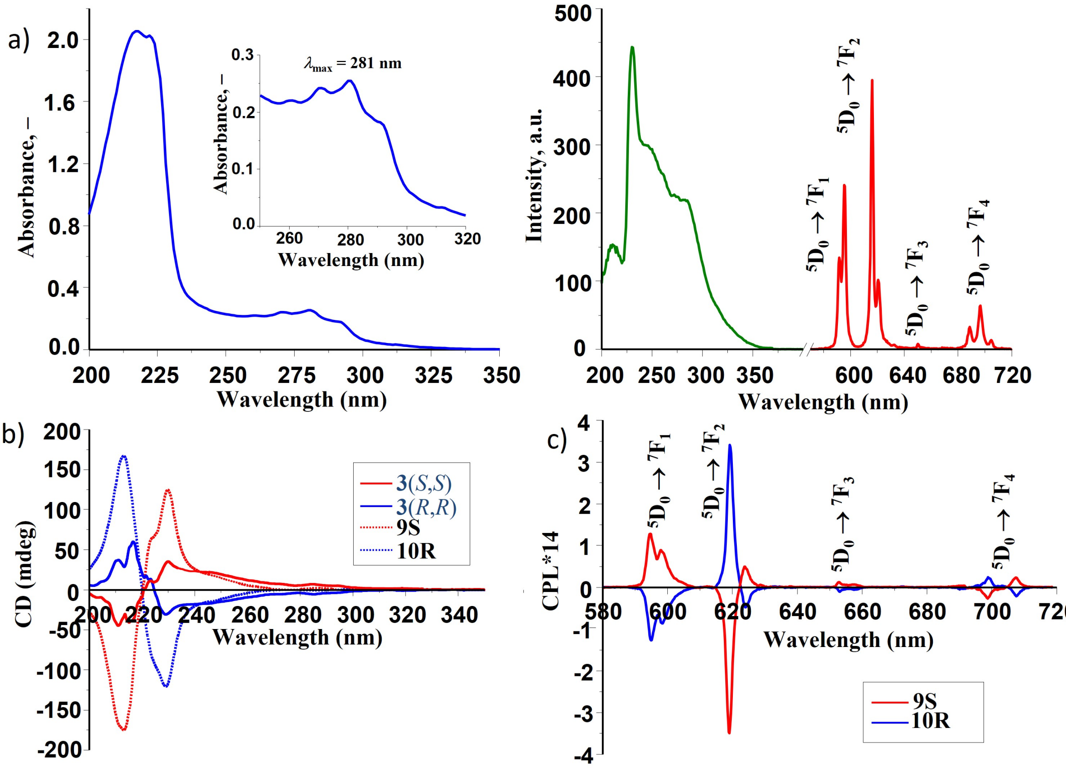

Figure S52: *a) The absorption (blue), phosphorescence excitation (green) and emission (red) spectra (λ_{ex} = 281 nm) of **9S** (c = 6.4 × 10^{-6} M) recorded in MeOH at 298 K. b) CD spectra of ligand solutions of **3(S,S)** and **3(R,R)** (c = 2.0 × 10^{-5} M) recorded in MeCN at 298 K before (solid lines) and after (dashed lines) the addition of 0.33 eq. of $Eu(CF_3SO_3)_3$ to give the complexes **9S** and **10R**, respectively. c) CPL emission spectra of **9S** and **10R** recorded in MeCN at 298 K (λ_{ex} = 281 nm).*

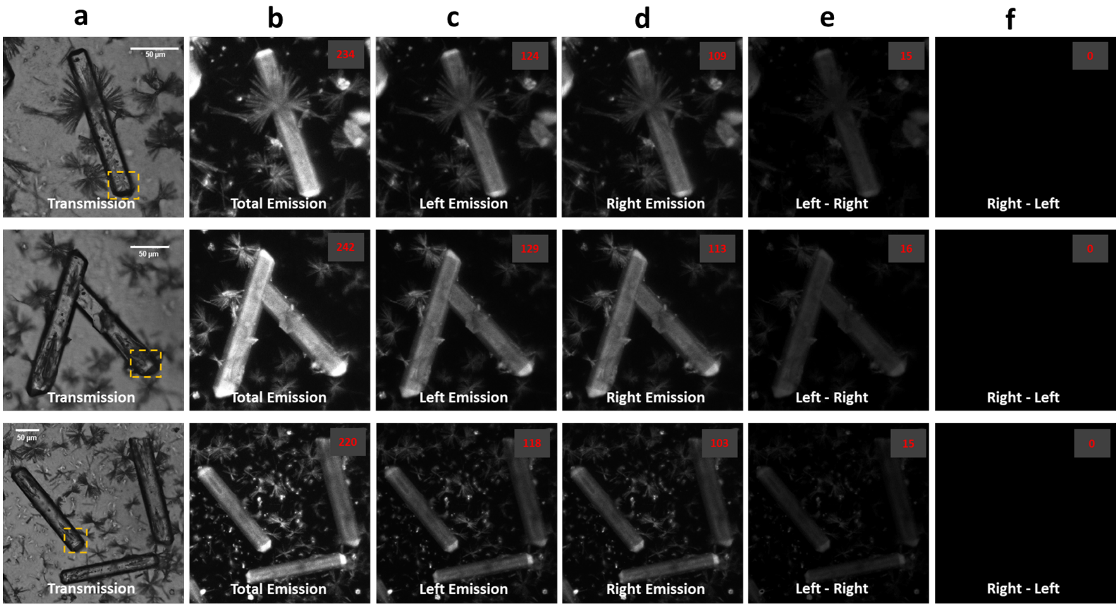

Figure S53: *Enantioselective differential chiral contrast (EDCC) CPL-LSCM of* ***9S*** *on a glass substrate. a) Transmission image, b) Total europium emission (λ_{ex}= 355nm, 20 mW, λ_{em} = 594/10 nm), c,d) Left and Right Handed CPL channel respectively (λ_{em} = 594/10 nm), e) Left-handed EDCC image (left CPL—right CPL) , f) Right-handed EDCC image (right CPL—left CPL). EDCC L-R and R-L have both been contrast enhanced by 40% for better visual representation. The objective used: ×40 0.7 NA air, 210 × 210 µM FOV, 100 AVG, 1.5 µM AXIAL SECTION, the whole dimensions of the crystal have been captured (length x width x depth). Scale bars = 50 µm, numbers in red are avg. Eight-bit pixel intensity values for each image region.*

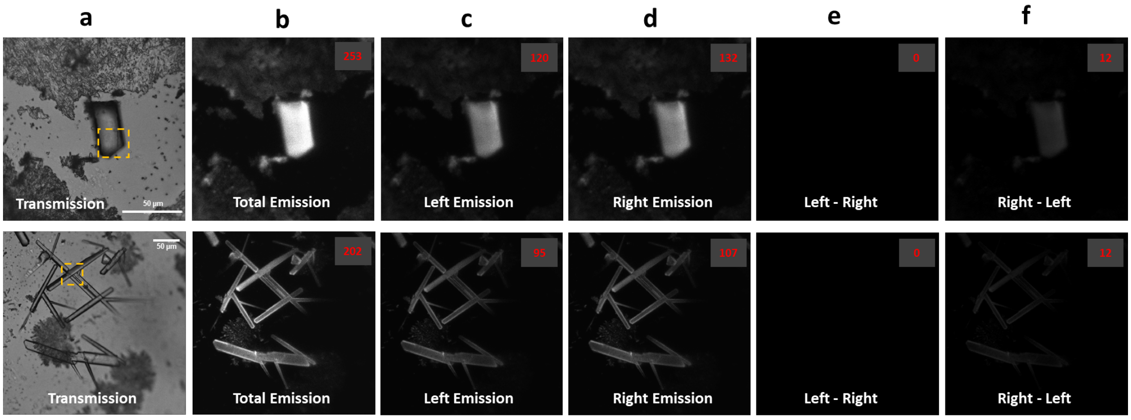

Figure S54: *Enantioselective differential chiral contrast (EDCC) CPL-LSCM of* ***10R*** *on a glass substrate. a) Transmission image, b) Total europium emission (λ_{ex}= 355nm, 20 mW, λ_{em} = 594/10 nm), c,d) Left and Right Handed CPL channel respectively (λ_{em} = 594/10 nm), e) Left-handed EDCC image (left CPL—right CPL) , f) Right-handed EDCC image (right CPL—left CPL). EDCC L-R and R-L have both been contrast enhanced by 40% for better visual representation. The objective used: ×40 0.7 NA air, 210 × 210 µM FOV, 100 AVG, 1.5 µM AXIAL SECTION, the whole dimensions of the crystal have been captured (length x width x depth). Scale bars = 50 µm, numbers in red are avg. Eight-bit pixel intensity values for each image region.*

References

1. Vogel, A. I., Tatchell, A. R., Furnis, B. S., Hannaford, A. J., Smith, P. W. G. *Vogel's Textbook of Practical Organic Chemistry*; 5th ed.; Prentice Hall: New York, **1996**.
2. Chauvin, A.-S., Gumy, F., Imbert, D., Bünzli, J.-C. G. *Spectrosc. Lett.* **2004**, *37*, 517.
3. Chauvin, A.-S., Gumy, F., Imbert, D., Bünzli, J.-C. G. *Spectrosc. Lett.* **2007**, *40*, 193.
4. Sheldrick, G. *Acta Crystallogr., Sect. A* **2008**, *64*, 112.
5. Carr, R., Puckrin, R., McMahon, B.K., Pal, R., Parker, D. & Palsson, L-O., Induced circularly polarized luminescence arising from anion or protein binding to racemic emissive lanthanide complexes, *Methods Appl. Fluoresc*. **2**, 024007 (2014).
6. MacKenzie, L. E., Pålsson, L.-O., Parker, D., Beeby, A. & Pal, R. Rapid time-resolved Circular Polarization Luminescence (CPL) emission spectroscopy. *Nat. Commun.* **11**, 1676 (2020)
7. Stachelek, P.; MacKenzie, L.; Parker, D.; Pal, R. Circularly Polarised Luminescence Laser Scanning Confocal Microscopy to Study Live Cell Chiral Molecular Interactions. *Nat. Commun.* **2022**, *13* (1), 553.
8. Pal, R. Phase Modulation Nanoscopy: A Simple Approach to Enhanced Optical Resolution. *Faraday Discuss.* **2015**, *177* (0), 507.
