# Supplementary material for: Ligand Chirality Transfer from Solution State to the Crystalline Self‐Assemblies in Circularly Polarized Luminescence (CPL) Active Lanthanide Systems
Source: Adv Sci (Weinh). 2024 Mar 6;11(18):2307448. doi: 10.1002/advs.202307448 (PMC11095229; doi:10.1002/advs.202307448)

checkCIF/PLATON report

Structure factors have been supplied for datablock(s) DB073

THIS REPORT IS FOR GUIDANCE ONLY. IF USED AS PART OF A REVIEW PROCEDURE FOR PUBLICATION, IT SHOULD NOT REPLACE THE EXPERTISE OF AN EXPERIENCED CRYSTALLOGRAPHIC REFEREE.

No syntax errors found. CIF dictionary Interpreting this report

Datablock: DB073

```
Bond precision:   C-C = 0.0074 A          Wavelength=0.71073

Cell:               a=14.906(5)       b=19.311(5)       c=23.190(5)
                    alpha=90          beta=90           gamma=90
Temperature:        102 K

                Calculated                     Reported
Volume          6675(3)                        6675(3)
Space group     P 21 21 21                     P 21 21 21
Hall group      P 2ac 2ab                      P 2ac 2ab
Moiety formula  C36 H35 N3 O5, C2 H6 O         C74 H72 N4 O14
Sum formula     C38 H41 N3 O6                  C76 H82 N6 O12
Mr              635.74                         1271.47
Dx,g cm-3       1.265                          1.265
Z               8                              4
Mu (mm-1)       0.086                          0.086
F000            2704.0                         2704.0
F000'           2705.24
h,k,lmax        19,25,30                       19,25,30
Nref            15913[ 8674]                   73933
Tmin,Tmax       0.982,0.991
Tmin'           0.977

Correction method= Not given

Data completeness= 8.52/4.65       Theta(max)= 27.861

                                                       wR2(reflections)=
R(reflections)= 0.0681( 62615)                         0.2015( 73933)

S = 1.005                  Npar= 847
```

The following ALERTS were generated. Each ALERT has the format
test-name_ALERT_alert-type_alert-level.
Click on the hyperlinks for more details of the test.

Alert level A

```
SHFSU01_ALERT_2_A  The absolute value of parameter shift to su ratio > 0.20
            Absolute value of the parameter shift to su ratio given   8.056
            Additional refinement cycles may be required.
PLAT080_ALERT_2_A Maximum Shift/Error ...........................       8.06 Why ?
PLAT213_ALERT_2_A Atom C092            has ADP max/min Ratio .....       7.0 prolat
PLAT242_ALERT_2_A Low    'MainMol' Ueq as Compared to Neighbors of        O6 Check
PLAT375_ALERT_2_A Strange C-O-H Geometry (C-O  >   1.45 Ang) .....       O10 Check
```

Alert level B

```
PLAT021_ALERT_4_B Ratio Unique / Expected Reflections too High ...      8.524
PLAT097_ALERT_2_B Large Reported Max.  (Positive) Residual Density       1.12 eA-3
PLAT196_ALERT_1_B No TEMP record and _measurement_temperature .NE.        293 Degree
PLAT220_ALERT_2_B NonSolvent   Resd 2  C   Ueq(max)/Ueq(min) Range       10.0 Ratio
PLAT220_ALERT_2_B NonSolvent   Resd 2  O   Ueq(max)/Ueq(min) Range        7.2 Ratio
PLAT230_ALERT_2_B Hirshfeld Test Diff for    O5        --C086     .      13.3 s.u.
PLAT230_ALERT_2_B Hirshfeld Test Diff for    O6        --C12      .       8.1 s.u.
PLAT230_ALERT_2_B Hirshfeld Test Diff for    O6        --C093     .       7.9 s.u.
PLAT230_ALERT_2_B Hirshfeld Test Diff for    C092      --C093     .      11.0 s.u.
PLAT241_ALERT_2_B High   'MainMol' Ueq as Compared to Neighbors of       C092 Check
PLAT992_ALERT_5_B Repd & Actual _reflns_number_gt Values Differ by         96 Check
```

Alert level C

```
DIFMN02_ALERT_2_C  The minimum difference density is < -0.1*ZMAX*0.75
            _refine_diff_density_min given =     -0.686
            Test value =     -0.600
DIFMN03_ALERT_1_C  The minimum difference density is < -0.1*ZMAX*0.75
            The relevant atom site should be identified.
DIFMX02_ALERT_1_C  The maximum difference density is > 0.1*ZMAX*0.75
            The relevant atom site should be identified.
PLAT042_ALERT_1_C Calc. and Reported MoietyFormula Strings  Differ     Please Check
PLAT098_ALERT_2_C Large Reported Min.  (Negative) Residual Density      -0.69 eA-3
PLAT147_ALERT_1_C s.u. on Symmetry Constrained Cell Angle(s) .....     Please Check
PLAT213_ALERT_2_C Atom O6              has ADP max/min Ratio .....        3.7 prolat
PLAT213_ALERT_2_C Atom C12             has ADP max/min Ratio .....        3.3 prolat
PLAT213_ALERT_2_C Atom C086            has ADP max/min Ratio .....        3.3 oblate
PLAT213_ALERT_2_C Atom C093            has ADP max/min Ratio .....        3.6 prolat
PLAT222_ALERT_3_C NonSolvent Resd 2  H   Uiso(max)/Uiso(min) Range       10.0 Ratio
PLAT242_ALERT_2_C Low    'MainMol' Ueq as Compared to Neighbors of         O5 Check
PLAT242_ALERT_2_C Low    'MainMol' Ueq as Compared to Neighbors of       C093 Check
PLAT244_ALERT_4_C Low    'Solvent' Ueq as Compared to Neighbors of         C7 Check
PLAT244_ALERT_4_C Low    'Solvent' Ueq as Compared to Neighbors of         C9 Check
PLAT260_ALERT_2_C Large Average Ueq of Residue Including       O10      0.128 Check
PLAT260_ALERT_2_C Large Average Ueq of Residue Including       O11      0.110 Check
PLAT340_ALERT_3_C Low Bond Precision on  C-C Bonds ...............    0.00743 Ang.
PLAT360_ALERT_2_C Short  C(sp3)-C(sp3) Bond  C092      - C093     .       1.41 Ang.
PLAT410_ALERT_2_C Short Intra H...H Contact  H034      ..H068     .       1.96 Ang.
                                                        x,y,z  =      1_555 Check
```

```
PLAT410_ALERT_2_C Short Intra H...H Contact  H021     ..H077      .       1.98 Ang.
                                                       x,y,z  =      1_555 Check
PLAT761_ALERT_1_C CIF Contains no X-H Bonds ......................     Please Check
PLAT762_ALERT_1_C CIF Contains no X-Y-H or H-Y-H Angles ..........     Please Check
PLAT790_ALERT_4_C Centre of Gravity not Within Unit Cell: Resd.  #          1 Note
              C36 H35 N3 O5
PLAT911_ALERT_3_C Missing FCF Refl Between Thmin & STh/L=    0.600          7 Report
PLAT918_ALERT_3_C Reflection(s) with I(obs) much Smaller I(calc) .         11 Check
PLAT975_ALERT_2_C Check Calcd Resid. Dens.  0.89Ang From N1      .       0.47 eA-3
PLAT975_ALERT_2_C Check Calcd Resid. Dens.  0.90Ang From N3      .       0.45 eA-3
PLAT975_ALERT_2_C Check Calcd Resid. Dens.  0.99Ang From N6      .       0.44 eA-3
PLAT976_ALERT_2_C Check Calcd Resid. Dens.  0.63Ang From O10     .      -0.63 eA-3
PLAT976_ALERT_2_C Check Calcd Resid. Dens.  0.75Ang From O10     .      -0.57 eA-3
PLAT976_ALERT_2_C Check Calcd Resid. Dens.  0.56Ang From O11     .      -0.41 eA-3
PLAT976_ALERT_2_C Check Calcd Resid. Dens.  0.53Ang From O11     .      -0.41 eA-3
```

Alert level G

```
FORMU01_ALERT_1_G  There is a discrepancy between the atom counts in the
            _chemical_formula_sum and _chemical_formula_moiety. This is
            usually due to the moiety formula being in the wrong format.
            Atom count from _chemical_formula_sum:   C76 H82 N6 O12
            Atom count from _chemical_formula_moiety:C74 H72 N4 O14
PLAT002_ALERT_2_G Number of Distance or Angle Restraints on AtSite          2 Note
PLAT003_ALERT_2_G Number of Uiso or Uij Restrained non-H Atoms ...          1 Report
PLAT007_ALERT_5_G Number of Unrefined Donor-H Atoms .............          2 Report
PLAT045_ALERT_1_G Calculated and Reported Z Differ by a Factor ...          2 Check
PLAT072_ALERT_2_G SHELXL First  Parameter in WGHT  Unusually Large       0.12 Report
PLAT083_ALERT_2_G SHELXL Second Parameter in WGHT  Unusually Large       7.30 Why ?
PLAT153_ALERT_1_G The s.u.'s on the Cell Axes   are Equal ..(Note)      0.005 Ang.
PLAT172_ALERT_4_G The CIF-Embedded .res File Contains DFIX Records          1 Report
PLAT186_ALERT_4_G The CIF-Embedded .res File Contains ISOR Records          1 Report
PLAT432_ALERT_2_G Short Inter X...Y Contact  O007     ..C079      .       2.98 Ang.
                                          1/2+x,-1/2-y,1-z  =      3_546 Check
PLAT720_ALERT_4_G Number of Unusual/Non-Standard Labels ..........        135 Note
PLAT790_ALERT_4_G Centre of Gravity not Within Unit Cell: Resd.  #          3 Note
              C2 H6 O
PLAT791_ALERT_4_G Model has Chirality at C021        (Sohnke SpGr)          R Verify
PLAT791_ALERT_4_G Model has Chirality at C3          (Sohnke SpGr)          R Verify
PLAT791_ALERT_4_G Model has Chirality at C028        (Sohnke SpGr)          R Verify
PLAT791_ALERT_4_G Model has Chirality at C034        (Sohnke SpGr)          R Verify
PLAT802_ALERT_4_G CIF Input Record(s) with more than 80 Characters          1 Info
PLAT860_ALERT_3_G Number of Least-Squares Restraints ............          7 Note
PLAT883_ALERT_1_G No Info/Value for _atom_sites_solution_primary .     Please Do !
PLAT912_ALERT_4_G Missing # of FCF Reflections Above STh/L=  0.600        141 Note
PLAT933_ALERT_2_G Number of HKL-OMIT Records in Embedded .res File         12 Note
PLAT978_ALERT_2_G Number C-C Bonds with Positive Residual Density.          0 Info
PLAT996_ALERT_1_G Non-Standard SHELXL LIST 4 Style FCF Supplied ..          ! Check
```

---

5 **ALERT level A** = Most likely a serious problem - resolve or explain
11 **ALERT level B** = A potentially serious problem, consider carefully
33 **ALERT level C** = Check. Ensure it is not caused by an omission or oversight
24 **ALERT level G** = General information/check it is not something unexpected

12 ALERT type 1 CIF construction/syntax error, inconsistent or missing data

```
 40 ALERT type 2 Indicator that the structure model may be wrong or deficient
  5 ALERT type 3 Indicator that the structure quality may be low
 14 ALERT type 4 Improvement, methodology, query or suggestion
  2 ALERT type 5 Informative message, check
```

checkCIF publication errors

Alert level A

```
PUBL006_ALERT_1_A  _publ_requested_journal is missing
            e.g. 'Acta Crystallographica Section C'
PUBL008_ALERT_1_A  _publ_section_title is missing. Title of paper.
PUBL009_ALERT_1_A  _publ_author_name is missing. List of author(s) name(s).
PUBL010_ALERT_1_A  _publ_author_address is missing. Author(s) address(es).
PUBL012_ALERT_1_A  _publ_section_abstract is missing.
            Abstract of paper in English.
```

```
  5 ALERT level A = Data missing that is essential or data in wrong format
  0 ALERT level G = General alerts. Data that may be required is missing
```

Publication of your CIF

You should attempt to resolve as many as possible of the alerts in all categories. Often the minor alerts point to easily fixed oversights, errors and omissions in your CIF or refinement strategy, so attention to these fine details can be worthwhile. In order to resolve some of the more serious problems it may be necessary to carry out additional measurements or structure refinements. However, the nature of your study may justify the reported deviations from journal submission requirements and the more serious of these should be commented upon in the discussion or experimental section of a paper or in the "special_details" fields of the CIF. *checkCIF* was carefully designed to identify outliers and unusual parameters, but every test has its limitations and alerts that are not important in a particular case may appear. Conversely, the absence of alerts does not guarantee there are no aspects of the results needing attention. It is up to the individual to critically assess their own results and, if necessary, seek expert advice.

If level A alerts remain, which you believe to be justified deviations, and you intend to submit this CIF for publication in a journal, you should additionally insert an explanation in your CIF using the Validation Reply Form (VRF) below. This will allow your explanation to be considered as part of the review process.

```
# start Validation Reply Form
_vrf_PUBL006_GLOBAL
;
PROBLEM: _publ_requested_journal is missing
RESPONSE: ...
;
_vrf_PUBL008_GLOBAL
;
```

```
PROBLEM: _publ_section_title is missing. Title of paper.
RESPONSE: ...
;
_vrf_PUBL009_GLOBAL
;
PROBLEM: _publ_author_name is missing. List of author(s) name(s).
RESPONSE: ...
;
_vrf_PUBL010_GLOBAL
;
PROBLEM: _publ_author_address is missing. Author(s) address(es).
RESPONSE: ...
;
_vrf_PUBL012_GLOBAL
;
PROBLEM: _publ_section_abstract is missing.
RESPONSE: ...
;
_vrf_SHFSU01_DB073
;
PROBLEM: The absolute value of parameter shift to su ratio > 0.20
RESPONSE: ...
;
_vrf_PLAT080_DB073
;
PROBLEM: Maximum Shift/Error ............................       8.06 Why ?
RESPONSE: ...
;
_vrf_PLAT213_DB073
;
PROBLEM: Atom C092            has ADP max/min Ratio .....        7.0 prolat
RESPONSE: ...
;
_vrf_PLAT242_DB073
;
PROBLEM: Low    'MainMol' Ueq as Compared to Neighbors of         O6 Check
RESPONSE: ...
;
_vrf_PLAT375_DB073
;
PROBLEM: Strange C-O-H Geometry (C-O  >   1.45 Ang) .....        O10 Check
RESPONSE: ...
;
# end Validation Reply Form
```

If you wish to submit your CIF for publication in Acta Crystallographica Section C or E, you should upload your CIF via the web. If you wish to submit your CIF for publication in IUCrData you should upload your CIF via the web. If your CIF is to form part of a submission to another IUCr journal, you will be asked, either during electronic submission or by the Co-editor handling your paper, to upload your CIF via our web site.

PLATON version of 12/09/2022; check.def file version of 09/08/2022

Datablock DB073 - ellipsoid plot

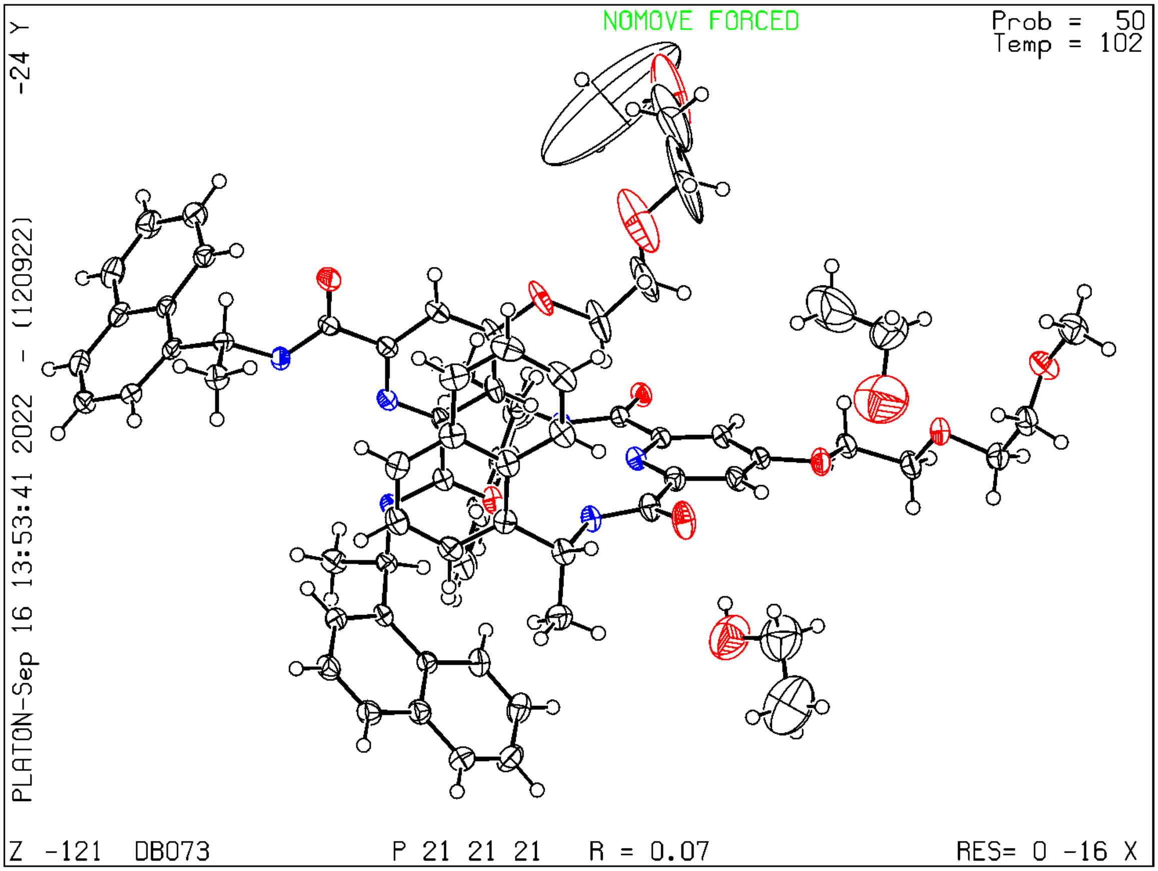

Supplement: Supplementary file 2 — Supporting Information [file ADVS-11-2307448-s001.zip › advs202307448-sup-0002-SuppMat.zip/checkCIF_Angew. Chemie. 1.pdf]
