# Supplementary material for: Ligand Chirality Transfer from Solution State to the Crystalline Self‐Assemblies in Circularly Polarized Luminescence (CPL) Active Lanthanide Systems
Source: Adv Sci (Weinh). 2024 Mar 6;11(18):2307448. doi: 10.1002/advs.202307448 (PMC11095229; doi:10.1002/advs.202307448)
Supplement: Supplementary file 2 — Supporting Information [file ADVS-11-2307448-s001.zip › advs202307448-sup-0002-SuppMat.zip/checkCIF_Angew. Chemie. 2.pdf]

No syntax errors found.
Please wait while processing ....

CIF dictionary
Interpreting this report

# Datablock: DFC029sq

| | | | |
|---|---|---|---|
| Bond precision: | C-C = 0.0214 A | | Wavelength=0.71073 |
| Cell: | a=22.7265(11) | b=22.7265(11) | c=13.0251(6) |
| | alpha=90 | beta=90 | gamma=120 |
| Temperature: | 105 K | | |

| | Calculated | Reported |
|---|---|---|
| Volume | 5826.1(6) | 5826.1(6) |
| Space group | P 63 | P 63 |
| Hall group | P 6c | P 6c |
| Moiety formula | C108 H111 Eu N9 O15, 3(C F3 O3 S) [+ solvent] | C111 H111 N9 O24 F9 S3 Eu1 |
| Sum formula | C111 H111 Eu F9 N9 O24 S3 [+ solvent] | C111 H111 Eu F9 N9 O24 S3 |
| Mr | 2374.24 | 2374.22 |
| Dx,g cm-3 | 1.353 | 1.353 |
| Z | 2 | 2 |
| Mu (mm-1) | 0.678 | 0.678 |
| F000 | 2448.0 | 2448.0 |
| F000' | 2449.73 | |
| h,k,lmax | 28,28,16 | 28,28,16 |
| Nref | 7956[ 4162] | 7884 |
| Tmin,Tmax | 0.914,0.931 | |
| Tmin' | 0.718 | |

Correction method= Not given

Data completeness= 1.89/0.99 Theta(max)= 26.371

R(reflections)= 0.0479( 7137) wR2(reflections)= 0.1275( 7884)

S = 0.957 Npar= 473

The following ALERTS were generated. Each ALERT has the format
**test-name_ALERT_alert-type_alert-level.**
Click on the hyperlinks for more details of the test.

## Alert level A

```
PLAT234_ALERT_4_A Large Hirshfeld Difference C37      --C38      .       0.36 Ang.
PLAT411_ALERT_2_A Short Inter H...H Contact  H45B     ..H45B     .       1.73 Ang.
                                          -y,x-y,z  =      4_555 Check
PLAT411_ALERT_2_A Short Inter H...H Contact  H45B     ..H45B     .       1.73 Ang.
                                         -x+y,-x,z  =      5_555 Check
```

## Alert level B

```
PLAT213_ALERT_2_B Atom C44             has ADP max/min Ratio .....        4.5 prolat
PLAT220_ALERT_2_B NonSolvent   Resd 1  C   Ueq(max)/Ueq(min) Range       10.0 Ratio
PLAT220_ALERT_2_B NonSolvent   Resd 1  O   Ueq(max)/Ueq(min) Range       10.0 Ratio
PLAT230_ALERT_2_B Hirshfeld Test Diff for    O4        --C43      .        8.7 s.u.
PLAT234_ALERT_4_B Large Hirshfeld Difference C14       --C43      .       0.30 Ang.
PLAT234_ALERT_4_B Large Hirshfeld Difference F3        --C50      .       0.26 Ang.
PLAT241_ALERT_2_B High   'MainMol' Ueq as Compared to Neighbors of         O5 Check
PLAT241_ALERT_2_B High   'MainMol' Ueq as Compared to Neighbors of        C45 Check
PLAT242_ALERT_2_B Low    'MainMol' Ueq as Compared to Neighbors of         O4 Check
PLAT242_ALERT_2_B Low    'MainMol' Ueq as Compared to Neighbors of        C14 Check
PLAT342_ALERT_3_B Low Bond Precision on  C-C Bonds ..............    0.02138 Ang.
PLAT990_ALERT_1_B Deprecated .res/.hkl Input Style SQUEEZE Job ...          ! Note
```

## Alert level C

```
PLAT094_ALERT_2_C Ratio of Maximum / Minimum Residual Density ....       2.22 Report
PLAT213_ALERT_2_C Atom O4              has ADP max/min Ratio .....        3.2 prolat
PLAT213_ALERT_2_C Atom C46             has ADP max/min Ratio .....        3.3 prolat
PLAT222_ALERT_3_C NonSolvent Resd 1  H   Uiso(max)/Uiso(min) Range       10.0 Ratio
PLAT234_ALERT_4_C Large Hirshfeld Difference C25       --C26      .       0.19 Ang.
PLAT234_ALERT_4_C Large Hirshfeld Difference S1        --C50      .       0.23 Ang.
PLAT241_ALERT_2_C High   'MainMol' Ueq as Compared to Neighbors of         C7 Check
```

**And 4 other PLAT241 Alerts**
More ...

```
PLAT242_ALERT_2_C Low    'MainMol' Ueq as Compared to Neighbors of         C6 Check
PLAT244_ALERT_4_C Low    'Solvent' Ueq as Compared to Neighbors of         S1 Check
PLAT250_ALERT_2_C Large U3/U1 Ratio for Average U(i,j) Tensor ....        2.2 Note
```

```
PLAT250_ALERT_2_C Large U3/U1 Ratio for Average U(i,j) Tensor ....        3.4 Note
PLAT260_ALERT_2_C Large Average Ueq of Residue Including       Eu1      0.135 Check
PLAT260_ALERT_2_C Large Average Ueq of Residue Including        S1      0.156 Check
PLAT410_ALERT_2_C Short Intra H...H Contact  H3        ..H36        .    1.95 Ang.
                                                  x,y,z  =      1_555 Check
PLAT431_ALERT_2_C Short Inter HL..A Contact  F2        ..O3         .    2.88 Ang.
                                            1-y,1+x-y,z  =      4_665 Check
```

## Alert level G

```
PLAT002_ALERT_2_G Number of Distance or Angle Restraints on AtSite          2 Note
PLAT003_ALERT_2_G Number of Uiso or Uij Restrained non-H Atoms ...          5 Report
PLAT007_ALERT_5_G Number of Unrefined Donor-H Atoms .............          2 Report
PLAT042_ALERT_1_G Calc. and Reported MoietyFormula Strings  Differ     Please Check
PLAT172_ALERT_4_G The CIF-Embedded .res File Contains DFIX Records          1 Report
PLAT178_ALERT_4_G The CIF-Embedded .res File Contains SIMU Records          1 Report
PLAT244_ALERT_4_G Low    'Solvent' Ueq as Compared to Neighbors of        C50 Check
PLAT335_ALERT_2_G Check Large C6 Ring C-C Range C4       -C9            0.16 Ang.
PLAT343_ALERT_2_G Unusual sp3      Angle Range in Main Residue for        C44 Check
PLAT398_ALERT_2_G Deviating  C-O-C    Angle From 120 for O5        .     148.5 Degree
PLAT605_ALERT_4_G Largest Solvent Accessible VOID in the Structure        165 A**3
PLAT791_ALERT_4_G Model has Chirality at C3           (Sohnke SpGr)         S Verify
PLAT791_ALERT_4_G Model has Chirality at C21          (Sohnke SpGr)         S Verify
PLAT802_ALERT_4_G CIF Input Record(s) with more than 80 Characters          1 Info
PLAT860_ALERT_3_G Number of Least-Squares Restraints ............          20 Note
PLAT869_ALERT_4_G ALERTS Related to the Use of SQUEEZE  Suppressed          ! Info
PLAT883_ALERT_1_G No Info/Value for _atom_sites_solution_primary .     Please Do !
PLAT912_ALERT_4_G Missing # of FCF Reflections Above STh/L=  0.600          3 Note
PLAT961_ALERT_5_G Dataset Contains no Negative Intensities .......     Please Check
PLAT978_ALERT_2_G Number C-C Bonds with Positive Residual Density.          0 Info
```

```
  3 ALERT level A = Most likely a serious problem - resolve or explain
 12 ALERT level B = A potentially serious problem, consider carefully
 19 ALERT level C = Check. Ensure it is not caused by an omission or oversight
 20 ALERT level G = General information/check it is not something unexpected

  3 ALERT type 1 CIF construction/syntax error, inconsistent or missing data
 31 ALERT type 2 Indicator that the structure model may be wrong or deficient
  3 ALERT type 3 Indicator that the structure quality may be low
 15 ALERT type 4 Improvement, methodology, query or suggestion
  2 ALERT type 5 Informative message, check
```

It is advisable to attempt to resolve as many as possible of the alerts in all categories. Often the minor alerts point to easily fixed oversights, errors and omissions in your CIF or refinement strategy, so attention to these fine details can be worthwhile. In order to resolve some of the more serious problems it may be necessary to carry out additional measurements or structure refinements. However, the purpose of your study may justify the reported deviations and the more serious of these should normally be commented upon in the discussion or experimental section of a paper or in the "special_details" fields of the CIF. checkCIF was carefully designed to identify outliers and unusual parameters, but every test has its limitations and alerts that are not important in a particular case may appear. Conversely, the absence of alerts does not guarantee there are no aspects of the results needing attention. It is up to the individual to critically assess their own results and, if necessary, seek expert advice.

**Publication of your CIF in IUCr journals**

A basic structural check has been run on your CIF. These basic checks will be run on all CIFs submitted for publication in IUCr journals (*Acta Crystallographica*, *Journal of Applied Crystallography*, *Journal of Synchrotron Radiation*); however, if you intend to submit to *Acta Crystallographica Section C* or *E* or *IUCrData*, you should make sure that full publication checks are run on the final version of your CIF prior to submission.

**Publication of your CIF in other journals**

Please refer to the *Notes for Authors* of the relevant journal for any special instructions relating to CIF submission.

**PLATON version of 18/05/2022; check.def file version of 17/05/2022**

# Datablock DFC029sq - ellipsoid plot

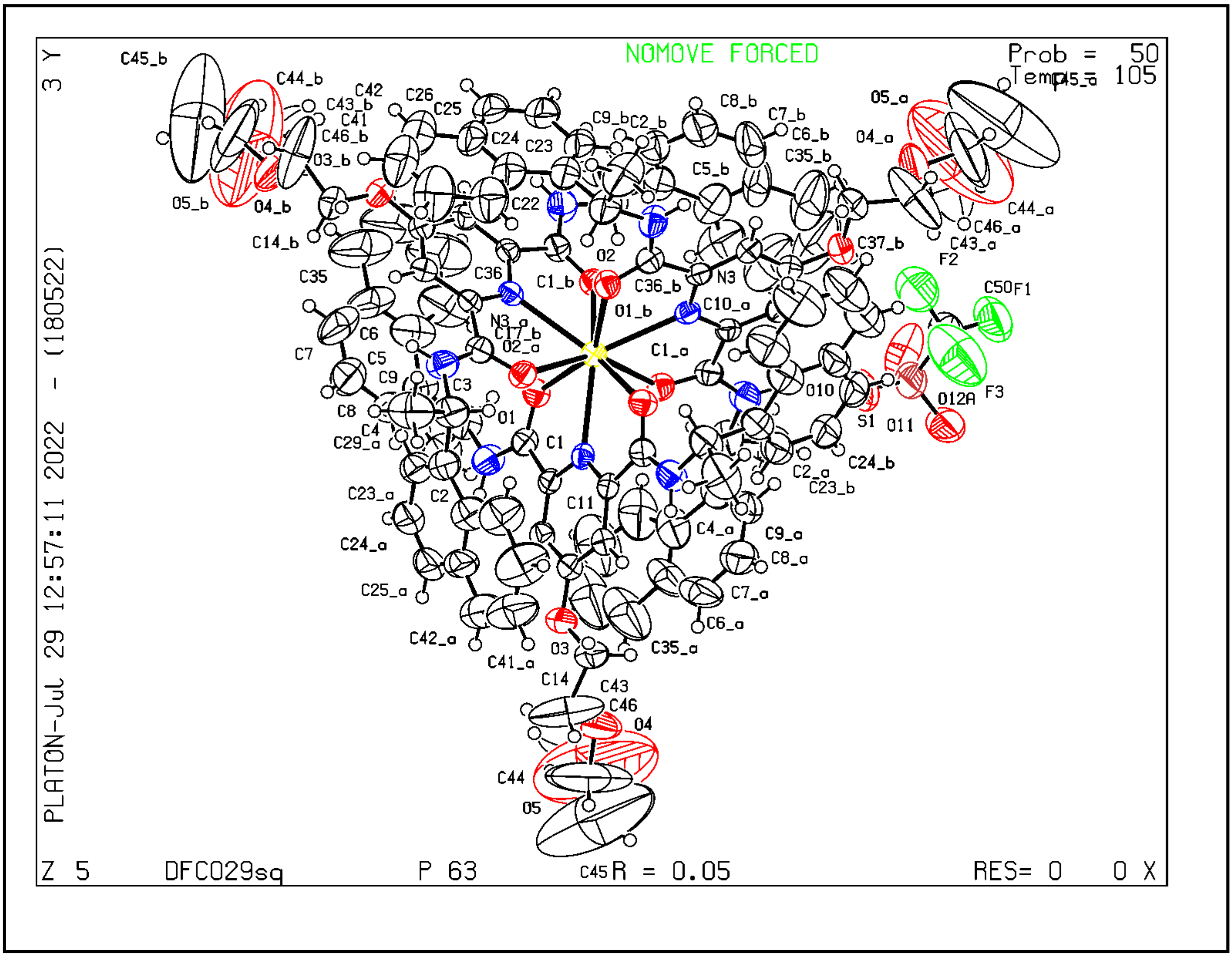


[Download CIF editor (publCIF) from the IUCr](#)
[Download CIF editor (enCIFer) from the CCDC](#)
[Test a new CIF entry](#)
