# Supplementary material for: Ligand Chirality Transfer from Solution State to the Crystalline Self‐Assemblies in Circularly Polarized Luminescence (CPL) Active Lanthanide Systems
Source: Adv Sci (Weinh). 2024 Mar 6;11(18):2307448. doi: 10.1002/advs.202307448 (PMC11095229; doi:10.1002/advs.202307448)
Supplement: Supplementary file 2 — Supporting Information [file ADVS-11-2307448-s001.zip › advs202307448-sup-0002-SuppMat.zip/checkCIF_Angew. Chemie. 4.pdf]

No syntax errors found.
Please wait while processing ....

CIF dictionary
Interpreting this report

Datablock: DFC080sq

Bond precision:	C-C = 0.0137 A		Wavelength=0.71073
Cell:	a=22.977(4)	b=22.977(4)	c=12.936(2)
	alpha=90	beta=90	gamma=120
Temperature:	100 K		

	Calculated	Reported
Volume	5915(2)	5915(2)
Space group	P 63 2 2	P 63 2 2
Hall group	P 6c 2c	P 6c 2c
Moiety formula	C93 H81 Eu N9 O9, 3(Cl O4) [+ solvent]	C93 H81 N9 O27 Cl3 Eu1
Sum formula	C93 H81 Cl3 Eu N9 O21 [+ solvent]	C93 H81 Cl3 Eu N9 O21
Mr	1918.99	1918.97
Dx,g cm-3	1.077	1.078
Z	2	2
Mu (mm-1)	0.656	0.656
F000	1968.0	1968.0
F000'	1969.43	
h,k,lmax	28,28,16	27,28,16
Nref	4063[2370]	83846
Tmin,Tmax	0.868,0.906	
Tmin'	0.843	

Correction method= Not given

Data completeness= ****/****   Theta(max)= 26.379

R(reflections)= 0.0636(72422)   wR2(reflections)= 0.2037(83846)

S = 0.869   Npar= 170

The following ALERTS were generated. Each ALERT has the format
test-name_ALERT_alert-type_alert-level.
Click on the hyperlinks for more details of the test.

Alert level A

```
PLAT234_ALERT_4_A Large Hirshfeld Difference C13      --C14      .        0.32 Ang.
```

Alert level B

```
PLAT021_ALERT_4_B Ratio Unique / Expected Reflections too High ...     35.378
PLAT230_ALERT_2_B Hirshfeld Test Diff for    C10      --C11      .        7.4 s.u.
PLAT234_ALERT_4_B Large Hirshfeld Difference C12      --C15      .       0.30 Ang.
PLAT241_ALERT_2_B High   'MainMol' Ueq as Compared to Neighbors of        C17 Check
PLAT242_ALERT_2_B Low    'MainMol' Ueq as Compared to Neighbors of        C16 Check
PLAT260_ALERT_2_B Large Average Ueq of Residue Including       Cl1      0.314 Check
PLAT420_ALERT_2_B D-H Bond Without Acceptor  O3       --H3       .     Please Check
PLAT990_ALERT_1_B Deprecated .res/.hkl Input Style SQUEEZE Job ...          ! Note
```

Alert level C

```
PLAT220_ALERT_2_C NonSolvent    Resd 1  C   Ueq(max)/Ueq(min) Range        5.8 Ratio
PLAT222_ALERT_3_C NonSolvent Resd 1  H   Uiso(max)/Uiso(min) Range        5.0 Ratio
PLAT230_ALERT_2_C Hirshfeld Test Diff for    C6       --C7       .        5.3 s.u.
PLAT230_ALERT_2_C Hirshfeld Test Diff for    C11      --C12      .        7.0 s.u.
PLAT234_ALERT_4_C Large Hirshfeld Difference C7       --C8       .       0.19 Ang.
PLAT234_ALERT_4_C Large Hirshfeld Difference C15      --C16      .       0.23 Ang.
PLAT241_ALERT_2_C High   'MainMol' Ueq as Compared to Neighbors of        C11 Check
PLAT241_ALERT_2_C High   'MainMol' Ueq as Compared to Neighbors of        C14 Check
PLAT242_ALERT_2_C Low    'MainMol' Ueq as Compared to Neighbors of         C3 Check
```

And 2 other PLAT242 Alerts

More ...

```
PLAT260_ALERT_2_C Large Average Ueq of Residue Including       Eu1      0.109 Check
PLAT342_ALERT_3_C Low Bond Precision on  C-C Bonds ...............    0.01375 Ang.
PLAT410_ALERT_2_C Short Intra H...H Contact  H6       ..H16      .       1.95 Ang.
                                                 x,y,z  =      1_555 Check
PLAT752_ALERT_4_C Angle   Calc      120.01, Rep    120.0(5) ......  Senseless s.u.
              O3      -C3      -C2          1_555  1_555  1_555     #   91 Check
PLAT918_ALERT_3_C Reflection(s) with I(obs) much Smaller I(calc) .         19 Check
PLAT973_ALERT_2_C Check Calcd Positive Resid. Density on       Eu1       1.45 eA-3
PLAT975_ALERT_2_C Check Calcd Resid. Dens.  1.03Ang From O6      .       0.44 eA-3
PLAT992_ALERT_5_C Repd & Actual _reflns_number_gt Values Differ by         19 Check
```

Alert level G

```
FORMU01_ALERT_1_G  There is a discrepancy between the atom counts in the
            _chemical_formula_sum and _chemical_formula_moiety. This is
            usually due to the moiety formula being in the wrong format.
            Atom count from _chemical_formula_sum:   C93 H81 Cl3 Eu1 N9 O21
            Atom count from _chemical_formula_moiety:C93 H81 Cl3 Eu1 N9 O27
PLAT002_ALERT_2_G Number of Distance or Angle Restraints on AtSite         2 Note
PLAT003_ALERT_2_G Number of Uiso or Uij Restrained non-H Atoms ...        11 Report
PLAT007_ALERT_5_G Number of Unrefined Donor-H Atoms .............          2 Report
PLAT042_ALERT_1_G Calc. and Reported MoietyFormula Strings  Differ     Please Check
PLAT072_ALERT_2_G SHELXL First  Parameter in WGHT  Unusually Large       0.19 Report
PLAT172_ALERT_4_G The CIF-Embedded .res File Contains DFIX Records         2 Report
PLAT178_ALERT_4_G The CIF-Embedded .res File Contains SIMU Records         1 Report
PLAT186_ALERT_4_G The CIF-Embedded .res File Contains ISOR Records         2 Report
PLAT244_ALERT_4_G Low    'Solvent' Ueq as Compared to Neighbors of       Cl1 Check
PLAT300_ALERT_4_G Atom Site Occupancy of H3         Constrained at       0.5 Check
PLAT606_ALERT_4_G Solvent Accessible VOID(S) in Structure ........         ! Info
PLAT791_ALERT_4_G Model has Chirality at C6           (Sohnke SpGr)        S Verify
PLAT802_ALERT_4_G CIF Input Record(s) with more than 80 Characters         1 Info
PLAT860_ALERT_3_G Number of Least-Squares Restraints ............         74 Note
PLAT869_ALERT_4_G ALERTS Related to the Use of SQUEEZE  Suppressed         ! Info
PLAT883_ALERT_1_G No Info/Value for _atom_sites_solution_primary .     Please Do !
PLAT910_ALERT_3_G Missing # of FCF Reflection(s) Below Theta(Min).         1 Note
PLAT913_ALERT_3_G Missing # of Very Strong Reflections in FCF ....         1 Note
PLAT933_ALERT_2_G Number of HKL-OMIT Records in Embedded .res File        15 Note
PLAT961_ALERT_5_G Dataset Contains no Negative Intensities .......     Please Check
PLAT978_ALERT_2_G Number C-C Bonds with Positive Residual Density.         0 Info
PLAT996_ALERT_1_G Non-Standard SHELXL LIST 4 Style FCF Supplied ..         ! Check
```

```
  1 ALERT level A = Most likely a serious problem - resolve or explain
  8 ALERT level B = A potentially serious problem, consider carefully
 19 ALERT level C = Check. Ensure it is not caused by an omission or oversight
 23 ALERT level G = General information/check it is not something unexpected

  5 ALERT type 1 CIF construction/syntax error, inconsistent or missing data
 22 ALERT type 2 Indicator that the structure model may be wrong or deficient
  6 ALERT type 3 Indicator that the structure quality may be low
 15 ALERT type 4 Improvement, methodology, query or suggestion
  3 ALERT type 5 Informative message, check
```

It is advisable to attempt to resolve as many as possible of the alerts in all categories. Often the minor alerts point to easily fixed oversights, errors and omissions in your CIF or refinement strategy, so attention to these fine details can be worthwhile. In order to resolve some of the more serious problems it may be necessary to carry out additional measurements or structure refinements. However, the purpose of your study may justify the reported deviations and the more serious of these should normally be commented upon in the discussion or experimental section of a paper or in the "special_details" fields of the CIF. checkCIF was carefully designed to identify outliers and unusual parameters, but every test has its limitations and alerts that are not important in a particular case may appear. Conversely, the absence of alerts does not guarantee there are no aspects of the results needing attention. It is up to the individual to critically assess their own results and, if necessary, seek expert advice.

Publication of your CIF in IUCr journals

A basic structural check has been run on your CIF. These basic checks will be run on all CIFs submitted for publication in IUCr journals (*Acta Crystallographica*, *Journal of Applied Crystallography*, *Journal of Synchrotron Radiation*); however, if you intend to submit to *Acta Crystallographica Section C* or *E* or *IUCrData*, you should make sure that full publication checks are run on the final version of your CIF prior to submission.

Publication of your CIF in other journals

Please refer to the *Notes for Authors* of the relevant journal for any special instructions relating to CIF submission.

PLATON version of 18/05/2022; check.def file version of 17/05/2022

Datablock DFC080sq - ellipsoid plot

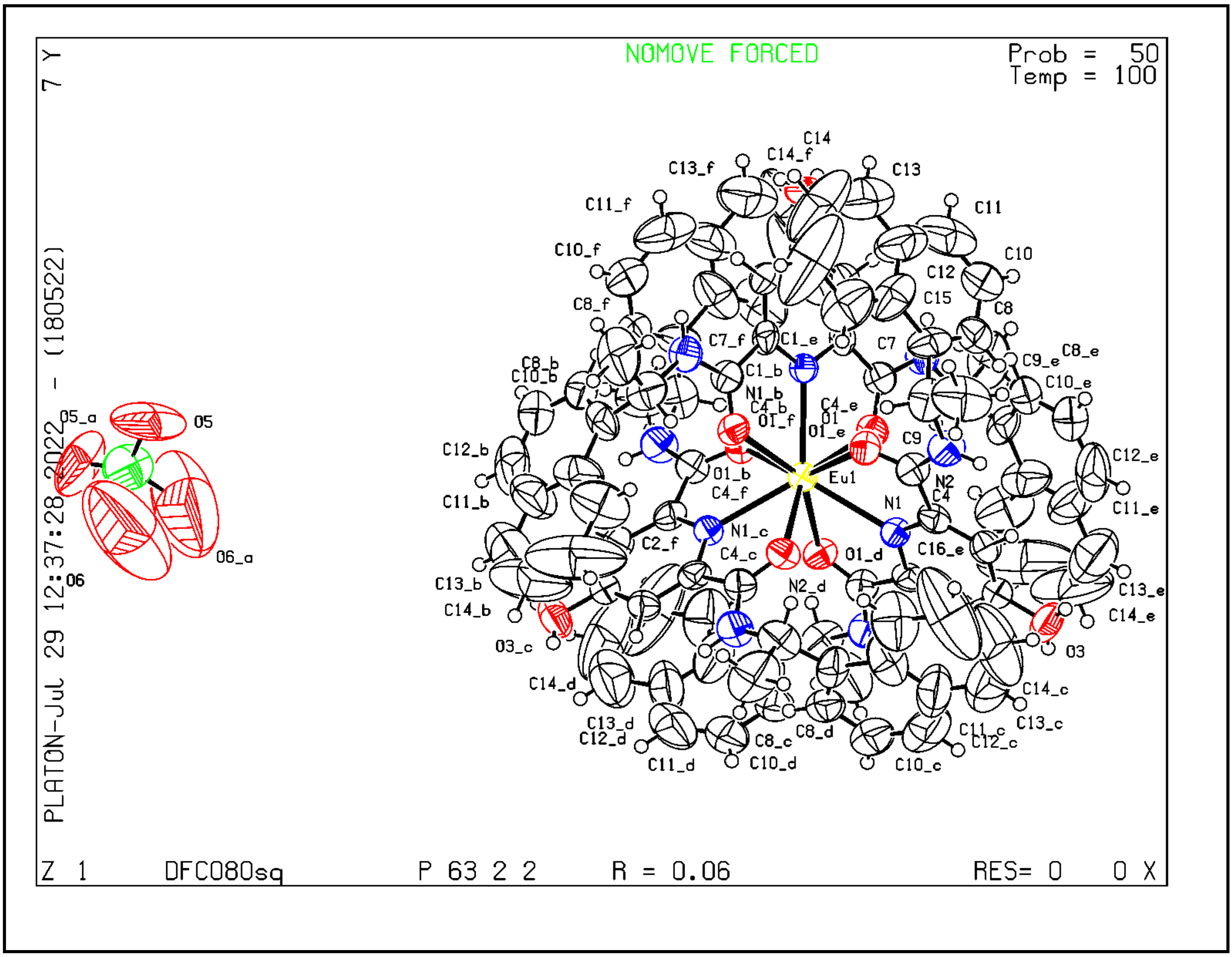

Download CIF editor (publCIF) from the IUCr
Download CIF editor (enCIFer) from the CCDC
Test a new CIF entry
