# Supplementary material for: Ligand Chirality Transfer from Solution State to the Crystalline Self‐Assemblies in Circularly Polarized Luminescence (CPL) Active Lanthanide Systems
Source: Adv Sci (Weinh). 2024 Mar 6;11(18):2307448. doi: 10.1002/advs.202307448 (PMC11095229; doi:10.1002/advs.202307448)
Supplement: Supplementary file 2 — Supporting Information [file ADVS-11-2307448-s001.zip › advs202307448-sup-0002-SuppMat.zip/Checkcif_report_9S.pdf]

checkCIF (basic structural check) running

Checking for embedded fcf data in CIF ...
Found embedded fcf data in CIF. Extracting fcf data from uploaded CIF, please wait . . . .

checkCIF/PLATON (basic structural check)

Structure factors have been supplied for datablock(s) tcd2188

THIS REPORT IS FOR GUIDANCE ONLY. IF USED AS PART OF A REVIEW PROCEDURE FOR PUBLICATION, IT SHOULD NOT REPLACE THE EXPERTISE OF AN EXPERIENCED CRYSTALLOGRAPHIC REFEREE.

No syntax errors found. CIF dictionary
Please wait while processing .... Interpreting this report

Structure factor report

Datablock: tcd2188

Bond precision:	C-C = 0.0145 A		Wavelength=0.71073
Cell:	a=22.8424(2)	b=22.8424(2)	c=12.9269(2)
	alpha=90	beta=90	gamma=120
Temperature:	100 K		

	Calculated	Reported
Volume	5841.29(14)	5841.28(14)
Space group	P 63	P 63
Hall group	P 6c	P 6c
Moiety formula	C102 H81 Eu N9 O9, 3(C F3 O3 S), 0.317(C H Cl3) [+ solvent]	C102 H81 Eu N9 O9, 2.111(C0.15 H0.15 Cl0.45), 3(C F3 O3 S)
Sum formula	C105.32 H81.32 Cl0.95 Eu F9 N9 O18 S3 [+ solvent]	C105.32 H81.32 Cl0.95 Eu F9 N9 O18 S3
Mr	2213.74	2213.73
Dx,g cm-3	1.259	1.259
Z	2	2
Mu (mm-1)	0.689	0.689
F000	2256.7	2257.0
F000'	2258.60	
h,k,lmax	30,30,17	30,30,17
Nref	9703[5062]	9667
Tmin,Tmax	0.937,0.956	0.669,0.746
Tmin'	0.634	

Correction method= # Reported T Limits: Tmin=0.669 Tmax=0.746 AbsCorr = MULTI-SCAN

Data completeness= 1.91/1.00 Theta(max)= 28.313

R(reflections)= 0.0718(8065) wR2(reflections)= 0.2093(9667)

S = 1.041 Npar= 538

The following ALERTS were generated. Each ALERT has the format
test-name_ALERT_alert-type_alert-level.
Click on the hyperlinks for more details of the test.

Alert level B

PLAT987_ALERT_1_B The Flack x is >> 0 - Do a BASF/TWIN Refinement Please Check

Alert level C

PLAT077_ALERT_4_C Unitcell Contains Non-integer Number of Atoms .. Please Check
PLAT220_ALERT_2_C NonSolvent Resd 1 C Ueq(max)/Ueq(min) Range 4.2 Ratio
PLAT245_ALERT_2_C U(iso) H13B Smaller than U(eq) C13B by 0.034 Ang**2
PLAT250_ALERT_2_C Large U3/U1 Ratio for <U(i,j)> Tensor(Resd 2) 2.2 Note
PLAT250_ALERT_2_C Large U3/U1 Ratio for <U(i,j)> Tensor(Resd 3) 2.2 Note
PLAT260_ALERT_2_C Large Average Ueq of Residue Including S6 0.156 Check
PLAT260_ALERT_2_C Large Average Ueq of Residue Including S1 0.156 Check
PLAT342_ALERT_3_C Low Bond Precision on C-C Bonds .... 0.0145 Ang.
PLAT910_ALERT_3_C Missing # of FCF Reflection(s) Below Theta(Min). 5 Note
0 1 0, -1 2 0, 0 2 0, 0 1 1, -1 2 1,
PLAT911_ALERT_3_C Missing FCF Refl Between Thmin & STh/L= 0.600 2 Report
-10 12 0, -1 6 1,
PLAT918_ALERT_3_C Reflection(s) with I(obs) much Smaller I(calc) . 7 Check
PLAT922_ALERT_1_C wR2 in the CIF and FCF Differ by .... 0.0021 Check
PLAT923_ALERT_1_C S Values in the CIF and FCF Differ by .... 0.011 Check
PLAT973_ALERT_2_C Check Calcd Positive Resid. Density on Eu2 1.07 eA-3

Alert level G

PLAT002_ALERT_2_G Number of Distance or Angle Restraints on AtSite 21 Note
PLAT003_ALERT_2_G Number of Uiso or Uij Restrained non-H Atoms ... 66 Report
PLAT007_ALERT_5_G Number of Unrefined Donor-H Atoms .... 1 Report
H
PLAT017_ALERT_1_G Check Scattering Type Consistency of C2 as N
PLAT033_ALERT_4_G Flack x Value Deviates > 3.0 * sigma from Zero . 0.073 Note
PLAT042_ALERT_1_G Calc. and Reported MoietyFormula Strings Differ Please Check
Calc: C102 H81 Eu N9 O9, 3(C F3 O3 S), 0.317(C H Cl3)
Rep.: C102 H81 Eu N9 O9, 2.111(C0.15 H0.15 Cl0.45),
3(C F3 O3 S)
PLAT063_ALERT_4_G Crystal Size Possibly too Large for Beam Size .. 0.66 mm
PLAT072_ALERT_2_G SHELXL First Parameter in WGHT Unusually Large 0.16 Report
PLAT171_ALERT_4_G The CIF-Embedded .res File Contains EADP Records 8 Report
PLAT172_ALERT_4_G The CIF-Embedded .res File Contains DFIX Records 2 Report
PLAT176_ALERT_4_G The CIF-Embedded .res File Contains SADI Records 13 Report
PLAT178_ALERT_4_G The CIF-Embedded .res File Contains SIMU Records 6 Report
PLAT186_ALERT_4_G The CIF-Embedded .res File Contains ISOR Records 4 Report
PLAT187_ALERT_4_G The CIF-Embedded .res File Contains RIGU Records 5 Report
PLAT191_ALERT_3_G A Non-default SADI Restraint Value has been used 0.0100 Report
And 6 other PLAT191 Alerts
More ...
PLAT231_ALERT_4_G Hirshfeld Test (Solvent) S6 --O2 . 9.7 s.u.
And 3 other PLAT231 Alerts
More ...
PLAT300_ALERT_4_G Atom Site Occupancy of O13B Constrained at 0.6667 Check
And 15 other PLAT300 Alerts
More ...
PLAT301_ALERT_3_G Main Residue Disorder ....(Resd 1) 60% Note
PLAT302_ALERT_4_G Anion/Solvent/Minor-Residue Disorder (Resd 2) 100% Note
And 3 other PLAT302 Alerts
More ...
PLAT304_ALERT_4_G Non-Integer Number of Atoms in ..... (Resd 2) 6.55 Check
And 3 other PLAT304 Alerts
More ...
PLAT398_ALERT_2_G Deviating C-O-C Angle From 120 for O13A . 164.1 Degree
PLAT605_ALERT_4_G Largest Solvent Accessible VOID in the Structure 610 A**3
PLAT720_ALERT_4_G Number of Unusual/Non-Standard Labels .... 18 Note
C2 C1EB H1EB C1FB H1FB Ha Hb Hc
C1KB C1DB H1DB C1EA H1EA C1FA H1FA C1KA
C1DA H1DA
PLAT811_ALERT_5_G No ADDSYM Analysis: Too Many Excluded Atoms .... ! Info
PLAT860_ALERT_3_G Number of Least-Squares Restraints .... 1601 Note
PLAT868_ALERT_4_G ALERTS Due to the Use of _smtbx_masks Suppressed ! Info
PLAT912_ALERT_4_G Missing # of FCF Reflections Above STh/L= 0.600 10 Note
PLAT913_ALERT_3_G Missing # of Very Strong Reflections in FCF .... 2 Note
0 1 0, 0 1 1,
PLAT930_ALERT_2_G FCF-based Twin Law (1 0 0) Est.d BASF 0.46 Check
PLAT931_ALERT_5_G CIFcalcFCF Twin Law (1 0 0) Est.d BASF 0.48 Check

PLAT933_ALERT_2_G Number of HKL-OMIT Records in Embedded .res File 6 Note
-10 12 0, -1 2 -1, -1 2 1, -1 6 -1, -1 6 1, 0 2 0,
PLAT969_ALERT_5_G The 'Henn et al.' R-Factor-gap value .... 10.24 Note
Predicted wR2: Based on SigI**2 2.02 or SHELX Weight 20.70
PLAT978_ALERT_2_G Number C-C Bonds with Positive Residual Density. 3 Info

---

0 **ALERT level A** = Most likely a serious problem - resolve or explain
1 **ALERT level B** = A potentially serious problem, consider carefully
14 **ALERT level C** = Check. Ensure it is not caused by an omission or oversight
63 **ALERT level G** = General information/check it is not something unexpected

5 ALERT type 1 CIF construction/syntax error, inconsistent or missing data
14 ALERT type 2 Indicator that the structure model may be wrong or deficient
14 ALERT type 3 Indicator that the structure quality may be low
41 ALERT type 4 Improvement, methodology, query or suggestion
4 ALERT type 5 Informative message, check

It is advisable to attempt to resolve as many as possible of the alerts in all categories. Often the minor alerts point to easily fixed oversights, errors and omissions in your CIF or refinement strategy, so attention to these fine details can be worthwhile. In order to resolve some of the more serious problems it may be necessary to carry out additional measurements or structure refinements. However, the purpose of your study may justify the reported deviations and the more serious of these should normally be commented upon in the discussion or experimental section of a paper or in the "special_details" fields of the CIF. checkCIF was carefully designed to identify outliers and unusual parameters, but every test has its limitations and alerts that are not important in a particular case may appear. Conversely, the absence of alerts does not guarantee there are no aspects of the results needing attention. It is up to the individual to critically assess their own results and, if necessary, seek expert advice.

Publication of your CIF in IUCr journals

A basic structural check has been run on your CIF. These basic checks will be run on all CIFs submitted for publication in IUCr journals (*Acta Crystallographica*, *Journal of Applied Crystallography*, *Journal of Synchrotron Radiation*); however, if you intend to submit to *Acta Crystallographica Section C* or *E* or *IUCrData*, you should make sure that full publication checks are run on the final version of your CIF prior to submission.

Publication of your CIF in other journals

Please refer to the *Notes for Authors* of the relevant journal for any special instructions relating to CIF submission.

PLATON version of 13/12/2023; check.def file version of 13/12/2023

Datablock tcd2188 - ellipsoid plot

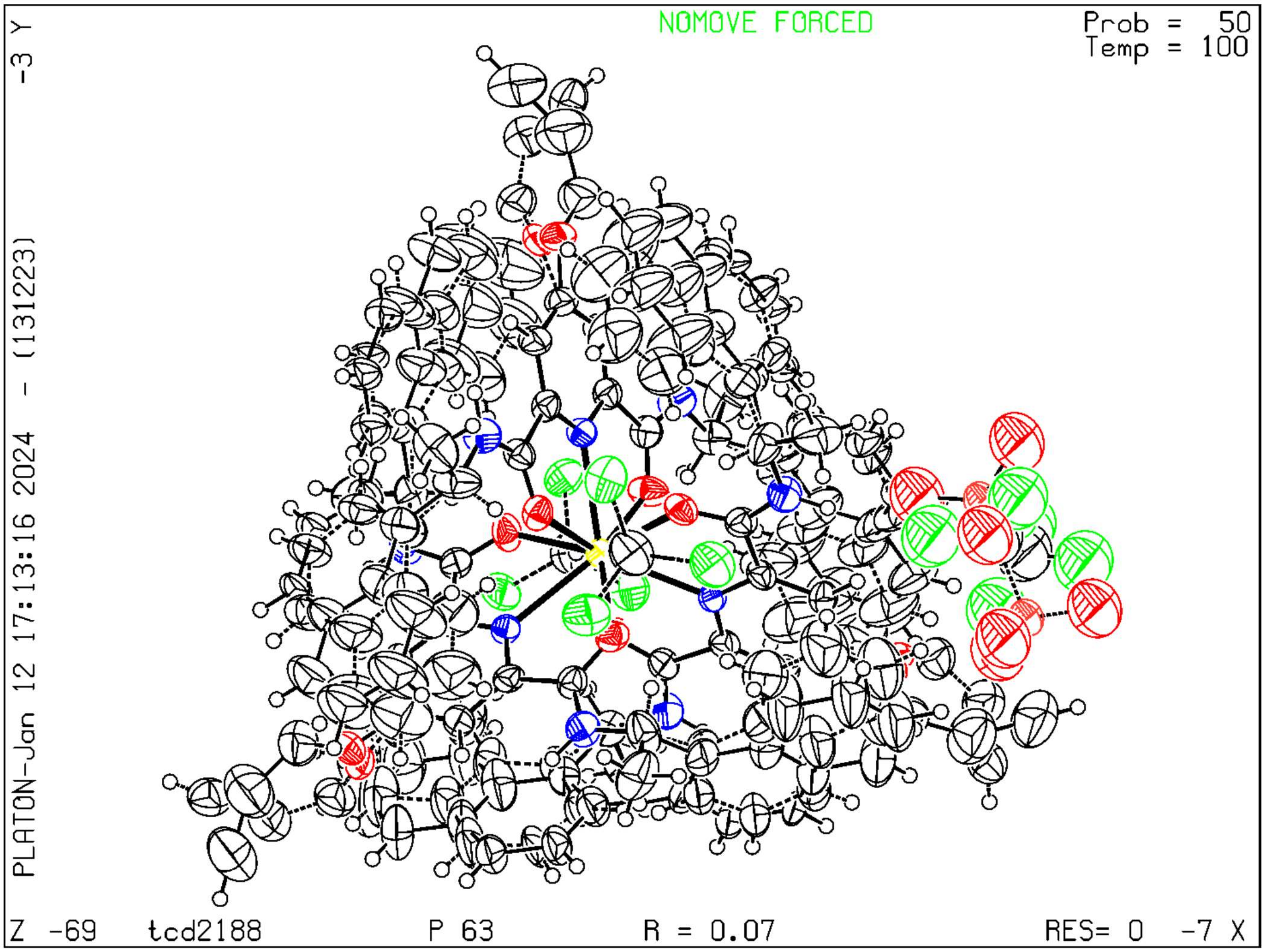

Download CIF editor (publCIF) from the IUCr
Download CIF editor (enCIFer) from the CCDC
Test a new CIF entry
